# Using pseudoalignment and base quality to accurately quantify microbial community composition

**DOI:** 10.1371/journal.pcbi.1006096

**Published:** 2018-04-16

**Authors:** Mark Reppell, John Novembre

**Affiliations:** Department of Human Genetics, University of Chicago, Chicago, Illinois, United States of America; Helmholtz-Zentrum fur Infektionsforschung GmbH, GERMANY

## Abstract

Pooled DNA from multiple unknown organisms arises in a variety of contexts, for example microbial samples from ecological or human health research. Determining the composition of pooled samples can be difficult, especially at the scale of modern sequencing data and reference databases. Here we propose a novel method for taxonomic profiling in pooled DNA that combines the speed and low-memory requirements of k-mer based pseudoalignment with a likelihood framework that uses base quality information to better resolve multiply mapped reads. We apply the method to the problem of classifying 16S rRNA reads using a reference database of known organisms, a common challenge in microbiome research. Using simulations, we show the method is accurate across a variety of read lengths, with different length reference sequences, at different sample depths, and when samples contain reads originating from organisms absent from the reference. We also assess performance in real 16S data, where we reanalyze previous genetic association data to show our method discovers a larger number of quantitative trait associations than other widely used methods. We implement our method in the software Karp, for k-mer based analysis of read pools, to provide a novel combination of speed and accuracy that is uniquely suited for enhancing discoveries in microbial studies.

## Author summary

Pooled DNA from multiple unknown organisms arises in a variety of contexts. Determining the composition of pooled samples can be difficult, especially at the scale of modern data. Here we propose the novel method Karp, designed to perform taxonomic profiling in pooled DNA. Karp combines the speed and low-memory requirements of k-mer based pseudoalignment with a likelihood framework that uses base quality information to better resolve multiply mapped reads. We apply Karp to the problem of classifying 16S rRNA reads using a reference database of known organisms. Using simulations, we show Karp is accurate across a variety of read lengths, reference sequence lengths, sample depths, and when samples contain reads originating from organisms absent from the reference. We also assess performance in real 16S data, where we reanalyze previous genetic association data to show that relative to other widely used quantification methods Karp reveals a larger number of microbiome quantitative trait association signals. Modern sequencing technology gives us unprecedented access to microbial communities, but uncovering significant findings requires correctly interpreting pooled microbial DNA. Karp provides a novel combination of speed and accuracy that makes it uniquely suited for enhancing discoveries in microbial studies.

This is a *PLOS Computational Biology* Methods paper.

## Introduction

The study of microbial community composition has been revolutionized by modern genetic sequencing. Experimenters can forgo the laborious work of culturing cells and detect a broader range of taxa than was previously possible. This improved ability to describe the microbes present in a pooled sample has led to important findings in human health [1–3] and ecology [4, 5]. These findings rely on quantification of the taxa present in experimental samples, and towards that goal many methods have been developed. The ever-increasing scale of both sequencing data and relevant reference databases require that such methods be efficient in addition to accurate. Here we present a novel method, Karp, which combines the speed of k-mer-based pseudoaligning with a likelihood framework that incorporates base quality information. In this work we use Karp to profile the taxonomy of pooled 16S microbiome data quickly and with an accuracy superior to widely adopted alternative methods.

Microbiome samples are commonly generated using either shotgun sequencing or the sequencing of marker genes, most often the gene encoding 16S ribosomal RNA. Classifying the output of shotgun sequencing can be difficult, as limited reference databases exist for entire bacterial genomes, so whole genome sequencing generally either requires computationally intensive de novo assembly methods [6–8] or limits the range of organisms available for study [9]. Alternatively, several large reference databases exist for microbial 16S sequences [10–12]. The 16S gene contains alternating regions of highly conserved and highly variable sequences, making it easy to target and well powered for differentiating taxa. Many experiments target one or several of the 16S hypervariable regions and sequence to a high depth [13–15].

Sequence identification problems can be broadly classified as either open-reference or closed-reference. In open-reference problems the reference sequences of possible contributors are unknown, so sample sequences are clustered or binned based on similarities between them. In a closed-reference problem the sequences of contributors are known, and quantification is typically a process of matching the observed sequencing reads against a reference database in order to build a taxonomic profile of a sequenced sample. Some algorithms use a hybrid approach, with a binning step followed by a closed-reference classification. Open and closed reference methods are suited for answering different research questions. While open reference methods can uncover novel organisms and broadly classify diversity in samples, closed reference methods provide information that can help compare the abundance of specific organisms in samples and search for associations when differences are observed. There are several significant challenges faced by closed-reference methods for classifying microbial samples. First, methods must be able to provide unbiased estimates when samples contain previously unidentified taxa. Second, microbial samples often contain a range of genetic diversity unmatched by single species sequencing samples. And finally, methods must efficiently compare sequences against reference databases containing potentially millions of organisms.

One widely applied approach to classifying microbiome samples is to calculate a similarity metric between a sample’s reads themselves, between the reads and a reference database, or both. Many early similarity based methods first employed the Basic Local Alignment Search Tool (BLAST) [16], which calculates both a similarity score and relative significance for local alignments of queries against reference sequences. Several methods refined BLAST output to classify sequence origin [17–19], however, the BLAST algorithm is computationally very intensive, making methods based on it hard to scale with both reference panel size and sequencing depth. These early BLAST based methods have largely been superseded by the USEARCH and UCLUST algorithms [20, 21] and several other recent similarity-based clustering algorithms [22–25] that are fast enough to handle modern data (millions of reads, each one hundreds of base pairs long). The speed and accuracy of these modern clustering algorithms has been shown to be very similar [22, 26]. Another approach for classifying sequences, often with the goal of profiling a sample, is based on the shared phylogeny of samples, and places query sequences along a phylogenetic tree. Phylogenetic methods using maximum-likelihood estimation [27], Bayesian posterior probabilities [28], or neighbor-joining [29] have all been developed. While representing the explicit relationships between organisms provided by phylogenetic methods is attractive, these methods impose a large computational burden. Also, while they often make accurate taxonomic assignments, phylogenetic methods tend to suffer from low sensitivity [30]. A third approach to classifying microbiome sequencing reads consists of methods that use sequence composition. Early sequence composition methods calculated the probability of a query originating from a specific taxon based on shared k-mers [31–33], an approach that has been refined and expanded upon by recent sequence composition methods [34, 35]. Recent articles by Lindgreen *et al* (2016) [36] and Sczyrba *et al* (2017) [37] provide performance evaluations of both binning and profiling methods in the context of metagenomics.

Very recently, the development of pseudoalignment [38] has allowed sequence composition quantification with minimal computational requirements and an accuracy superior to other recent methods [39, 40]. Pseudoaligning, originally developed in the context of RNA sequencing experiments, is a rapid k-mer based algorithm that uses a de Bruijn Graph of the reference database to identify potential matches for a query sequence without aligning the query to reference sequences. Pseudoaligning is very fast, and is implemented in the software Kallisto [38], which uses an expectation maximization (EM) algorithm to resolve multiply-mapped reads without assigning them to a single taxonomic unit. The speed advantages of Kallisto and pseudoaligning come at a cost; notably it ignores information about sequencing quality that could help assign multiply-mapped reads more accurately. Sequencing errors occur non-uniformly along reads, and base-quality scores record the probability of errors at each base. Thus, quantification can be improved by using base-quality scores to help distinguish true mismatches between reads and references from sequencing errors.

Kallisto’s limitations led us to develop Karp, a program that leverages the speed and low memory requirements of pseudoaligning with an EM algorithm that uses sequencing base-quality scores to quickly and accurately classify the taxonomy of pooled microbiome samples. Here, we demonstrate with simulations of 16S sequencing experiments the improvement in accuracy that Karp provides relative to Kallisto, modern similarity-based methods, the machine learning based 16S Classifier [41], and the Wang *et al.* (2007) naive Bayesian classifier. We also use simulations to demonstrate how Karp leads to better estimates of important summary statistics and remains robust when sequences from organisms absent from our reference database are present at high frequencies in samples. We also introduce a modified version of the Karp algorithm that collapses information across reference sequences sharing the same taxonomic labels, which we call Karp-Collapse. We evaluated the method in practice by assessing the number of discoveries made in a dataset [42] designed to associate host microbiome abundance with individual’s genotypes. We tested genera level abundances, and found that across sampling conditions Karp is able to detect more significant associations than comparison methods. For researchers seeking to maximize their power to detect associations between known taxa in their 16S samples and environmental or genetic variables, Karp provides the highest accuracy at a minimal comupational cost.

## Materials and methods

### Ethics statement

The human data we reanalyzed in this study was originally collected and analyzed by the Ober Lab, who had their protocol approved by the University of Chicago IRB (protocol 09-354-A). Written informed consent was obtained from all adult participants and the parents of minors. In addition, written assent was obtained from minor participants.

### Method overview

**Fig 1:**
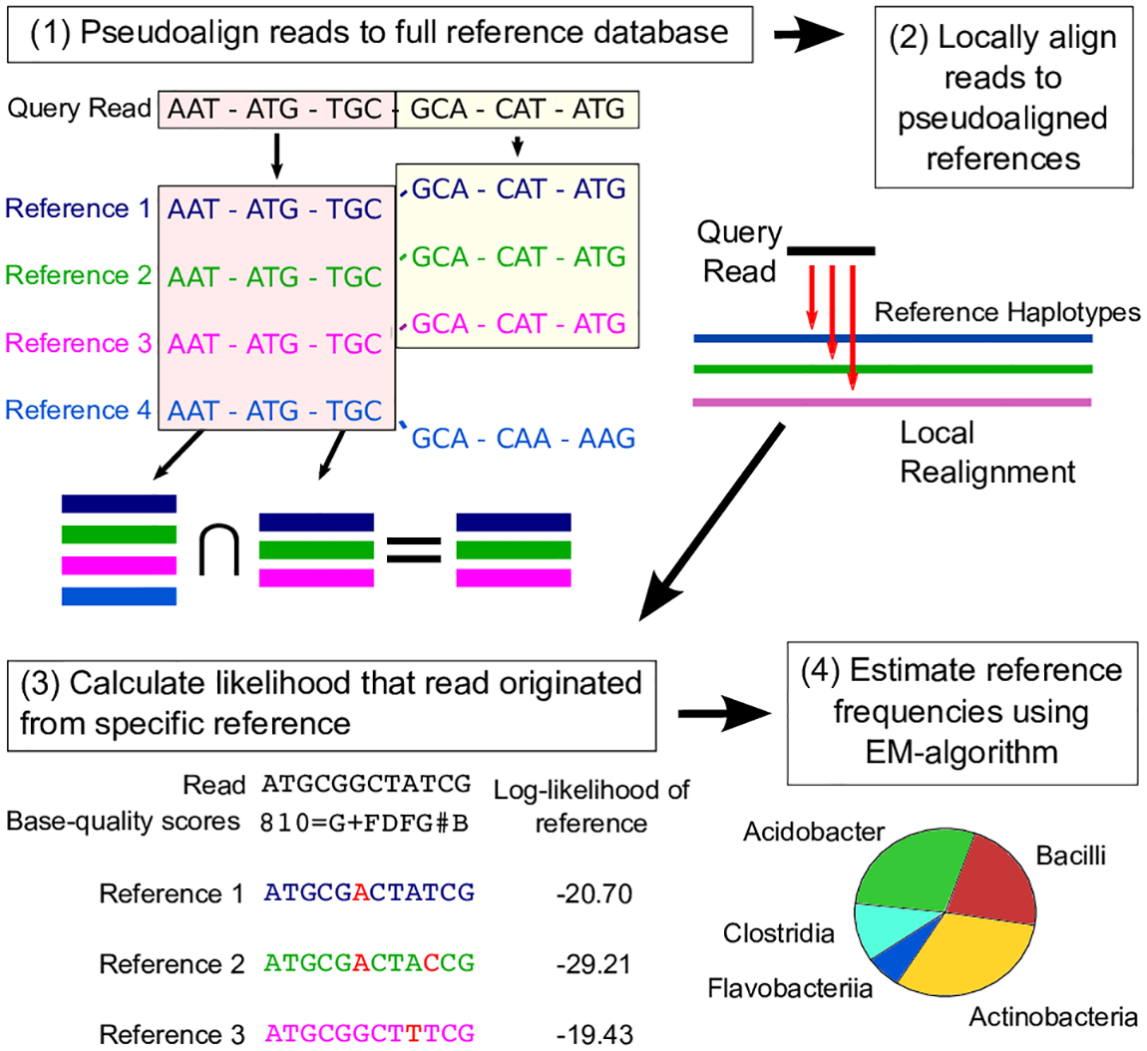
Overview of Karp. (1) Query reads are pseudoaligned against an index of the reference database, resulting in a set of references they could have potentially originated from. (2) The query reads are locally aligned to the possible references. (3) Using the best alignment, the likelihood that a read originated from a specific reference is calculated. (4) Using the read likelihoods an EM-algorithm is employed to estimate the relative abundances of the references in the pool of query reads.

The aim of Karp is to estimate a vector $F=(f_{1},\ldots f_{M})$, containing the proportion of a pooled DNA sample that is contributed by each of *M* possible haploid reference sequences, which we refer to as just reference sequences hereafter. Fig 1 gives an outline of Karp’s quantification process and Table 1 defines the terms used in this section. The first step in using Karp is the construction of a k-mer index of the *M* reference sequences. This index catalogs the subset of the *M* reference sequences that contain each unique k-mer of a given length. Next, the query reads are pseudoaligned using the k-mer index. Query reads that pseudoalign to multiple references (multiply-mapped reads) are locally aligned to each potential reference, and each reference’s best alignment is kept. Queries that pseudoalign to a single reference are assigned without alignment. Next, for multiply-mapped reads the likelihood that they originated from each potential reference is calculated using the best alignment and the base-quality scores that correspond to the read. After the likelihoods for every query read have been calculated, an EM-algorithm is used to estimate the relative frequencies of each reference sequence contributing to the pool. More details about the method are provided in the following sections.

**Table 1:** Materials and methods definitions.

	Definitions
*N*	total number of reads
*M*	total number of haploid reference sequences
*r*_*j*_	read *j* for *j* ∈ 1, .., *N*
*h*_*k*_	haploid reference sequence *k* for *k* ∈ 1, …, *M*
F	vector of length *M*, with entries *f*_*k*_ corresponding to frequency of reference *h*_*k*_
*η*_*j*_	vector of length *M*, entry *k* = 1 if read *r*_*j*_ originated from reference *h*_*k*_, 0 otherwise
*l*_*j*,*k*_	*P*(*r*_*j*_|*η*_*j*,*k*_ = 1), likelihood of read *r*_*j*_ originating from reference *h*_*k*_
*t*_*k*_	number of reads known to originate from reference *h*_*k*_
N*	$N-\sum_{k=1}^{M}t_{k}$, number of reads with unknown reference of origin

### Pseudoaligning and alignment

Aligning millions of reads against hundreds of thousands of references is impractical in both memory and time. However, calculating the probability that a read originated from a given reference sequence using base-quality information requires an alignment. To overcome this challenge, Karp uses pseudoalignment as a filter before performing local alignment. Pseudoalignment is a fast and memory efficient way to narrow the space of possible references from which a query read may have originated. Our pseudoaligning algorithm is directly based on that of Kallisto [38]. Briefly, first an indexed de Bruijn Graph of the reference database is constructed, with each observed k-mer mapped to an equivalence class of reference sequences that it is contained in. Next, each query read is decomposed into its constituent k-mers, which are searched against the index. Kallisto uses a strict intersection of the equivalence classes returned by the k-mer search to arrive at a pseudoalignment. Karp can also be set to use the strict intersection of equivalence classes. However, because mismatched bases are accounted for in Karp’s read likelihood framework, we are more concerned with false negatives than false positive matches and the default setting is more inclusive. In Karp’s default mode, if no strict intersection is observed, the intersection of all equivalence classes with the same maximum number of matched k-mers, conditional on the maximum being >1, are declared matches. Reads with <2 matched k-mers are always removed from analysis for failing to pseudoalign.

After pseudoaligning, reads are locally aligned to the matching reference sequences using the Striped Smith-Waterman algorithm [43, 44] (SSW penalties: mismatch (2), gap opening (3), gap extending (1)).

### Read likelihoods

Our likelihood and EM frameworks build closely on the work of Kessner *et al.* (2013) [45], whose software Harp implemented a method for estimating reference frequencies in pooled DNA. Kessner *et al.* (2013) recognized the potential of their method to improve accuracy in microbiome studies, but Harp was computationally infeasible with modern reference databases.

For a read *r*_*j*_ with *j* ∈ 1, .., *N* and length *L*_*j*_, let (*r*_*j*_[1], …, *r*_*j*_[*L*_*j*_]) be the base calls at each position along the read. Assume we have a reference database with *M* possible reference sequences. For reference sequence *h*_*k*_ with *k* ∈ 1, …, *M* let (*h*_*k*,*j*_[1], …, *h*_*k*,*j*_[*L*_*j*_]) give the values of the bases in *h*_*k*_ corresponding to the best alignment of read *r*_*j*_. Note the entries in this vector may not be contiguous due to insertions, deletions, or because the reads are paired-end. Define the probability of sequencing error at each position as *q*_*j*_[*i*] = *P*(*r*_*j*_[*i*] ≠ *h*_*k*,*j*_[*i*]) for *i* ∈ 1, .., *L*_*j*_, and define the variable *η*_*j*_, a vector of length *M* with components *η*_*j*,*k*_ = 1 if *r*_*j*_ originated from reference *h*_*k*_ and 0 otherwise. Assuming sequencing errors are independent, we can then formulate the probability of read *r*_*j*_ arising from reference *h*_*k*_, which we label *l*_*j*,*k*_, as

$$\begin{array}{c} l_{j,k}=P\left(r_{j}|\eta_{j,k}=1\right)=\prod_{i=1}^{L_{j}}P\left(r_{j}\left[i\right]|h_{k,j}\left[i\right],q_{j}\left[i\right]\right) \end{array}$$ (1)

where, if we assume every base is equally likely when an error occurs

P(rj[i]|hk,j[i],qj[i])={1-qj[i]llllllifrj[i]=hk,j[i]qj[i]/3lmslllifrj[i]≠hk,j[i]. (2)

A more complete definition of the probability would sum over all possible alignments of *r*_*j*_ to *h*_*k*_. However, in non-repetitive marker gene sequence the best local alignment typically contributes such a large proportion of the probability weight, that excluding alternate local alignments has a negligible impact on results but substantially improves computation.

### Estimating reference sequence frequencies

As previously noted, the aim of our method is to estimate a vector $F=(f_{1},\ldots ,f_{M})$, containing the frequencies of the *M* possible references in a pooled DNA sample. If we were to observe which reference gave rise to each read in our sample, the maximum likelihood estimate of F, $\hat{F}$, would follow directly from the multinomial likelihood. In reality, we observe the reads *r*, but the references that they originate from, *η*, are unobserved. To estimate F we therefore employ an EM algorithm, with a form common to mixture model problems. Details of our EM algorithm are provided in S1 File.

Karp modifies the standard mixture EM algorithm in two ways to speed up performance. The first is an assumption that if a read *r*_*j*_ uniquely pseudoaligns to a reference *h*_*k*_ then $P(\eta_{j,k}=1|r,F)=1$. For haploid reference *h*_*k*_, label the number of reads that uniquely map as *t*_*k*_ and define $N^{*}=N-\sum_{k=1}^{M}t_{k}$. Then we can write the likelihood of the data as

$$\begin{array}{c} L(F|\eta ,r)\propto \prod_{k=1}^{M}f_{k}^{\left(t_{k}+\sum_{j=1}^{N*}\eta_{j,k}\right)} \end{array}$$ (3)

and our update step as

$$\begin{array}{c} {\hat{f}}_{k}^{\left(i+1\right)}=\frac{t_{k}}{N}+\frac{1}{N}\sum_{j=1}^{N^{*}}[\frac{l_{j,k}{\hat{f}}_{k}^{\left(i\right)}}{\sum_{m=1}^{M}l_{j,m}{\hat{f}}_{m}^{\left(i\right)}}]. \end{array}$$ (4)

This assumption also provides a logical initial estimate of $F^{\left(0\right)}$

$$\begin{array}{c} f_{k}^{\left(0\right)}=\frac{t_{k}}{N}+\frac{N^{*}}{M}. \end{array}$$ (5)

The second speed-up that Karp uses is an implementation of SQUAREM [46], which accelerates the convergence of EM algorithms by using information from multiple previous parameter updates to improve the current EM update step.

Additionally, Karp allows the user to specify a minimum reference frequency. After the frequency of a reference falls below this threshold during the EM updates, its value is set to zero and its frequency weight is distributed evenly across the remaining references. Technically, this filter violates the guarantee of the EM algorithm to reach a local maximum of the likelihood function, however, it proves quite useful in practice. We find that when there is sufficient information to distinguish closely related species this approach imposes a sparsity condition which is effective for avoiding the estimation of spurious references at very low frequencies. When only limited information to distinguish between closely related species exists, for example in data generated from a single 16S hypervariable region, it can be better to set the minimum frequency very low to avoid eliminating true low frequency taxa with probability weights distributed evenly across indistinguishable taxa. S2 and S3 Figs explore the impact of different thresholds on the simulated and real data presented in this study.

### Read likelihood filter

Our EM method relies on the fact that all the reads in our sample originated from reference sequences present in our reference database. In real data this assumption can be problematic; the classification of microbial taxonomy is an ongoing project and many taxons have yet to be identified or referenced. To preserve the accuracy of our frequency estimates in the presence of reads originating from organisms absent from the reference database we implemented a filter on the maximum read likelihood value [45].

Specifically, using the base-quality scores of the query reads we calculate a “null” distribution of likelihood values corresponding to what we would observe if every query were matched to its true originating reference and every mismatched base was the result of sequencing errors. Then, after the local realignment step we filter out query reads where the greatest observed likelihood falls too far outside this distribution, as these are unlikely to truly match any of the reference sequences present in the database. Karp includes the option to output the maximum likelihood for each read, which can be used to determine the appropriate cutoff value. In our simulations, where a variety of empirical quality score distributions were encountered, cutoff values between -3.0 and -1.5 yielded similar results, a finding in line with Kessner *et al.* (2013), and which supports a default value of -2.0. For more details about the filter see S2 File.

This likelihood filter works in concert with the pseudoalignment step to prevent chimeric reads that may exist in a sample from being classified. Chimeric reads should rarely pseudo align properly and when they do, the likelihood filter can trigger their removal as the alignment is likely to contain mismatches.

### Karp-Collapse

The default approach in Karp estimates the relative frequencies of the individual reference sequences present in the reference database. In many microbiome databases there is not a one-to-one relationship between reference sequences and taxonomic labels; multiple sequences share a single label. When little information exists to distinguish closely related reference sequences, estimating the relative frequencies at the taxon level rather than individual reference sequences can improve accuracy. To accommodate this, Karp includes a collapse option, which adds a step to the estimation procedure. When the collapse option is used, after pseudoalignment and local alignment Karp calculates the average likelihood for each taxonomic label, and uses these likelihoods in the EM algorithm to estimate taxonomic frequencies. This can be interpreted in a Bayesian context as the likelihood a read is from a taxon under a uniform prior for the source reference sequence within that taxa. Karp output in collapse mode provides counts at each taxonomic level from species to phylum. Because it is estimating the frequencies of fewer categories, collapse mode is often faster than Karp’s default. It is important to note that the read-likelihood distribution for the read filter described in section Read likelihood filter, is based on the assumption of matching a read with its exact source. Karp-Collapse potentially combines information from multiple reference sequences, so this distribution no longer fits the data, and the filter should not be applied in the same manner. For more details see S3 File.

### Simulating 16S reads

To compare Karp with alternative methods we simulated pooled sequence samples. The general simulation procedure was as follows. First, a fixed number of reference sequences were selected at random from a reference fasta file and a vector of frequencies corresponding to these references was generated by drawing from a Dirichlet distribution. Next, a predetermined number of reads were simulated. For each read a reference sequence was drawn at random according to its frequency in the original frequency vector. We simulated reads from either the entire 16S gene or from only the V4 hypervariable region. When we simulated reads from the entire gene a read start position was selected uniformly and a number of bases corresponding to the desired read length were copied from the selected reference. In the case of paired-end reads, the distance between pairs was drawn as an upper-bound Poisson random variable with an empirically derived mean. Bases which would cause the read to extend past the end of the reference were excluded from being initiation points. When we simulated paired-end reads from the V4 hypervariable region, the initiation point for the forward read was the first base of the region, and the reverse read sequenced backwards from the region’s final base. Once a read’s bases were copied, a corresponding base-quality score vector was generated based on an empirical distribution of quality scores. To simulate 75bp single-end reads we used the publicly available Illumina-sequenced mock-community dataset from the Human Microbiome Project [47]. For simulating 151bp paired-end reads we used the quality scores observed in Illumina-sequenced microbiome samples collected from Amish and Hutterite mattresses [48]. Finally, 301bp paired-end reads were simulated using scores from a sample of human saliva downloaded from Illumina’s BaseSpace platform (https://basespace.illumina.com/projects/17438426) [49]. Finally, errors were simulated along the read with probabilities corresponding to the base-quality score at each position and assuming that the three alternative bases were equally likely. After adding errors the read was added to the pooled sample, and the algorithm proceeded to the next read.

### Simulations

In our simulations we used two reference databases. For full 16S gene sequence we used the 99% identity GreenGenes version 13.8 [12]. To simulate reads from the V4 hypervariable region we used the TaxMan tool [50] with the primers from Caporaso *et al* (2012) [51] to extract the region from the Greengenes 13.5 database. We simulated reads as coming from only the V4 region, but used the database with the full gene sequence for classification. We reduced the full gene database to match the non-redundant sequences present in the V4 database so that we could classify which taxon a read was generated from. We simulated samples with two depths: first, samples containing 10^6^ sequencing reads, a depth inspired by recent high-depth studies [48] and designed to demonstrate the computational feasibility of Karp, and second, samples with 10^5^ reads, more in line with more shallow pooled sequencing experiments [52, 53]. At both depths, for each sample we selected 1,000 reference sequences randomly from the reference database and simulated reads following the approach in the section “Simulating 16S reads”. The Dirichlet distribution used to generate the sample frequency vectors had identical alpha values varied between 0.002 and 7. These parameter settings created samples with a broad range of Shannon Diversity values (S4 Fig). For each combination of read length, region size, and sample depth we generated between 100 and 200 samples. We placed samples into bins based on their Shannon diversity, and each bin for each condition had a minimum of 10 samples. Each sample was a unique mix of 1,000 reference sequences. With Kallisto and Karp the raw forward and reverse reads were directly classified. For SINTAX, SortMeRNA, 16S Classifier, Mothur’s Naive Bayesian classifier, UCLUST, and USEARCH6.1 paired-end reads were merged into contigs using the QIIME script *join_paired_ends.py* or Mothur’s *make.contigs* command before being submitted for classification. The QIIME algorithms UCLUST and USEARCH6.1 performed OTU clustering before taxonomy was assigned, and as would be expected observed an increasing number of clusters as diversity increased. At the high end, in the 75bp single-end samples with 1,000,000 reads we observed between 10,000 and 70,000 OTUs in the samples. On the low end, in the 301bp paired-end 100,000 read samples we observed between 25 and 2,000 OTUs.

We simulated an additional 100 samples with 75bp single-end reads to compare how each method’s frequency estimates impacted the estimation of common sample summary statistics. Many statistics, such as *β* Diversity, summarize the sharing of taxa between samples, so instead of 1,000 unique taxa in each sample, we used a shared pool of 1,000 taxa for all 100 samples, and further increased the similarity between samples by introducing correlation between the reference frequencies. The reference frequencies for each sample were a linear combination of a random Dirichlet variable generated in a manner identical to the simulations above and the reference frequencies of the preceding sample. In this way the samples again covered the full range of Shannon Diversity values, however the frequencies of shared taxa was potentially much higher, providing a broader range of summary statistic values in the simulations.

Next we compared how the methods performed when the simulated samples contained reads generated from taxa that were absent from the reference database being used for quantification. We selected one phylum (*Acidobacteria*), one order (*Pseudomonadales*), and one genus (*Clostridiisalibacter*) at random from the taxa in GreenGenes with more than 30 reference sequences. Then, for each missing taxa, we simulated 10 samples where 50% of the reads originated from 3 different members and at least 5% of the reads came from closely related taxa (kingdom Bactera for *Acidobacteria*, class Gammaproteobacteria for *Pseudomonadales*, and family Clostridiaceae for *Clostridiisalibacter*). Next, we create 3 reduced GreeGenes reference databases, each with one of the missing taxa (including all lower ranking members) expunged. Finally, we classified the simulated samples using both the appropriate reduced reference database and the full GreenGenes database.

Finally, we examined how sensitive our results were to the assumption that base-quality scores are accurate representations of the probability of sequencing error. Karp assumes that base-quality scores follow the Phred scale, where the probability of a sequencing error is $10^{\frac{Q}{-10}}$ for quality score *Q*. To test this assumption we simulated and classified 50 samples where the actual probability of an error was $10^{\frac{Q}{-5}}$ and also 50 samples where errors occurred uniformly at 1% of bases.

We compare the different methods using an AVGRE (AVerage Relative Error) metric [39, 54, 55] which is based on the absolute value of the difference between the true and estimated counts of reads in the simulated samples. Define *M*_*a*_ as a set of indices for the actual reference sequences contributing to a pooled sample and *M*_*e*_ as the set of indices for additional references a method classifies as having a non-zero number of reads that are not truly present. Also, let *T*_*i*,*e*_ be the count of reads estimated for reference *i* and *T*_*i*,*a*_ is the actual number of simulated reads from reference *i* present in the sample. Using these values the AVGRE metric has the form:

$$\begin{array}{c} AVGRE=\frac{1}{1000}\sum_{i\in M_{a}\cup M_{e}}|T_{i,e}*\frac{\sum_{i\in M_{a}}T_{i,a}}{\sum_{i\in M_{a}\cup M_{e}}T_{i,e}}-T_{i,a}| \end{array}$$ (6)

We include the scaling factor of 1/1000 in order to transform the value into an estimate of the average per-reference error rate, as our pooled simulation samples include 1,000 individual reference sequences. We use the same scaling factor when looking at errors in the estimation of higher order taxonomy for consistency, although the true number of references at any given taxonomic level will be <1,000. Throughout this work we calculate error using only references with either estimated or true frequencies >0.1%.

### Real data

To test the performance of Karp with real data we reanalyzed samples originally published by Igartua *et al.* (2017). In brief, these samples were collected from nasal brushings of Hutterites located in South Dakota at two time points, January/February 2011 (winter) and July 2011 (summer). Two nasal sites were collected, the nasal vestibule and the nasopharynx. After quality control a total of 332 samples were analyzed (87 summer and 80 winter vestibule, 88 summer and 77 winter nasopharynx). From these samples the V4 region of the 16S rRNA gene was amplified and sequenced using the Illumina HiSeq2000 (llumina, San Diego, USA) under a single end 102 base-pair protocol.

Following de-multiplexing, reads were processed using the QIIME 1.9.1 toolkit, and only reads with an expected barcode, zero ambiguous base calls, less than three consecutive low-quality base calls, and a minimum Phred quality score of 20 along the entire read were kept. The reads that passed the QC filters were then taxonomically classified using the 97% identity Greengenes May 2013 reference database [12] and the Karp, Karp-Collapse, Kallisto and UCLUST algorithms. The UCLUST classification was carried out with classifier version 1.2.22q as part of the original study, the other classifications were performed for this study.

The Hutterite samples were previously genotyped using Affymetrix arrays (Affymetrix, Santa Clara, USA) [56–58]. Imputation was performed using the pedigree based approach PRIMAL [59] with a sequenced reference panel of 98 Hutterites. 3,161,460 variant sites with genotype call rates > 95% and a minor allele frequencies > 0.10 in any of the 4 season/site subsamples were then tested for association with the relative abundance of genera level taxa. Association was tested using a linear mixed model implemented in GEMMA [60]. The model included sex and age as fixed effects and a kinship random effect to adjust for the relatedness between study members. For each season/site combination, any genera that was classified as present in > 75% of samples by Karp, UCLUST, or Kallisto was analyzed. In total, 369 genera-season association analysis were performed (120 summer nasopharynx, 69 winter nasopharynx, 98 summer vestibule, 82 winter vestibule). To calculate q-values for our association statistics a subset of 148,653 SNPs pruned to have LD *r*^2^ < 0.5 were used as independent markers in each analysis and the Benjamini and Hochberg procedure [61] was applied.

### Implementation

The program Karp is implemented in C++, and available for download from GitHub at https://github.com/mreppell/Karp. Karp takes as input sample fastq files, reference sequences in fasta format, and taxonomy files with labels corresponding to the references. The first stage of analysis with Karp is building a k-mer index of the references. Karp then uses this index along with the reference sequences to pseudoalign, locally align, and then quantify the taxonomy in the fastq file of query reads. Karp includes a post analysis option to tabulate multiple samples and calculate compositional summaries. Karp can make use of multi-threading to improve performance, and allows users to specify frequency thresholds, EM convergence conditions, likelihood filter parameters, and pseudoalignment k-mer length.

The simreads program we used to simulate sequence data with an empirical distribution of base-quality scores is available at https://bitbucket.org/dkessner/harp.

## Results

### Comparison of competing methods with simulations

To test the performance of Karp against alternatives we simulated independent samples with 75bp single-end reads drawn from 1,000 reference sequences selected at random using two reference databases, one with full 16S gene sequences, and the other with only the V4 hypervariable region of the gene. We simulated samples at two depths: 100,000 reads per sample and 1,000,000 reads per sample. Each simulation used a unique set of 1,000 references, and the frequencies of each reference was varied to create a range of Shannon Diversity (S4 Fig). With both the full gene sequence and the V4 samples we classified sequences against the full 16S gene sequence in a GreenGenes database using Karp, Kallisto (v0.42.4), SINTAX (USEARCH v10.0.240) [62], the Wang *et al.* (2007) Naive Bayes classifier implemented in Mothur (v1.36.1) [63], the downloaded version of the 16S Classifier [41], and several algorithms from QIIME including UCLUST (v1.2.22q), USEARCH (v6.1.544), and SortMeRNA (v2.0). We estimated errors as described in section Simulations. Quantitative results from these simulations, and those with 151bp and 301bp paired-end reads are available in S4 File. If not explicitly stated, in the following paragraphs samples were 75bp single-end with 1,000,000 reads.

**Fig 2:**
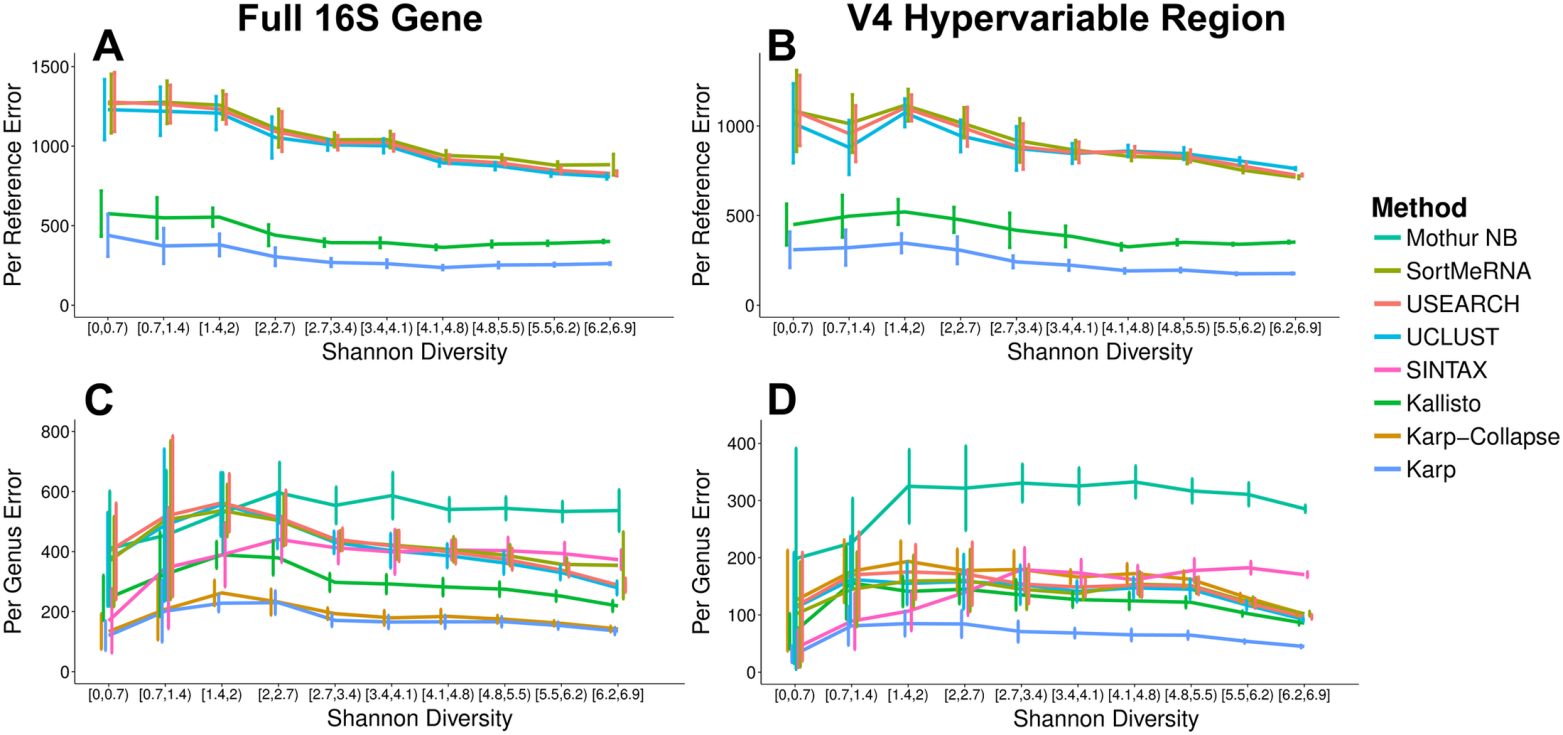
75bp simulation results. The average absolute error with 95% confidence intervals from simulated samples of 1 × 10^6^ 75bp reads, with every simulated dataset having a unique mix of 1,000 reference sequences. Each colored line represents a different quantification method, including Karp, Kallisto, SINTAX, UCLUST, USEARCH, SortMeRNA, and the Naive Bayes method implemented in Mothur. The samples were also classified with the 16S Classifier, its errors were above the shown range. Error refers to the average relative error (AVGRE): the difference between the true number of reads for each reference sequence present in the simulated data and the number classified by each method, if each method had classified every read in the data. (A) Per reference level error for taxa with frequency >0.1% in samples simulated from the full 16S gene sequence. (B) Per reference level error for taxa with frequency >0.1% in samples simulated using only the V4 hypervariable region of the 16S gene. (C) Genera-level error in the full gene samples. (D) Genera-level error in V4 samples.

We looked first at performance in 75bp single-end reads, identifying the exact reference sequence that was used to generate the sequencing reads in a sample. Across both sample sizes, at both samples depths, and in both the full gene and just the V4 region samples, the Karp algorithm had the lowest average errors (Fig 2). For example, in the full gene samples with 1,000,000 reads, Karp’s average error was 34% smaller than Kallisto and 65-66% smaller than UCLUST, USEARCH, and SortMeRNA. Across all the methods there was a trend of decreasing average error with increasing sample diversity. In the samples with 1,000,000 reads, Karp’s average error was 48% smaller when diversity was >6.2 than when it was <0.7.

Many reference sequences share the same taxonomic label, and researchers are often interested in hypotheses at a broader taxonomic level, like genera, than individual references. We aggregated counts for references with identical labels and again compared with the truth in our simulated samples. In the full gene high depth samples, the Karp and Karp-Collapse algorithms were the most accurate across diversity values and across taxonomic levels. (Fig 2C, S4 File). In the low depth and V4 samples, the Karp algorithm was still the most accurate, however Karp-Collapse’s performance was comparable to, or slightly worse than Kallisto and the QIIME algorithms. The SINTAX algorithm had accuracy similar to Karp in the lowest diversity samples (Shannon Diversity < 1.4), but decreased in accuracy as sample diversity increased. Across all evaluations Mothur’s Naive Bayes classifier and the 16S Classifier gave the least accurate estimates on average (Fig 2D).

The difference in quantification error observed here is relevant for downstream analysis. Using the full 16S gene we calculated summary statistics in 100 independent simulated samples. Karp’s estimates were on average closer to the truth than either Kallisto or UCLUST (Table 2). For Simpson Diversity, Karp’s estimate was within 10% of the actual value for 44% of samples, compared with 32% of samples for Kallisto, and only 2% of samples with UCLUST. Karp’s estimate of Simpson Diversity fell within 25% of the actual value in 82% of samples, with Kallisto this figure was 62%, and UCLUST was 18%.

**Table 2:** Summaries of microbiome data were calculated from 100 simulated samples containing different mixtures of 1,000 references. Only reference sequences with frequencies >0.1% were used to calculate the statistics. In each sample the absolute value of the difference between the actual statistic and that estimated by Karp, Kallisto, and UCLUST was calculated. The group-wise Beta Diversity value was a single estimate from all 100 samples; it is not an average and therefore there is no standard error.

	Individual	Pairwise		Group
Statistic	Simpson Diversity	^2^*D* Beta Diversity	Bray-Curtis Dissimilarity	^2^*D* Beta Diversity
Actual Values	0.002—0.9	0.44—0.87	0.40—1.0	0.025
	Average Absolute Difference (Standard Error)			
Karp	0.014 (0.029)	0.029 (0.035)	0.009 (0.018)	0.002
Kallisto	0.015 (0.025)	0.037 (0.046)	0.012 (0.023)	0.002
UCLUST	0.079 (0.13)	0.093 (0.090)	0.026 (0.043)	0.008

**Fig 3:**
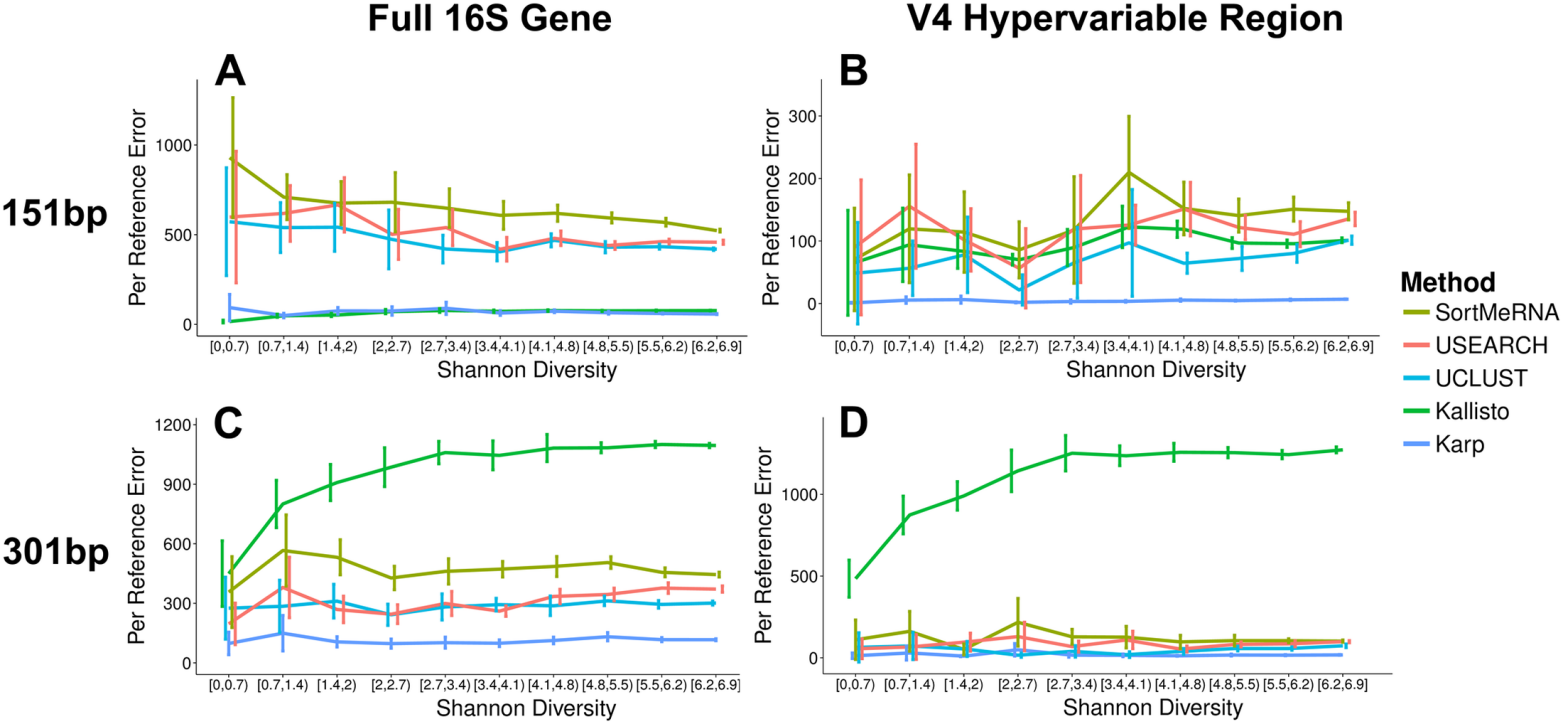
151bp and 301bp simulation results. Average relative errror (AVGRE) with 95% confidence intervals from the taxonomic quantification of simulated samples with 151bp paired-end and 301bp paired-end reads. Per reference error was calculated using the classifications from Karp, Kallisto, UCLUST, USEARCH, and SortMeRNA Only references with frequencies >0.1% in either the true reference or a simulated sample were used to calculate error. (A) Per reference error in full 16S gene samples of 151bp paired-end reads. (B) Per reference level error in 151bp paired-end samples simulated using only the V4 hypervariable region of the 16S gene. (C) Per reference error in full 16S gene samples of 301bp paired-end reads. (D) Per reference error in V4 samples of 301bp paired-end reads.

In addition to 75bp single-end reads, we simulated and classified samples with 151bp and 301bp paired-end reads, again at depths of 100,000 and 1,000,000 reads per sample, and using both the full 16S gene and just the V4 hypervariable region (Fig 3). With 151bp paired-end reads, in the full gene simulations at both depths, Kallisto had the lowest per reference error rate, with Karp a close second. On average Kallisto’s errors were 11% smaller than Karp’s at high depth and 19% smaller at low depth, and between 88-91% smaller than the QIIME algorithms across all comparisons. In the V4 simulations, at both depths, Karp had a substantially lower per reference error rate than the other methods: between 92% and 96% smaller across all comparisons. When we aggregated counts for references with identical taxonomic labels and compared their abundance estimates with the true values we saw different patterns in the V4 and full gene samples. In the V4 samples Karp and Karp-Collapse were very comparable with each other and had lower average errors than all other methods in almost all comparisons below the taxonomic level of class. SINTAX had the lowest errors in some low diversity bins at the taxonomic levels of family and order, and the lowest errors across the full diversity spectrum at the level of class. In the full gene simulations, Karp had the lowest average errors at taxonomic levels below class, with SINTAX the most accurate at class level classification. The second most accurate algorithm varied with taxonomic level: Kallisto at species, Karp-Collapse at genus and family, UCLUST at order, and Karp at class.

For the 301bp paired-end reads Kallisto’s strict pseudoalignment threshold struggled to make assignments. In the deeper samples, it classified only 3.8% of reads on average (versus 53% with Karp). Note that Kallisto’s performance could be improved by subsampling shorter regions from the longer reads, although this would be removing information that Karp is currently using to assign reads. Also, the Naive Bayes quantification could not be computed for the higher depth 301bp paired-end reads for the full gene samples with the computational resources available and was therefore not compared. In the 301bp samples, at the level of correctly identifying reference sequences, Karp was the most accurate, with average errors <90% smaller than Kallisto and <60% smaller than the QIIME algorithms across both depths in both the full gene and V4 samples. When we looked at errors in estimating taxonomic groups in the 301bp samples we see a more complex pattern (S4 File). In the full gene samples, at both depths, Karp is the most accurate for estimating species and genus frequencies (Karp average error at least 12% smaller than Karp-Collapse and at least 46% smaller than all other methods across comparisons). At higher taxonomic classifications both Karp algorithms, SINTAX, and the three QIIME algorithms perform well, and different ones have the lowest error in different comparisons: across family, order, and class and both depths UCLUST has the lowest error in four of the comparisons, Karp-Collapse in one, and SINTAX in one. For the V4 samples, Karp-Collapse had the lowest average errors for estimating species, genus, and family frequencies, while SINTAX and UCLUST performed the best at classifying orders and classes. Relative to the other methods, the Karp algorithms performed best in higher diversity samples and more specific taxonomic labels. Overall, in the V4 samples with the 301bp paired-end reads the errors for both Karp algorithms, SINTAX, and the QIIME algorithms were generally very small.

**Fig 4:**
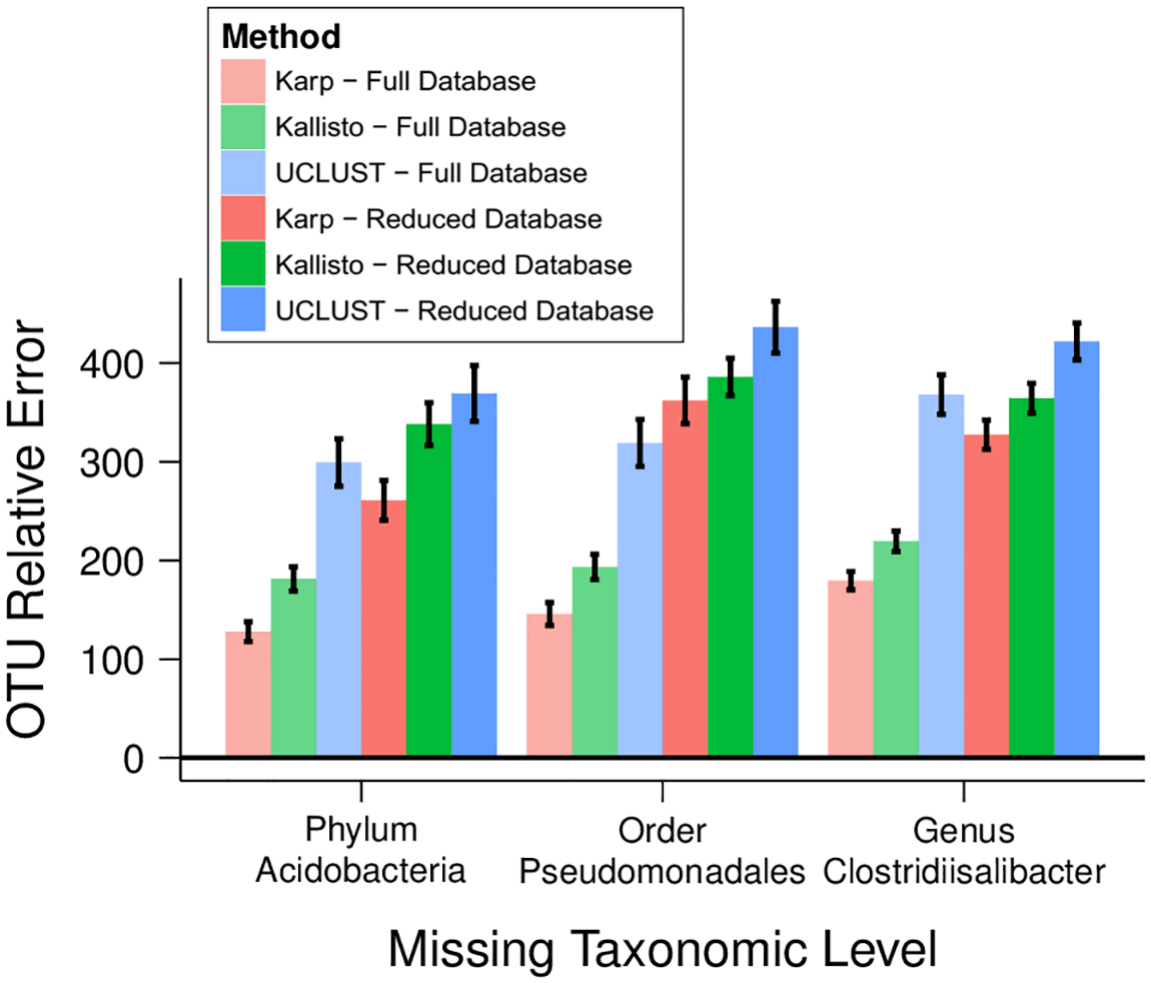
Results with missing taxa. Accuracy when the reference database used for quantification is missing taxa found in the sample. For each of one phylum (*Acidobacteria*), one order (*Pseudomonadales*), and one genus (*Clostridiisalibacter*), 10 samples were simulated where 50% of the reads originated from the noted taxa. Each sample was classified with the full GreenGenes database and also a reduced version of the database lacking all members of the taxa which had been used to simulate the sample. The accuracy of estimates by Karp, Kallisto, and UCLUST for the 50% of the samples that did not originate from the absent taxa were compared with their true frequencies. Black bars give 95% confidence intervals.

In microbiome quantification problems it is not uncommon to have taxa present in sequenced samples that are absent from reference databases. We tested the robustness of Karp under this scenario with simulated samples containing reads from references removed from the reference databases used for quantification. For each of one phylum (*Acidobacteria*), one order (*Pseudomonadales*), and one genus (*Clostridiisalibacter*) we simulated 10 independent samples where 50% of reads originated from 3 different members of each taxon and created copies of the GreenGenes database where the reference sequences for every member was removed. We classified the simulated data with both the reduced databases and the full GreenGenes to measure how much the absence of relevant references impacted estimate accuracy. Karp, Kallisto, and UCLUST were all less accurate when classifying samples using the reduced databases rather than the full database (Fig 4). Under all scenarios Karp remained the most accurate method, and in the case of the phylum *Acidobacteria* and genus *Clostridiisalibacter* Karp’s quantification using the reduced reference database was more accurate than UCLUST using the full reference database.

The model that underpins Karp relies on knowing the probability of a sequencing error at a given position in a read. Our work assumes that the base-quality scores are accurate estimates for the probability of sequencing error. In real data it has been recognized that base-quality scores are not always accurate, leading to the development of methods to empirically recalibrate base-quality using known monomorphic sites. With pooled microbiome samples this recalibration is complicated (possibly requiring spike-ins of known sequence during the experiment or alignment to conserved reference sequence), so we explored how differences between the expected sequencing error rate as represented by the base-quality scores and the actual sequencing error rate effect Karp’s accuracy. Under a model where the actual probability of a sequencing error was $10^{\frac{Q}{-5}}$ for quality score *Q*, rather than the $10^{\frac{Q}{-10}}$ assumed by our model, Karp was still on average more accurate than Kallisto or UCLUST/USEARCH (errors 12.9% smaller than Kallisto and 64.2% smaller than UCLUST, S5 Fig). When errors actually occurred uniformly at 1% of bases, grossly violating Karp’s model, it was still the most accurate method (errors 11.5% smaller than Kallisto, 62.8% smaller than UCLUST, S5 Fig).

The increased accuracy of Karp comes at some computational cost, especially relative to Kallisto, however it is still quite feasible for modern data. Table 3 compares the performance of the methods while classifying samples with either 10^6^ 75bp single-end, 151bp paired-end, or 301bp paired-end reads using 12 cores, and in all cases even the full mode of Karp requires <3 hours. Karp was run with default settings, in both full and collapse mode. For Karp and Kallisto the 75bp reads require longer to classify than the 151bp reads due to the larger number of multiply-mapped reads resulting from the shorter length.

**Table 3:** Computational requirements and speed of Karp, Kallisto, SINTAX, UCLUST, USEARCH61, SortMeRNA, and the Wang *et al.* (2007) Naive Bayes using Mothur. All programs were run using 12 multi-threaded cores except Mothur. Mothur’s memory requirements scale with the number of cores used, and in order to keep memory <16GB we limited it to 4 cores. The values for UCLUST and USEARCH give the time to assign taxonomy, generally with these methods reads are clustered before taxonomy is assigned and the value in parenthesis gives the time to first cluster and then assign taxonomy. The results for the method 16S Classifier are not shown: it was fast and required a few minutes at most; however its memory usage scaled dramatically with the number of reads. To keep memory usage < 128GB samples needed to be split into several smaller samples and then reassembled. Additionally, it could not be run in parallel, so a meaningful comparison against the other methods of speed and memory requirements was not possible.

Method	Time (Minutes)			Max Memory
	75bp	151bp	301bp	
Karp	161.9	24.7	80.4	10 GB
Karp-Collapse	36.0	16.1	74.5	10 GB
Kallisto	4.5	2.6	1.3	10 GB
SINTAX	267.9	361.8	366.5	2 GB
UCLUST	87.4 (146.6)	18.9 (67.1)	3.9 (55.9)	4 GB
USEARCH61	9.6 (27.7)	1.4 (10.0)	0.7 (7.1)	4 GB
SortMeRNA	37.3	22.3	29.6	4 GB
Mothur Naive Bayes*	502.4	1578.2	NA	16 GB

*limited to 4 cores

### Performance assessment in real 16S rRNA data

We next sought to evaluate the relative performance of the method in the context of real 16S data, where the true composition of samples is unknown. As a testbed, we re-analyzed data from a recent large-scale human nasal microbiome study [42] that aimed to detect associations between the relative frequencies of genera level taxa and host genotypes. For this study, genome-wide association studies (GWAS) are carried out where each genus is tested for association with each individual genotyped variant present in the genomes of the host individuals. Because lower rates of measurement error should allow for the discovery of more associations, we evaluated classification methods on the number of discovered associations, controlling for false discovery rate (FDR). Specifically, we classified 332 16S samples sequenced from 144 Hutterite individuals. The samples were brushed from two nasal locations (nasopharynx and vestibule) during two seasons (summer and winter). We classified the samples using the Karp algorithm, the Karp-Collapse algorithm, and Kallisto. We also used previous classification data created using the UCLUST algorithm [42]. With all four classification methods we tested for additive effects in the relative abundances of 369 genera level taxa from 3,161,460 SNPs using a linear mixed model that controlled for relatedness between the Hutterite individuals. Following the approach of Igartua et al. (2017) we used 148,653 SNPs with linkage disequilibrium *r*^2^ < 0.5 as a set of independent tests to calculate the false discovery rate (FDR). For this study we classified associations as independent if they involved different seasons, different genera, or had LD *r*^2^ < 0.5 and were >10kb apart.

The summer samples were more taxonomically diverse than the winter samples, and this was reflected in the number of associations detected, particularly in the nasopharynx. Across the four classification methods, at an FDR of 0.05, associations with 63 distinct genera were detected. Across the two tissues and two seasons, at an FDR rate of 0.05, the Karp-Collapse method detected 53 associations with 32 distinct genera (27 summer nasopharynx (SN), 8 summer vestibule (SV), 10 winter nasopharynx (WN), 8 winter vestibule (WV)), the Karp method 40 associations with 26 genera (17 SN, 9 SV, 10 WN, 4 WV), Kallisto 38 associations with 27 genera (23 SN, 4 SV, 5 WN, 6 WV), and the UCLUST algorithm 42 associations with 18 genera (15 SN, 11 SV, 2 WN, 6 WV) (S7 Fig). The relative ordering of methods, from most discoveries to least, was not dependent on the qvalue cutoff chosen, at a 0.1 threshold (131 Karp-Collapse, 121 Karp, 95 Kallisto, 120 UCLUST) and 0.01 threshold (12 Karp-Collapse, 4 Karp, 9 Kallisto, 8 UCLUST) the Karp-Collapse method also detected the most total associations. At an FDR threshold of 0.05, if we classify a signal as overlapping between methods when it is for the same genus, same season, and for SNPs with *r*^2^ > 0.5 within 10kb, the overlap between the Karp algorithms was 0.12 (10/83), it varied between 0.08 and 0.11 for either Karp algorithm and Kallisto or UCLUST. Kallisto and UCLUST only shared 0.04 (3/77) of their total associations.

## Discussion

In both simulations and real 16S data we have shown that Karp is an accurate and computationally feasible method for estimating the relative frequencies of contributing members in a pooled DNA sample. Although not as fast as some alternatives, Karp’s superior accuracy across the tested range of read lengths, taxonomic levels, and absent references makes a strong case for its adoption.

Although our work here has focused on applying Karp in the context of 16S microbiome experiments, its potential uses extend to most closed-reference quantification problems. Pooled DNA experiments are common in many fields. The identification and estimation of contributor abundance in whole genome metagenomics, pooled sequencing of data from artificial selection experiments, and RNA isoform identification are all possible with Karp. The limiting factor with extending Karp is not related to data type, but the diversity of kmers in the potential reference databases. The memory requirements of creating the the pseudoalignment kmer index is the limiting step, and is shared equally by Karp and Kallisto. For such applications, finding a suitable reference may be challenging, but the resulting improvements in accuracy from the application of Karp should be available.

A important consideration when designing a 16S sequencing experiment is how much of the gene to sequence, and what reference database to use for profiling. It has previously often been preferred to sequence either one or few hypervariable regions, in part to prevent clustering algorithms like those in QIIME from creating multiple taxonomic groups from the same reference at different regions. With Karp and Kallisto this concern can be ignored, opening up the possibility of capturing more fine scale information about the organisms present in a sample by sequencing a longer portion of their genome. In our V4 simulations we used a reference database that had been pared down to non-redundant reference sequences, so that we could definitively assess whether the estimate frequencies were accurate. This is not necessarily possible or desirable in a real experiment, and the cost of that needs to be understood. The shorter the reference used the more likely there are closely related organisms with identical reference sequences. Many more taxa have identical sequences across a single hypervariable region than across the entire 16S gene. Under such conditions the difference between methods that probabilistically assign reads to references, like Karp and Kallisto, and those that make a hard assignment, like UCLUST or USEARCH, can arise. With Karp, when references are nearly identical they will receive nearly equal probability weights from each read that maps to them, and the result will be many closely related references at low frequencies. With UCLUST or other similarity score methods, the references are sorted and the first of the closely related references to appear in the sorting order will be assigned all or nearly all the references, regardless of if it is the actual contributing organism. The truth in this case, is that the sequencing data does not contain enough information to accurately distinguish between the references, and both methods end up at sub-optimal, albeit different solutions. Under such conditions researches need to have a realistic expectation of what they can resolve in their data, and it is likely that inferences of higher-level taxonomic abundances rather than individual references are more likely to be robust. In such situations the Karp-Collapse algorithm is likely to perform well, as was the case with the real data association testing performed for this study. Now that the computational methods exist to work with multiple hypervariable regions or even the entire gene, we would suggest that experimenters could benefit substantially from sequencing more than is often presently used.

In addition to k-mer length, Karp users can adjust the thresholds for minimum frequency during the update step of the EM algorithm and the likelihood filter z-score. While results are often relatively invariant across a broad range of threshold values (S1, S2 and S3 Figs), avoiding extreme threshold values can improve quantification accuracy substantially. Practical guidance for setting the thresholds is given in Supplementary file S3 S3 File. It is worth noting that Karp’s tuning parameters influence performance as well as accuracy, so choosing optimum values can improve not just accuracy but experimental run time as well.

In both ecology and human health a greater understanding of the microbiome promises medical and scientific breakthroughs. Modern sequencing technology gives us unprecedented access to these microbial communities, but only if we can correctly interpret the pooled DNA that sequencing generates can we hope to make significant progress. Towards that end, Karp provides a novel combination of speed and accuracy that makes it uniquely suited for scientists seeking to make the most out of their samples.

## Supporting information

S1 File EM algorithm details. A more detailed description of the EM algorithm used by Karp.
(PDF)

S2 File Likelihood filter details. A more detailed description of the maximum likelihood z-score filter used by Karp.
(PDF)

S3 File Effect of Karp tuning parameters on run-time and accuracy. Discussion of the impact of Karp’s tuning parameters on the accuracy of results and the speed of computation, related to S1, S2 and S3 Figs.
(PDF)

S4 File Full simulation results table. Mean and standard deviation AVGRE values for all permutations of read length and sample depth for both the full 16S gene and V4 hypervariable region simulations. Includes the per reference error values as well as the results when estimates were collapsed to species, genus, family, order, or class classification.
(PDF)

S1 Fig K-mer length and Karp performance. Impact of k-mer length on Karp performance. Pseudoalignment indexes constructed using different k-mer lengths were used to classify 30 previously analyzed samples selected to cover a full range of Shannon Diversities. For each of 75bp, 151bp, and 301bp reads 10 samples of 1,000,000 reads were analyzed. (A) The average error values with 95% confidence intervals for each read length. (B) Average run times using 12-cores in parallel.
(PDF)

S2 Fig Impact of EM update minimum frequency threshold on program performance. Karp uses an EM algorithm to estimate the relative frequencies of reference sequences in a pooled sample. During the EM process a minimum frequency threshold can be applied that removes references with frequencies below this threshold. Set at a low frequency, the threshold helps remove spurious findings and improves accuracy, particularly for shorter reads. At higher frequencies the threshold removes references actually present in the sample and lowers accuracy. In this figure different thresholds are applied during quantification of 30 previously analyzed samples selected to cover a full range of Shannon Diversities. K-mers of length 19 were used for these analyses. For lengths of 75bp, 151bp, and 301bp 10 samples were analyzed. (A) The average error values with 95% confidence intervals for each read length. (B) Average run times using 12-cores in parallel. For shorter reads, increasing the threshold reduces the number of EM iterations required to converge and decreases run-time.
(PDF)

S3 Fig Impact of the EM update minimum frequency threshold on performance in common references. The impact of the EM frequency threshold is smaller when analyzing error in the estimates of more common references. Solid lines present the error calculated using all references classified, dashed lines give the error when only references with an actual or estimated frequency above >0.1%, a cut-off used frequently in this study. In such cases the chosen frequency threshold is less important.
(PDF)

S4 Fig Properties of simulated 75bp samples. Each simulated dataset contains reads from a mixture of 1,000 reference sequences (each an operational taxonomic unit: OTU). The frequencies at which reads were generated from contributing references were varied to create datasets with a range of Shannon Diversity. As diversity increases the frequency distribution begins to approach a uniform distribution.
(PDF)

S5 Fig Robustness of assumption that base quality scores reflect rate of sequencing error. Impact of assumption that base-quality scores accurately represent probability of sequencing error. For two different models of sequencing error we simulated 50 samples and classified them with Karp, Kallisto, and UCLUST/USEARCH. Each method is represented by a different colored line, and bars represent 95% confidence intervals (A) In our first model the true rate of sequencing error varied with the base-quality score, but was smaller than Karp’s model assumes. (B) In our second model, errors were distributed uniformly at 1% of bases in each read, independent of whatever base-quality score was assigned.
(PDF)

S6 Fig Accuracy of methods in simulated data when classifying 75bp samples at higher taxonomic levels. Average absolute error and 95% confidence intervals from the taxonomic quantification of 110 simulated samples, each comprised of 1,000,000 75bp paired-end reads. Taxonomy was classified using Karp, Kallisto, SINTAX, UCLUST, USEARCH, SortMeRNA, and the Naive Bayes implemented in Mothur. Counts were aggregated for OTUs classified in the same (A) family, (B) order, or (C) class and taxa with a frequency >0.1% were compared to their true counts.
(PDF)

S7 Fig Genomic association results with genera level taxa in real 16S sequencing data. Associations between the relative abundances of 369 genera level taxa in 332 16S sequencing samples and their host genomes. The 16S samples were drawn from brushings of two tissues, the nasopharynx and nasal vestibule, during either the summer or the winter. 3,161,460 SNPs were tested for association with the relative genera abundances estimated by four methods: Karp, Karp-Collapse, Kallisto, and UCLUST. There was greater microbiome diversity in the summer samples, particularly those from the nasopharynx, and this was reflected in a greater number of associations across all classification methods. At FDR thresholds of 0.01, 0.05, and 0.1 the Karp-Collapse method detected the most independent associations, and with the most distinct genera. Additionally, the Karp algorithms and Kallisto also shared a larger group of common associations with each other than they did with UCLUST.
(PDF)
