# Supplementary material for: Using pseudoalignment and base quality to accurately quantify microbial community composition
Source: PLoS Comput Biol. 2018 Apr 16;14(4):e1006096. doi: 10.1371/journal.pcbi.1006096 (PMC5945057; doi:10.1371/journal.pcbi.1006096)
Supplement: S1 File — A more detailed description of the EM algorithm used by Karp. (PDF) [file pcbi.1006096.s001.pdf]

EM algorithm details

For a pooled sample of reads r with $r \in 1, ..., N$, if we observed which reference haplotypes the reads in our sample originated from, η, and we assumed that conditional on the frequencies $\mathcal{F}$ the query reads are independent, it would be possible to calculate the maximum likelihood estimate $\hat{\mathcal{F}}$ using the complete data likelihood, which has the form

$$\mathcal{L}(\mathcal{F}|\eta, r) = P(\eta, r|\mathcal{F}) = \prod_{j=1}^{N} P(r_j, \eta_j|\mathcal{F}) \propto \prod_{k=1}^{M} f_k^{\sum_{j=1}^{N} \eta_{j,k}} \tag{1}$$

In actuality, we observe the reads but the reference haplotypes that they originate from are unobserved. To estimate $\mathcal{F}$ we therefore employ an EM algorithm. Briefly, the E-step of our procedure can be written

$$\begin{aligned} Q(\mathcal{F}, \mathcal{F}^{(i)}) &= \mathbb{E}_{\eta|r,\mathcal{F}^{(i)}}\left[\prod_{j=1}^{N} P(r_j, \eta_j|\mathcal{F})\right] \\ &\propto \mathbb{E}_{\eta|r,\mathcal{F}^{(i)}}\left[\sum_{k=1}^{M}\sum_{j=1}^{N} \eta_{j,k} log(f_k^{(i)})\right] \\ &= \sum_{k=1}^{M}\sum_{j=1}^{N} \mathbb{E}_{\eta|r,\mathcal{F}^{(i)}}\left[\eta_{j,k}\right] log(f_k^{(i)}) \end{aligned} \tag{2}$$

where

$$\begin{aligned} \mathbb{E}_{\eta|r,\mathcal{F}^{(i)}}\left[\eta_{j,k}\right] = P(\eta_{j,k} = 1|r, \mathcal{F}^{(i)}) &= \frac{P(r_j|\eta_{j,k} = 1)P(\eta_{j,k} = 1|\mathcal{F}^{(i)})}{P(r_j|\mathcal{F}^{(i)})} \\ &= \frac{l_{j,k} f_k^{(i)}}{\sum_{m=1}^{M} l_{j,m} f_m^{(i)}} \end{aligned} \tag{3}$$

The M-step directly follows from the form of our likelihood, and the algorithm updates the estimates of $\mathcal{F}$ until convergence according to

$$\hat{f}_k^{(i+1)} = \frac{\sum_{j=1}^{N} \mathbb{E}_{\eta|r,\mathcal{F}^{(i)}}\left[\eta_{j,k}\right]}{N} = \frac{1}{N}\sum_{j=1}^{N}\left[\frac{l_{j,k} f_k^{(i)}}{\sum_{m=1}^{M} l_{j,m} f_m^{(i)}}\right]. \tag{4}$$
