# Supplementary material for: Using pseudoalignment and base quality to accurately quantify microbial community composition
Source: PLoS Comput Biol. 2018 Apr 16;14(4):e1006096. doi: 10.1371/journal.pcbi.1006096 (PMC5945057; doi:10.1371/journal.pcbi.1006096)
Supplement: S2 File — A more detailed description of the maximum likelihood z-score filter used by Karp. (PDF) [file pcbi.1006096.s002.pdf]

Likelihood filter details

When we classify pooled microbiome data it is likely that some reads originate from taxa that are absent from our reference database. Filtering these reads improves the accuracy of frequency estimates for the taxa that are present. Karp uses a likelihood based filter that was first published and validated in Kessner *et al.* (2013).

Given a set of query reads with their corresponding base-quality scores, we can calculate the mean and variance for the distribution of likelihood values that would result if every query read were aligned to the actual reference that gave rise to it, such that every mismatch was the result of sequencing error. This calculation requires only the query read base-quality scores, not the actual reads or a reference database, and is carried out before Karp begins pseudoalignment.

Recalling the notation of section 'Read likelihoods', a read of length L, has bases $r_{[0]}, r_{[1]}, ..., r_{[L]}$ and corresponding base-quality scores $q_{[0]}, q_{[1]}, ..., q_{[L]}$. If each read r originated from a reference h, our goal is to calculate $\mathbb{E}\big[log\big(P(r|q,h)\big)\big]$ and $Var\big[log\big(P(r|q,h)\big)\big]$.

First, for each position $i \in 1, .., L$ define the empirical distribution of base-quality scores, $Q_{[i]}$, in a sample of N reads by

$$P(q_{[i]}|Q_{[i]}) = \frac{\sum_{j=1}^{N} I_{q_{j,[i]}=q_{[i]}}}{N} \tag{1}$$

where I is an indicator function and $q_{j,[i]}$ is the base-quality score at position i on read j. This distribution is independent of h.

Assuming that each position along a read is independent we can write:

$$\begin{aligned}
\mathbb{E}[log(P(r|q,h))] &= \mathbb{E}\Big[log\Big(\prod_{i=1}^{L} P(r_{[i]} \mid h_{[i]}, q_{[i]})\Big)\Big] \\
&= \sum_{i=1}^{L} \mathbb{E}\Big[log\Big(P(r_{[i]} \mid h_{[i]}, q_{[i]})\Big)\Big] \\
&= \sum_{i=1}^{L} \mathbb{E}\Big[\mathbb{E}\big[log\big(P(r_{[i]} \mid h_{[i]}, q_{[i]})\big) \mid q_{[i]}\big]\Big] \\
&= \sum_{i=1}^{L} \sum_{q_{[i]}} \Big[\mathbb{E}\big[log\big(P(r_{[i]} \mid h_{[i]}, q_{[i]})\big) \mid q_{[i]}\big] P(q_{[i]}|Q_{[i]})\Big]
\end{aligned} \tag{2}$$

For each position i the probability of sequencing error is a known function of the base-quality score, $\epsilon(q_{[i]})$. Karp assumes Phred scaled base-quality scores (with options for Phred+33 or Phred+64), where $\epsilon(q_{[i]}) = 10^{-\frac{q_{[i]}}{10}}$. Using $\epsilon(q_{[i]})$ and equation (2) in the main text we can write the conditional expectation as:

$$\begin{aligned} &\mathbb{E}\Big[log\big(P(r_{[i]} \mid h_{[i]}, q_{[i]})\big) \;\; \mid q_{[i]}\Big] = \\ &\qquad \big[1 - \epsilon(q_{[i]})\big] log\big(1 - \epsilon(q_{[i]})\big) + \epsilon(q_{[i]}) log\big(\epsilon(q_{[i]})/3\big) \end{aligned} \tag{3}$$

Note that this expression does not depend on $h_{[i]}$ or $r_{[i]}$. By combining equations 1, 2, and 3 we have an expression for $\mathbb{E}\big[log\big(P(r|q,h)\big)\big]$. To calculate $Var\big[log\big(P(r|q,h)\big)\big]$ we again use the assumption that bases are independent and write:

$$\begin{aligned} Var\big[log\big(P(r|q)\big)\big] &= \sum_{i=1}^{L} Var\big[log\big(P(r_{[i]}|q_{[i]})\big)\big] \\ &= \sum_{i=1}^{L} \Big[\mathbb{E}\big[log\big(P(r|q,h)\big)^2\big] - \mathbb{E}\big[log\big(P(r|q,h)\big)\big]^2\Big] \end{aligned} \tag{4}$$

The likelihood filter is applied after the query reads have been locally aligned to the reference database and the corresponding likelihood values have been determined. Then, a z-score is computed for each query read using its largest likelihood value and the mean and variance of the "null" likelihood distribution (Equations 3 and 4). If this z-score is too low it is evidence that the true reference that the read originated from is absent from the database, and correspondingly the read is removed.
