# Supplementary material for: Using pseudoalignment and base quality to accurately quantify microbial community composition
Source: PLoS Comput Biol. 2018 Apr 16;14(4):e1006096. doi: 10.1371/journal.pcbi.1006096 (PMC5945057; doi:10.1371/journal.pcbi.1006096)
Supplement: S3 File — Discussion of the impact of Karp’s tuning parameters on the accuracy of results and the speed of computation, related to S1, S2 and S3 Figs. (PDF) [file pcbi.1006096.s003.pdf]

Effect of Karp tuning parameters on run time and accuracy

While accuracy is largely similar across a range of values, understanding when adjusting the minimum EM update frequency or the z-score could improve results is important for Karp users. Setting the minimum EM frequency threshold too high causes the removal of real references present in the sample, while setting it too low can cause spurious references to be included in the final solution. In situations with enough information to distinguish between closely related references, for example if the entire 16S gene sequence has been sequenced, a greater frequency threshold can yield more accurate solutions (S2 Fig, S3 Fig). Under such conditions threshold values on the order of Karp's default (1/Number of reads) are often appropriate. Alternately, where only limited information exists, for example if a single hypervariable region has been sequenced, lower thresholds can give more optimal solutions. This is because a lower threshold avoids removing organisms truly in the sample that have had their reads spread across closely related taxa, each with a fraction of the true organism's frequency. With limited information setting the minimum frequency an order of magnitude lower than the Karp default (i.e. $1/(10 * \text{Number of reads})$) can yield better results.

For the z-score likelihood filter, Karp estimates the mean and standard deviation using the distribution of base-quality scores present in the data being classified. Thus, the quality of the data plays a role in determining the best threshold to use. Karp includes an option to output the distribution of maximum likelihood scores for a sample. Outputting these scores for a few samples, and comparing them with the z-score values output in Karp's log files is a good way to determine if the default threshold is too strict or lenient for a particular experiment. If the threshold is falling too near the median value of the real likelihoods, lowering it may improve accuracy by retaining more reads. If the threshold falls far outside the actual distribution of read likelihoods, setting it to a greater value could improve its ability to filter our reads from references absent from the reference database.

The speed of analysis is also impacted by tuning parameter choices (S1 Fig), k-mer length can affect the amount of reads that multiply map, which can have a large impact on analysis time. Likewise, more lenient frequency or likelihood filter thresholds can require longer to analyze simply due to the inclusion of more references and reads. If sufficient compute resources are available, prioritizing accurate estimation should be the priority, but researchers should be aware of the impact of their choices on analysis times.
