# Supplementary material for: Using pseudoalignment and base quality to accurately quantify microbial community composition
Source: PLoS Comput Biol. 2018 Apr 16;14(4):e1006096. doi: 10.1371/journal.pcbi.1006096 (PMC5945057; doi:10.1371/journal.pcbi.1006096)
Supplement: S4 File — Mean and standard deviation AVGRE values for all permutations of read length and sample depth for both the full 16S gene and V4 hypervariable region simulations. Includes the per reference error values as well as the results when estimates were collapsed to species, genus, family, order, or class classification. (PDF) [file pcbi.1006096.s004.pdf]

Simulation Results

February 17, 2018

Values given are average values of AVGRE (AVerage Relative Error) metric with standard deviations given in parenthesis:

$$AVGRE = \frac{1}{1000} \sum_{i \in M_a \cup M_e} \left| T_{i,e} * \frac{\sum_{i \in M_a} T_{i,a}}{\sum_{i \in M_a \cup M_e} T_{i,e}} - T_{i,a} \right| \tag{1}$$

Where M_a as the set of actual haploid reference sequences contributing to a pooled sample and M_e as the set of additional references a method classifies as having a non-zero number of reads that are not truly present. Also, let $T_{i,e}$ be the count of reads estimated for reference i and $T_{i,a}$ is the actual number of simulated reads from reference i present in the sample.

Table 1: **Per reference error rates, 1,000,000 reads**

Read Length	Region	Samples	Method	Shannon Diversity									
				[0,0.7)	[0.7,1.4)	[1.4,2)	[2,2.7)	[2.7,3.4)	[3.4,4.1)	[4.1,4.8)	[4.8,5.5)	[5.5,6.2)	[6.2,6.9]
75bp SE	Full gene	110	Karp	439.3 (262.1)	372.3 (178.9)	379.3 (111.4)	304.4 (90.7)	268.4 (41.2)	260.8 (39)	236.2 (21.6)	252 (28.4)	254.7 (15.5)	261.5 (12.2)
			UCLUST	1229.1 (376.1)	1218.9 (246.3)	1207.1 (168.5)	1054.1 (209.2)	1008.2 (55.7)	1002.1 (72)	894.4 (33.7)	874.6 (38.5)	827.6 (29)	808 (30.8)
			SortMeRNA	1267.3 (367)	1275.8 (216.7)	1257.6 (144.9)	1112.7 (193.6)	1039.5 (72)	1041 (84.6)	942.5 (47.3)	928.3 (28.6)	880.5 (34.4)	883.2 (130.5)
			Kallisto	575.3 (283)	548.9 (206.1)	553.3 (93.5)	439.9 (106.7)	393 (38.3)	391.9 (49.7)	363.4 (23.7)	384.3 (31.2)	389.4 (21.6)	400.5 (15.5)
			USEARCH61	1276.9 (367.9)	1264.4 (191.7)	1231.9 (149.3)	1092 (202.6)	1021.1 (74)	1021.3 (85.1)	917.2 (46.4)	895 (29.3)	848.3 (33.4)	828.4 (31.7)
	V4	130	Karp	309.3 (227.7)	321.2 (224.5)	345.9 (121.2)	306.3 (121.6)	241.6 (54.5)	222.1 (45.4)	191.3 (19.8)	195.4 (16.4)	175.2 (8.3)	176.9 (5.4)
			UCLUST	1083.5 (517.4)	1013.1 (368.4)	1115.6 (203.2)	1018 (136.9)	919 (197.1)	867.5 (83.7)	831.1 (41.1)	818.8 (49.1)	755.9 (27.6)	713.9 (13.7)
			SortMeRNA	1014.8 (505.2)	880.1 (345.4)	1073.2 (179.1)	944.3 (143.4)	874.6 (197.3)	846.3 (90)	860 (52.4)	845.7 (54.1)	805.1 (29.5)	762 (10.6)
			Kallisto	449.8 (262.1)	496.3 (267.7)	520.4 (160.2)	476.2 (113.3)	417.9 (155.1)	383.6 (93.7)	325.5 (29.5)	350.6 (24.3)	339.8 (8.5)	352 (9.4)
			USEARCH61	1086.5 (447.6)	957 (357.6)	1100.7 (166.1)	995.5 (176.2)	885.4 (205.6)	852.6 (93.4)	855.9 (47.6)	832.2 (55.5)	778.3 (29.9)	726.1 (8.6)
151bp PE	Full gene	130	Karp	93.6 (118.9)	50.9 (35)	75.2 (43.8)	74.7 (37.7)	89.4 (55)	63.5 (20.8)	73.5 (12.2)	65.2 (13.1)	60.8 (4.7)	57.6 (3.8)
			UCLUST	572.3 (484.7)	552.4 (388.9)	541.7 (313.8)	473.8 (264.9)	419.9 (124.4)	405.3 (82.4)	468.6 (59.1)	430.3 (51.2)	433 (22.3)	419.2 (9.9)
			SortMeRNA	929.2 (540)	726.5 (343.7)	676.1 (281.3)	680.9 (266.3)	648.4 (173.2)	607.8 (125.3)	619.8 (73.1)	593.6 (46.1)	569.6 (35.4)	523 (11.1)
			Kallisto	15.9 (12.6)	46.8 (24.4)	52.6 (21.4)	71.3 (19.4)	77.6 (17.1)	72.8 (11.1)	76.7 (7.3)	75.3 (5.3)	76.5 (5.2)	77.1 (2.9)
			USEARCH61	598.1 (591.3)	630.4 (436.8)	666.3 (354.2)	502 (225.4)	539.5 (162.3)	419.1 (106.6)	480 (65.3)	439.8 (35.3)	461.7 (22)	458 (17.2)
	V4	190	Karp	1.2 (2.2)	5.5 (16.7)	6.2 (18.5)	1.9 (1.4)	3.2 (4.3)	3.5 (2.2)	5.5 (3.1)	4.7 (1.2)	6 (2)	6.9 (0.8)
			UCLUST	48.5 (262.1)	56.4 (140.8)	77.9 (194.9)	21.7 (39)	65.1 (91.1)	97 (137.6)	64.4 (25.9)	72 (30.9)	80.1 (22.7)	101.3 (10.8)
			SortMeRNA	70.6 (264.4)	119.5 (278.2)	114.3 (207.8)	85.8 (72.4)	117.7 (137.5)	209.8 (144.6)	152.1 (68)	140.7 (42.6)	150.9 (31)	147.5 (22.1)
			Kallisto	65.2 (270.8)	93.9 (189.5)	83.3 (87.4)	70.1 (14)	89.6 (46.8)	122.5 (53.5)	118.8 (21)	96.7 (14)	95.7 (9.6)	101.2 (6.2)
			USEARCH61	89.8 (348.2)	155.6 (320)	101.8 (161)	56.4 (102.4)	119.2 (138.1)	125.6 (51.3)	151.1 (69.3)	121.7 (31.2)	110.9 (32.3)	135.3 (17.4)
301bp PE	Full gene	170	Karp	97.7 (129.6)	149.4 (228.2)	105.7 (73.1)	97 (59.1)	101.7 (63.4)	98.8 (27.3)	112.7 (27.1)	131.7 (33.7)	117 (16.9)	116.6 (8.2)
			UCLUST	275.6 (356.4)	285.1 (334.8)	310.7 (237.5)	242.3 (137.1)	281.6 (150.3)	293.2 (51.4)	287.6 (79.4)	312.2 (38.5)	294.7 (31)	301.3 (16.2)
			SortMeRNA	356.6 (407.6)	566.2 (457.5)	531.9 (246.7)	427.6 (151.1)	460.8 (145.7)	471.9 (63.3)	485.6 (81.3)	505.2 (47.6)	456.1 (36.3)	444.8 (19.4)
			Kallisto	449.5 (376.7)	800.7 (304.1)	908.6 (253.7)	985.8 (249.8)	1059.5 (128.8)	1045.4 (117.6)	1082.2 (110.4)	1083.8 (41.3)	1100.3 (25.2)	1095.2 (17.5)
			USEARCH	196.1 (238.5)	380 (389.1)	269 (187)	245.3 (121.1)	299.2 (138.6)	260.7 (42.7)	334.5 (58.3)	344.5 (51.5)	376.7 (43)	371.7 (25.6)
	V4	190	Karp	12.6 (54.5)	29.5 (139.3)	10.1 (10.3)	48.8 (61.9)	16 (20)	14.9 (13.4)	12.2 (4.3)	16.8 (9.6)	15.9 (5.4)	17.6 (3.3)
			UCLUST	63.3 (288.8)	72.5 (269.5)	54.2 (148.8)	15.7 (20.4)	39.3 (56.8)	19.1 (28.5)	38.8 (28.7)	56.6 (24)	56.5 (16.5)	73.3 (18.1)
			SortMeRNA	110.4 (389.9)	161.8 (393.1)	51.1 (108.5)	217.1 (235.3)	128.4 (73.3)	126.4 (106.9)	97.4 (65.2)	105 (53.4)	104.8 (19.5)	101.9 (11.4)
			Kallisto	484.1 (366.5)	873.8 (372.9)	990.5 (281.5)	1143.9 (205.7)	1251.8 (174.9)	1236.2 (97.9)	1257.4 (89.1)	1255.3 (51.2)	1243.3 (41.6)	1272.6 (29.5)
			USEARCH61	54.9 (194.6)	66 (243.9)	95.6 (177.7)	129.8 (141)	68.4 (54.8)	108.9 (86.2)	53.4 (29.7)	81.6 (25.2)	85.4 (29.9)	98.6 (10.5)

SE=Single-end, PE=Paired-end

Table 2: **Per reference error rates, 100,000 reads**

Read Length	Region	Samples	Method	Shannon Diversity									
				[0,0.7)	[0.7,1.4)	[1.4,2)	[2,2.7)	[2.7,3.4)	[3.4,4.1)	[4.1,4.8)	[4.8,5.5)	[5.5,6.2)	[6.2,6.9]
75bp SE	Full gene	130	Karp	39.7 (21)	41.1 (14.3)	41.5 (15.2)	37.5 (8.3)	34.7 (6.8)	32.9 (2.9)	32.5 (1.7)	31.7 (1.7)	32.3 (1.4)	33.7 (0.8)
			UCLUST	105.9 (45.4)	118.1 (18.4)	113.2 (16.3)	108.4 (14.7)	102.6 (10)	98.8 (3.3)	92.9 (5.2)	86.7 (3.6)	82.8 (1.8)	78.1 (1.1)
			SortMeRNA	113.7 (41.4)	122.8 (18.1)	118.7 (16.6)	117.5 (12.2)	109.1 (6.4)	103.5 (4.7)	100 (3.9)	93.9 (1.5)	90 (1.7)	85.9 (1)
			Kallisto	55.2 (22.9)	53.8 (16.2)	53.2 (15.4)	48.1 (8.5)	44.8 (6.4)	42.8 (3.5)	42.6 (1.8)	41.4 (1.7)	42.5 (1.4)	44.1 (0.9)
			USEARCH61	111.2 (41.7)	123 (17.6)	117.4 (17.5)	113.9 (14.2)	105.4 (6.8)	98.7 (5.7)	94.9 (4.5)	88.7 (1.9)	84.2 (1.9)	79.5 (0.9)
	V4	130	Karp	34 (25.5)	31.9 (19.3)	34.2 (13.9)	37.5 (12.8)	29.9 (9)	24.9 (5.3)	23.8 (3.5)	22.5 (2)	22.6 (1.5)	23.2 (0.8)
			UCLUST	104.3 (49.1)	101.5 (43.4)	105 (25.1)	111.2 (27.1)	102.2 (13.9)	91.7 (11.8)	88 (6.1)	80.6 (4.8)	78.6 (3.5)	72.5 (1.7)
			SortMeRNA	114.3 (47.2)	110.5 (44)	106.2 (20.1)	110.2 (22.1)	98.9 (13.2)	91.3 (7)	89.6 (5.4)	88.3 (4.6)	84.7 (2.3)	80.7 (1.2)
			Kallisto	48.8 (28.8)	49.5 (27)	55.4 (26.6)	52.9 (13.9)	45.7 (10.3)	40.5 (10.9)	37.8 (4.6)	36.2 (3.5)	37.2 (2.4)	37.4 (1.2)
			USEARCH61	121.1 (48.4)	113.9 (42.4)	108.6 (21)	110.3 (22.5)	99.8 (13.5)	88.6 (7.3)	87 (6.1)	84.6 (5.4)	78.9 (2.3)	73.4 (1.4)
151bp PE	Full gene	130	Karp	7.3 (8.8)	7 (7.3)	9 (5.6)	7.4 (2.8)	6.5 (2)	6.4 (1.2)	7.1 (1.5)	7 (1.3)	6.4 (0.6)	5.9 (0.5)
			UCLUST	45.5 (40.8)	43.1 (31.5)	53.5 (30.9)	50.6 (23.2)	39.5 (7.7)	43.9 (8.6)	46.4 (5.9)	44.9 (4.8)	46.5 (3.1)	44.8 (2.7)
			SortMeRNA	65.1 (43.2)	45.4 (27.3)	75.1 (28.4)	73.2 (20)	54.9 (10.8)	65.4 (5.5)	63.1 (3.9)	63.5 (4.7)	63.1 (3)	56.4 (1.9)
			Kallisto	2.6 (2.4)	4.1 (2.2)	5.3 (2.4)	6.6 (1.6)	6.4 (1)	6.7 (1)	7.7 (0.6)	7.6 (0.5)	7.9 (0.4)	8.8 (0.2)
			USEARCH61	55.9 (50)	44.2 (34)	52.9 (23.9)	52.8 (20.3)	44.8 (12.4)	45.5 (5.2)	50.3 (4.4)	48.8 (5.7)	49.2 (2.6)	49.1 (2.6)
	V4	190	Karp	0.1 (0.1)	0.3 (0.5)	0.7 (2.2)	0.3 (0.1)	0.8 (0.5)	0.8 (0.9)	0.9 (0.4)	0.9 (0.2)	1.2 (0.2)	1.6 (0.1)
			UCLUST	5.1 (25.3)	4.8 (20)	7 (15.1)	11.5 (11.7)	8.1 (6.9)	8.5 (8.1)	10 (5.6)	7.2 (2.9)	8.2 (2.2)	10.5 (1.3)
			SortMeRNA	3.8 (12.4)	18.4 (33.2)	16.6 (27.3)	19.2 (20.2)	11.9 (8.3)	16.7 (10.4)	16.4 (7)	14.6 (4.1)	13.8 (3.1)	15.4 (1.5)
			Kallisto	2.3 (1.7)	5.5 (2.9)	8.3 (5.2)	11.1 (9.4)	10.1 (4.9)	9 (1.8)	10.4 (2.3)	10.8 (2.3)	10.1 (0.6)	11.5 (0.6)
			USEARCH61	5 (25.2)	12.4 (36.8)	8.4 (15.7)	18.5 (15.5)	9.9 (7.6)	9.5 (8.1)	14.9 (8.4)	14.7 (5.4)	11.3 (3.3)	14.2 (1.2)
301bp PE	Full gene	130	Karp	5.5 (7.7)	8.7 (11.5)	12 (11.4)	13.5 (10.4)	9 (3.1)	11.7 (4.8)	11.1 (1.7)	11.7 (1.5)	11.4 (1.5)	11.5 (0.6)
			UCLUST	18.7 (20.5)	25.4 (29)	37.2 (27.6)	34.2 (23.5)	23.6 (9.8)	32.4 (6.8)	31.6 (4.6)	32.2 (5.9)	33.2 (4.3)	31.5 (1.7)
			SortMeRNA	40 (35.3)	39.8 (30.7)	47.6 (22)	56.5 (20.9)	43.6 (12.8)	52.7 (12.4)	49.6 (6.1)	49.3 (4.9)	49.3 (3.8)	46.5 (2.1)
			Kallisto	43.1 (36.3)	68.6 (35.7)	89.6 (27.3)	89.9 (20.8)	98.8 (19.4)	100.5 (11.7)	110.9 (13)	106 (5.2)	111.6 (5.4)	113.1 (2.3)
			USEARCH61	29.1 (47.3)	27.5 (34.6)	32.4 (28.4)	35.5 (25.7)	26.4 (9)	34.3 (10.3)	31.6 (4.5)	32.3 (2.6)	32.6 (4)	32.7 (2.3)
	V4	190	Karp	0.6 (1.9)	0.9 (1)	1.5 (2.2)	1.8 (1.7)	2 (2.9)	1.7 (0.9)	1.9 (0.3)	2.5 (0.5)	2.9 (0.6)	3.9 (0.3)
			UCLUST	0.5 (1)	4 (20)	11.6 (23.1)	1.9 (2.7)	2.8 (3.6)	4.4 (4.1)	3.9 (2.3)	5.2 (2.7)	6.4 (2)	7.3 (1.1)
			SortMeRNA	5.2 (27)	5.3 (15.1)	11.5 (21.8)	7.9 (12.3)	9.4 (10.9)	15.7 (7.1)	10.2 (5.4)	10.5 (4)	10.9 (3.2)	10.6 (1.5)
			Kallisto	62.2 (48.3)	100.3 (38.1)	90.2 (27.9)	113.5 (14.4)	112.3 (17.9)	118.5 (10.5)	121.2 (11.8)	129.2 (6.5)	128.5 (3.7)	132.7 (2.2)
			USEARCH61	9.2 (39)	6.7 (21.3)	6.1 (14.8)	5.1 (7.2)	6.2 (9.1)	4 (2)	7 (2.5)	8.1 (3.6)	8.1 (1.8)	11.2 (1.3)

SE=Single-end, PE=Paired-end

Table 3: **Per species error rates, 1,000,000 reads**

Read Length	Region	Samples	Method	Shannon Diversity									
				[0,0.7)	[0.7,1.4)	[1.4,2)	[2,2.7)	[2.7,3.4)	[3.4,4.1)	[4.1,4.8)	[4.8,5.5)	[5.5,6.2)	[6.2,6.9]
75bp SE	Full gene	110	Karp	28.6 (41.4)	74.6 (67.4)	103.2 (72.5)	91.7 (37.2)	59.7 (21.4)	67.6 (32.8)	63.5 (19.4)	71.2 (23.4)	72.9 (17.7)	71.1 (13.7)
			Karp-Collapse	39.3 (62.1)	111.9 (102.9)	141.4 (68.6)	131.9 (88.9)	99.3 (30.6)	91.1 (29.8)	87.1 (15.3)	89.8 (20.2)	88.6 (14.1)	84.3 (12.1)
			UCLUST	90 (142.5)	265.3 (308.5)	281.6 (259.6)	267.7 (158.4)	210.6 (102.1)	181.2 (78.9)	176.2 (48.3)	176 (50.9)	178.3 (40.9)	170 (40)
			SortMeRNA	78.9 (120.8)	295.5 (368.3)	291.1 (240.6)	265.4 (168.6)	231.4 (109.6)	193.8 (76.6)	182.2 (52.5)	185.7 (48.1)	185.8 (38.5)	199.6 (105.4)
			Kallisto	49.2 (65.3)	119.5 (104.7)	157.6 (106.1)	141.5 (56.2)	96 (30.1)	113.2 (49.9)	103.2 (28.4)	113.9 (32.7)	117.7 (25.1)	111.1 (20.4)
			USEARCH61	82 (122)	297 (361)	297.6 (261.9)	274 (168.9)	230.2 (101.3)	203.5 (90.4)	181.8 (49.2)	187.9 (49.6)	185.3 (40.7)	176.8 (41)
			Mothur NB	102.1 (183.2)	302.5 (329.3)	326.5 (234.5)	351.1 (182.2)	256.4 (86)	288.3 (125.5)	243.9 (60.9)	251.9 (54.6)	243 (46.5)	282 (205.3)
			16S Classifier	204.9 (365.4)	289.7 (330.9)	299.6 (283.8)	383.8 (144.3)	306.5 (141.3)	319.4 (152.4)	297.2 (58)	297.6 (83.2)	305.7 (68.1)	296.5 (60.6)
			SINTAX	49.2 (79.6)	241.3 (312.3)	245.6 (227.6)	241.9 (187.7)	186.0 (116.8)	205.2 (103.4)	199.1 (41.0)	196.2 (58.2)	208.3 (51.5)	203.9 (40.7)
	V4	130	Karp	5.8 (8.2)	32 (53.2)	15.2 (20.5)	15.8 (15.1)	30.2 (22.6)	23.8 (6.8)	18.3 (6)	18.9 (6.5)	15.1 (4)	15.5 (2.3)
			Karp-Collapse	16.4 (18.6)	65.1 (80.9)	30.3 (37.4)	30.3 (24.1)	59.7 (38.8)	59 (13.1)	52.9 (12.1)	54.1 (13.1)	48.1 (5)	41.5 (3.5)
			UCLUST	11.4 (14.4)	70.9 (132.5)	23.1 (29.8)	32.5 (37.9)	68.5 (55.4)	53.6 (21.6)	44.9 (9.7)	47 (18.3)	44.8 (10.4)	41 (4.8)
			SortMeRNA	15.1 (22.9)	65.6 (114.8)	24.2 (31.7)	33.3 (35.7)	74.5 (66.5)	52.1 (13.4)	49.3 (20.2)	50.7 (19.6)	44.3 (8.3)	46.1 (4.3)
			Kallisto	16.2 (19.3)	64.1 (115.8)	20.1 (23.3)	22.9 (18.8)	57.4 (71.1)	38.1 (13.5)	31.1 (8.4)	33.4 (11.8)	30.5 (7.2)	30.9 (3.9)
			USEARCH61	18 (26.8)	78.4 (122.7)	25.6 (33.6)	38.5 (44.4)	77.1 (67.5)	52.5 (13.8)	47.2 (15)	50.4 (20.8)	45.1 (9.9)	44.3 (4.6)
			Mothur NB	13.6 (15.7)	96 (133.4)	55.6 (102.3)	70.8 (76.4)	111.5 (73.5)	94.9 (40.2)	93.4 (32.7)	97.8 (16.2)	98.3 (15.2)	96.8 (4.3)
			16S Classifier	28.1 (38.2)	171.7 (276.1)	51.8 (97.6)	68.4 (92.8)	108.1 (98.9)	68.6 (35.3)	66.9 (30.5)	69.5 (11.6)	78.5 (15.4)	82.8 (5.5)
			SINTAX	1.8 (<0.1)	31.3 (<0.1)	10.0 (<0.1)	32.5 (53.8)	65.8 (52.7)	43.9 (27.5)	40.4 (16.7)	46.3 (19.8)	48.0 (16.5)	49.9 (6.0)
151bp PE	Full gene	130	Karp	2.8 (4.5)	3.7 (6.8)	4.3 (8.4)	7 (8.1)	9.5 (7)	8.9 (5)	8.1 (3.3)	9.9 (2.7)	10 (1.5)	9.4 (0.8)
			Karp-Collapse	13.8 (18.8)	22.2 (40.4)	13.2 (20.9)	46.5 (53.5)	31.1 (25.8)	40.6 (20.5)	35.2 (15.8)	41 (14.8)	40.4 (9.5)	39 (3.6)
			UCLUST	15.2 (21.3)	33.8 (74.6)	43.6 (75.9)	35.6 (32.9)	42 (24.8)	48.6 (27.6)	54.7 (17.1)	55.2 (11.8)	59.7 (10.7)	59.3 (7)
			SortMeRNA	24 (32.9)	48 (86.3)	55.5 (73.2)	59.6 (28.3)	86.3 (45.4)	80 (40.7)	87.3 (33.8)	75 (18.9)	81.5 (13.1)	74.7 (3.6)
			Kallisto	1.7 (2.2)	7 (15.4)	13 (23.1)	11.6 (11.3)	13.8 (9)	16.7 (8)	21.5 (8.7)	17.4 (4.4)	19.9 (3.8)	19.1 (1.4)
			USEARCH61	17 (18.7)	56.4 (133.8)	69.4 (88.7)	39.7 (45)	59.1 (38.3)	52.4 (27.7)	56.2 (20)	55 (12.7)	67 (12.7)	67.3 (3.4)
			Mothur NB	48.4 (49.3)	108.1 (102.9)	113.8 (103.6)	120.6 (48.4)	161.1 (33.8)	175.2 (59.6)	180.5 (32.6)	168.6 (20.9)	162.7 (14.9)	140.9 (6.9)
			16S Classifier	213.6 (354.9)	247.2 (317.4)	292.2 (259.4)	213 (95.8)	273.5 (108.9)	270.8 (101.5)	276 (52)	240.6 (34)	267.9 (32)	254.9 (9)
			SINTAX	20.1 (28.9)	32.3 (64.6)	61.6 (81.9)	51.8 (49.6)	79.0 (32.0)	96.2 (52.9)	98.6 (37.4)	97.2 (22.3)	102.3 (19.0)	99.1 (6.2)
	V4	190	Karp	<0.1 (<0.1)	0.1 (0.5)	2.1 (11.3)	0.1 (0.2)	1.2 (3.3)	0.4 (0.7)	0.4 (0.8)	1.1 (1.2)	0.9 (0.7)	1.5 (0.7)
			Karp-Collapse	<0.1 (<0.1)	0.1 (0.2)	2.2 (11.4)	0.1 (0.3)	1.2 (3.4)	0.9 (1.6)	0.6 (0.9)	1.5 (1.7)	0.8 (0.7)	1.3 (0.9)
			UCLUST	1.1 (4.3)	0.2 (0.5)	0.4 (0.9)	15.7 (44.2)	15 (44.9)	6.5 (12.2)	3.6 (7.5)	3.6 (6.1)	5.7 (5.5)	7.5 (2.4)
			SortMeRNA	1.1 (3.9)	2.6 (7)	0.5 (1.3)	17.1 (45.9)	13 (26.8)	14 (25.1)	7.5 (9.7)	9.5 (11.4)	10.7 (8.8)	9.7 (4.7)
			Kallisto	0.5 (0.9)	29.5 (155.7)	2.1 (5.7)	1.5 (3.1)	12.7 (28.1)	24.5 (45.9)	8.7 (10.3)	6.6 (5)	6.9 (3.8)	7.3 (1.8)
			USEARCH61	0.8 (3.9)	67.8 (251.7)	1.9 (5.3)	0.2 (0.5)	17.2 (49.9)	15 (26.1)	12.3 (14.6)	7.8 (8.7)	9.6 (6.5)	8.4 (2.2)
			Mothur NB	61.7 (81)	54.8 (78.7)	89.9 (104.7)	112.2 (132.3)	99.3 (77.2)	132.6 (49.2)	102.8 (38.1)	94.4 (18.5)	99.2 (13.2)	103.9 (11.7)
			16S Classifier	165.2 (335.8)	133.2 (242.7)	123.2 (123.9)	82.5 (107)	67.1 (42)	126.7 (87.2)	79.2 (34.3)	69.4 (16.4)	73.2 (13.3)	81.8 (12.5)
			SINTAX	2.4 (5.2)	21.7 (60.5)	35.4 (86.0)	18.2 (45.5)	47.3 (61.9)	80.8 (100.7)	28.9 (24.6)	26.3 (16.5)	20.8 (10.3)	29.1 (6.6)

SE=Single-end, PE=Paired-end

Table 4: **Per species error rates, 1,000,000 reads**

Read Length	Region	Samples	Method	Shannon Diversity									
				[0,0.7)	[0.7,1.4)	[1.4,2)	[2,2.7)	[2.7,3.4)	[3.4,4.1)	[4.1,4.8)	[4.8,5.5)	[5.5,6.2)	[6.2,6.9]
301bp PE	Full gene	170	Karp	0.7 (1)	2.3 (2.5)	9.3 (19.9)	13.3 (20.7)	13.3 (11.7)	8.6 (6.5)	14.4 (4.7)	13.7 (3.2)	16.1 (4.4)	14.8 (2.9)
			Karp-Collapse	2.9 (5)	14.9 (27.6)	17.6 (41.2)	12.5 (18.6)	17.1 (18)	14.5 (7.3)	21.7 (12.5)	19 (5.7)	18.6 (5)	16.5 (2.4)
			UCLUST	6.3 (13.3)	16 (33)	23.5 (63.1)	26.6 (40.2)	54.7 (78.7)	34.5 (31.6)	37.3 (17.2)	39.9 (9.7)	43.8 (12.2)	42.3 (5.9)
			SortMeRNA	15.1 (31.7)	48 (85.1)	43.7 (47.4)	53.6 (54.9)	73.1 (73.7)	60.6 (31.6)	66.2 (24.3)	75.2 (14.3)	72.9 (12.9)	71.4 (10.1)
			Kallisto	27.8 (54.7)	112.9 (228.6)	60.8 (74.3)	194.6 (181.1)	254.9 (165.4)	255.8 (68.4)	256.1 (100)	275.4 (29.4)	310.1 (61.8)	307.6 (19.4)
			USEARCH61	6.8 (16.7)	16.1 (28.8)	40.7 (100.2)	29.5 (33.2)	36.4 (27)	24.8 (12.9)	38.7 (17.5)	38.1 (8.2)	49.3 (12)	52 (10.9)
			Mothur NB	NA (NA)	NA (NA)	NA (NA)	NA (NA)	NA (NA)	NA (NA)	NA (NA)	NA (NA)	NA (NA)	NA (NA)
			16S Classifier	203.8 (354.4)	291.8 (291.1)	240 (216.8)	249.6 (150.8)	251.7 (144.5)	241.7 (59.8)	238.9 (79.5)	252.9 (27.2)	251.4 (39.3)	251 (16.5)
			SINTAX	7.7 (16.1)	40.7 (81.1)	48.7 (117.2)	80.7 (84.4)	106.8 (120.5)	101.5 (50.6)	87.1 (35.5)	94.9 (16.7)	103.2 (21.2)	100.4 (17.4)
	V4	190	Karp	0.5 (1.9)	0.3 (1.1)	0.9 (4.2)	3.8 (10.2)	0.5 (0.6)	0.9 (1.4)	1.4 (1.2)	2.3 (1.8)	1.5 (1.3)	1.3 (0.3)
			Karp-Collapse	0.5 (2)	0.1 (0.2)	0.1 (0.3)	0.8 (2.1)	0.4 (0.6)	0.3 (0.3)	0.4 (0.4)	1.1 (2.7)	1 (1.5)	0.5 (0.3)
			UCLUST	<0.1 (0.1)	3.8 (18.2)	0.1 (0.6)	<0.1 (0.1)	5.1 (11.1)	0.8 (1.2)	2.7 (6.5)	3.4 (5.2)	4.7 (4.8)	6 (3)
			SortMeRNA	2 (6.6)	4.2 (18)	2.5 (11.4)	7.2 (19.1)	9.8 (15.3)	3.7 (8.6)	10.8 (17.7)	7.4 (7)	8.4 (10.7)	5.7 (2.5)
			Kallisto	5.4 (7.5)	14.5 (30)	20.9 (75.6)	28 (42.2)	66.8 (74.3)	79.4 (82.1)	106 (54)	107.2 (45.8)	101.6 (35.4)	107.6 (18.3)
			USEARCH61	0.6 (2.4)	0.1 (0.2)	0.1 (0.3)	3.3 (5.6)	1.8 (3.5)	2.3 (3.3)	2.6 (4)	3.6 (5.8)	5.7 (3.4)	6.2 (2.9)
			Mothur NB	303 (706.3)	48.3 (80.4)	68.8 (90.3)	43.4 (65.9)	109.6 (83.1)	99.2 (63.2)	126.5 (45.2)	108.3 (23.4)	115.5 (24.4)	98.1 (6.6)
			16S Classifier	160 (363.6)	152.5 (265.7)	111.2 (168.6)	60.8 (93.5)	84.7 (78.3)	81.6 (72.3)	101.3 (43.2)	85.5 (25.1)	93.6 (26)	79.5 (9)
			SINTAX	<0.1 (0.1)	72.1 (218.4)	0.2 (0.6)	58.3 (182.3)	10.2 (12.0)	36.6 (52.6)	35.2 (34.6)	23.8 (9.1)	33.2 (11.1)	28.7 (6.8)

SE=Single-end, PE=Paired-end

Table 5: **Per genus error rates, 1,000,000 reads**

Read Length	Region	Samples	Method	Shannon Diversity									
				[0,0.7)	[0.7,1.4)	[1.4,2)	[2,2.7)	[2.7,3.4)	[3.4,4.1)	[4.1,4.8)	[4.8,5.5)	[5.5,6.2)	[6.2,6.9]
75bp SE	Full gene	110	Karp	120.3 (97.5)	201.7 (165.9)	228.2 (46.5)	229.9 (63.1)	170.9 (32.5)	165.5 (35.2)	166.2 (35.3)	166.3 (27.7)	153.3 (19.4)	135.3 (23.6)
			Karp-Collapse	134 (116.8)	207.7 (164.1)	262.5 (64.8)	231.9 (69.7)	193.3 (29.6)	179.4 (37.4)	184.2 (35.6)	174.7 (21)	161.1 (14.4)	142.2 (18.4)
			UCLUST	374 (309.4)	488 (409.6)	557.2 (171.4)	503.8 (151.8)	430.7 (60.5)	402.9 (93)	386.3 (64.5)	362.1 (60.8)	330 (39.3)	279 (45.3)
			SortMeRNA	366.9 (296.9)	505 (426.9)	536.7 (141)	503.5 (136.2)	436.2 (54.8)	421.9 (77.5)	407.3 (68.4)	387.8 (57.5)	357.6 (36)	354 (220.2)
			Kallisto	246.1 (148.5)	324.4 (227.7)	388.9 (72.4)	379.1 (92.4)	297.5 (48.4)	291.7 (51.1)	281.7 (47)	274.8 (39.3)	251.7 (28.7)	218.9 (36.4)
			USEARCH61	400.7 (317.7)	517.9 (432.1)	562.4 (156.6)	513.7 (147.3)	441 (60.3)	419 (83.9)	399.5 (65.7)	374.5 (58.6)	338.5 (36.9)	288.2 (46.1)
			Mothur NB	409.9 (378.6)	454.2 (348.5)	529.2 (215.6)	596.1 (163.6)	553.9 (99.1)	586.4 (124.7)	540.2 (64.2)	544.1 (62)	533.5 (54.8)	536.6 (136.9)
			16S Classifier	1722 (648.2)	1190.5 (884.1)	1550.5 (424.4)	1799 (221.8)	1704.5 (220.7)	1679.8 (297.9)	1663 (202.4)	1654 (227.7)	1636.6 (236.6)	1595.3 (242.3)
			SINTAX	169.4 (210.8)	345.1 (324.9)	389.4 (163.4)	440.7 (123.4)	413.2 (81.9)	399.0 (115.0)	405.0 (49.9)	403.4 (71.7)	393.8 (59.2)	373.2 (62.2)
	V4	130	Karp	31.3 (32.9)	81 (77.5)	84.9 (49.9)	84.2 (37.9)	70.9 (29.5)	68.2 (13.6)	65.1 (16.5)	64.5 (11.5)	53.6 (4.8)	44.9 (3.9)
			Karp-Collapse	125 (201)	176.5 (125.3)	193.6 (81.4)	177.6 (60.8)	179.6 (52.9)	166.3 (20.7)	172.4 (28)	162.1 (24)	129.9 (12.7)	101.4 (4.8)
			UCLUST	112.2 (221.8)	161.9 (173)	155.2 (85)	158.2 (77.2)	152.9 (55.5)	141.2 (30)	147.3 (29.7)	144.9 (29.6)	116.7 (14)	91.9 (6)
			SortMeRNA	100.9 (209.6)	144.7 (149.2)	159.3 (103.1)	160.3 (87.3)	145.1 (57.4)	137.5 (18.5)	153.8 (35.2)	151.4 (22.3)	123.3 (13.2)	102.2 (6.8)
			Kallisto	71.3 (70.4)	156 (145.1)	141.2 (61.2)	145 (41.2)	135.1 (43.1)	126.8 (22.1)	124.6 (24.7)	122.1 (15.8)	102.3 (7.4)	85.7 (5.3)
			USEARCH61	114.5 (217.1)	170 (171.7)	175.2 (109.4)	172 (89.5)	154 (55.1)	148.7 (26.3)	152 (34.3)	151 (24.9)	120.4 (14.1)	96.6 (6.3)
			Mothur NB	198.2 (441.1)	225.9 (178.4)	325 (146.9)	321.6 (118.9)	330.6 (53.3)	325.4 (51.3)	332.6 (45.4)	316.9 (34.2)	310.9 (32.7)	285.7 (10.3)
			16S Classifier	613.6 (798.1)	652.4 (595.8)	658.2 (407.7)	691.6 (256.6)	669.5 (167)	677.7 (173.9)	680.6 (108.2)	663.1 (93.6)	714.5 (36.7)	693.3 (22.4)
			SINTAX	41.5 (54.0)	89.2 (79.9)	106.6 (71.8)	139.4 (64.7)	179.1 (62.3)	173.6 (37.9)	161.5 (40.8)	177.5 (33.6)	182.5 (17.9)	170.6 (7.9)
151bp PE	Full gene	130	Karp	4.5 (7.6)	8.8 (10)	8.3 (10.2)	9.5 (9.5)	12.4 (7.5)	12.6 (7.3)	12.6 (3.2)	14.8 (3.4)	15.3 (1.7)	14.6 (1.2)
			Karp-Collapse	17.7 (36.3)	57.5 (107.3)	17.6 (19.5)	51.7 (66.4)	40.8 (29.8)	36.2 (10.3)	43 (14.6)	45 (13.3)	48.3 (10.6)	49.7 (5.6)
			UCLUST	38.5 (63.6)	87.1 (132.5)	52 (86.4)	66.5 (83.2)	51.4 (28.5)	63.1 (33.5)	65.9 (16.6)	59.6 (13.4)	70.1 (10.7)	66.9 (4.3)
			SortMeRNA	53.1 (58.6)	110.7 (123.3)	87.5 (101.4)	113.9 (52.3)	105.3 (53.4)	116.4 (61.7)	100.5 (31)	102.6 (19.5)	99.4 (14.4)	87.7 (5.6)
			Kallisto	8.3 (11.5)	25.4 (21.8)	37.3 (25.9)	44.6 (27.7)	51.9 (11.8)	53.3 (8.4)	54.2 (11.9)	53.3 (5.2)	55.6 (4.5)	55.2 (2.8)
			USEARCH61	24 (28.9)	101 (122.2)	73.3 (89)	49.8 (39.7)	68.9 (48.8)	62.4 (24.6)	71.7 (17)	70.2 (15)	78 (12.5)	76.2 (5.9)
			Mothur NB	121.7 (90.7)	201.9 (139.5)	212.8 (94.8)	264.6 (92.3)	247.2 (95.3)	251.3 (95.2)	218.3 (53.1)	210.3 (19.5)	199.9 (24)	167.8 (13.7)
			16S Classifier	1393 (844.9)	1240.4 (645)	1306.4 (531.2)	1184.9 (346.1)	1345.6 (159.5)	1385.8 (130.9)	1352.3 (99.9)	1326 (45.9)	1347.4 (59.8)	1347.5 (15.5)
			SINTAX	37.9 (47.4)	99.2 (149.6)	87.0 (98.4)	113.1 (56.8)	118.8 (44.5)	133.6 (57.1)	130.5 (33.9)	126.8 (27.1)	136.3 (18.0)	126.4 (5.7)
	V4	190	Karp	0.2 (0.3)	2.4 (12)	4.2 (16.3)	0.7 (1.1)	2.1 (4.2)	1.6 (1.5)	3.1 (2.5)	2.5 (1)	2.4 (0.7)	3 (0.8)
			Karp-Collapse	0.2 (0.7)	0.4 (1.2)	4.2 (16.6)	0.6 (1.3)	1.9 (4.3)	2.5 (3.1)	1.7 (1.6)	1.8 (1.2)	1.3 (0.9)	2 (1)
			UCLUST	0.7 (2.1)	0.2 (0.3)	36.7 (174.8)	4.6 (7.8)	21.2 (46.4)	14.9 (25.4)	13.2 (19.1)	11.8 (12.9)	11.8 (7.9)	15.1 (4.6)
			SortMeRNA	7.8 (35.9)	14.7 (56.7)	19.4 (65.3)	15.6 (27.6)	18.3 (40.9)	33.8 (40.2)	25.6 (19.3)	27.1 (12.9)	29.1 (13.2)	31.7 (12.9)
			Kallisto	5.9 (7.3)	8.7 (10.2)	29.3 (26.3)	33 (19.1)	35.6 (41.5)	49 (39.2)	42.8 (8.6)	37.2 (8.2)	35.9 (3.8)	36.9 (2.4)
			USEARCH61	11.2 (62)	37 (173.1)	13.4 (62.1)	5.6 (9.1)	24.1 (46.6)	31.1 (47.2)	24.3 (16)	20.7 (18.6)	23.1 (9.7)	24.1 (7.3)
			Mothur NB	93.3 (86.2)	129.4 (95.4)	246 (196.5)	305.5 (117.4)	242.4 (47.3)	319.5 (38.1)	311.5 (41.2)	297 (36.2)	277.4 (16.7)	251.1 (11.4)
			16S Classifier	299.2 (653.9)	277.4 (402.6)	348.3 (365.4)	422.7 (396.9)	247.8 (118.7)	366.5 (193.1)	329.2 (84)	342.1 (83.2)	320 (56.8)	317.9 (33.6)
			SINTAX	3.7 (8.7)	14.6 (55.4)	87.9 (258.9)	60.7 (127.4)	36.1 (59.6)	80.9 (77.9)	60.2 (39.3)	50.8 (47.8)	36.6 (14.9)	40.1 (8.9)

SE=Single-end, PE=Paired-end

Table 6: **Per genus error rates, 1,000,000 reads**

Read Length	Region	Samples	Method	Shannon Diversity									
				[0,0.7)	[0.7,1.4)	[1.4,2)	[2,2.7)	[2.7,3.4)	[3.4,4.1)	[4.1,4.8)	[4.8,5.5)	[5.5,6.2)	[6.2,6.9]
301bp PE	Full gene	170	Karp	7.1 (20.1)	6 (11.2)	14.4 (17.4)	19.6 (23.7)	16.8 (16.2)	12.1 (4.4)	21.6 (9.1)	19.4 (2.2)	22.5 (2.7)	24.8 (2.5)
			Karp-Collapse	9.2 (10.2)	15.7 (28.5)	26.3 (37.5)	20.9 (20.6)	21.8 (23.3)	19.4 (9.3)	22 (11)	20.1 (4.5)	21.4 (3.6)	19.8 (2.3)
			UCLUST	23 (59)	15.9 (33)	30.3 (43.2)	27.8 (38)	42.3 (49.8)	38.3 (37.6)	42 (22.4)	37.7 (10.8)	47.7 (10.7)	40.5 (5.6)
			SortMeRNA	42.2 (88.1)	46.7 (64.8)	93.4 (85.5)	71.1 (55.1)	75.3 (71.8)	76.3 (35.1)	79.5 (24.4)	81.5 (20.9)	79.4 (10.1)	72.9 (7.5)
			Kallisto	313.5 (368.4)	468 (380)	636.5 (277.7)	688 (218.7)	782 (175.3)	741.7 (174.4)	816.1 (142.6)	792.6 (61.3)	825.7 (55)	814.9 (25)
			USEARCH61	21.2 (50.3)	18.7 (34.4)	41.8 (48)	27.4 (34.8)	34.4 (35.9)	24.2 (8.9)	44.1 (26)	44 (11.2)	48.5 (9)	50 (9)
			Mothur NB	NA (NA)	NA (NA)	NA (NA)	NA (NA)	NA (NA)	NA (NA)	NA (NA)	NA (NA)	NA (NA)	NA (NA)
			16S Classifier	1049.8 (861.4)	1272.6 (572.3)	1328.5 (462.9)	1188.2 (210.2)	1277.6 (279)	1168.9 (190)	1264.5 (178.9)	1232 (87.2)	1231.3 (39.9)	1223 (20.6)
			SINTAX	25.9 (36.1)	53.3 (97.8)	93.2 (91.2)	87.3 (59.1)	109.6 (88.6)	109.7 (68.9)	103.8 (48.5)	104.0 (13.8)	118.7 (18.0)	105.7 (11.3)
	V4	190	Karp	4.2 (20.9)	1.1 (1.4)	5.2 (8)	24 (41.4)	6.6 (7.7)	4.7 (2.6)	4.3 (1.3)	6.3 (3.3)	5.6 (1.3)	5.4 (1)
			Karp-Collapse	0.7 (2.5)	0.2 (0.8)	0.8 (1.8)	4.4 (13.3)	0.6 (0.7)	1 (1.5)	0.5 (0.4)	1.3 (2.9)	0.8 (0.5)	0.7 (0.5)
			UCLUST	0.1 (0.4)	2.9 (14.5)	1.5 (9.4)	5.3 (16)	3.8 (10.6)	4.8 (13.7)	2.7 (6.6)	1.2 (1)	6.4 (5)	7.9 (2.8)
			SortMeRNA	1.8 (5.7)	8.7 (31.1)	4.4 (15.4)	2.7 (3.1)	6.4 (15.9)	17.2 (41.6)	17.2 (28)	7.1 (7)	18.3 (12.1)	12.2 (3.7)
			Kallisto	69.8 (117.7)	207.2 (264.6)	430.5 (318.3)	565.1 (199.5)	533 (175.4)	592.3 (112.4)	561.6 (103.2)	550.4 (57.3)	570.4 (41.2)	558 (30.5)
			USEARCH61	0.8 (3.4)	1 (3.8)	3.5 (14.5)	0.5 (1)	4.7 (12.3)	10.1 (17.6)	5.8 (9.3)	6 (9.4)	7.2 (3.9)	9.4 (3.9)
			Mothur NB	133.2 (387.6)	207.9 (155.1)	393.5 (262.2)	308.4 (76.4)	317.8 (73.1)	309.4 (28.7)	306.9 (29.1)	292.3 (26.3)	275.6 (12.7)	242.1 (9.6)
			16S Classifier	28.4 (39.5)	342.1 (518.5)	433.8 (405.3)	396.7 (294.1)	344.8 (242.8)	367.7 (72.8)	329 (85.5)	324.3 (24.9)	353.1 (41.3)	329.6 (23.8)
			SINTAX	2.7 (7.1)	100.4 (363.6)	47.8 (118.9)	26.8 (44.0)	28.8 (51.7)	74.6 (125.3)	58.3 (38.8)	39.4 (21.0)	48.6 (16.9)	40.9 (8.0)

SE=Single-end, PE=Paired-end

Table 7: **Per family error rates, 1,000,000 reads**

Read Length	Region	Samples	Method	Shannon Diversity									
				[0,0.7)	[0.7,1.4)	[1.4,2)	[2,2.7)	[2.7,3.4)	[3.4,4.1)	[4.1,4.8)	[4.8,5.5)	[5.5,6.2)	[6.2,6.9]
75bp SE	Full gene	110	Karp	110.6 (113.4)	224.3 (142.9)	181 (73.5)	206.8 (84.7)	148.6 (28.3)	132.6 (16.7)	119.7 (9.1)	118.4 (10)	96 (10.8)	82 (6.9)
			Karp-Collapse	107 (112.6)	215.7 (137.3)	165.3 (69.9)	198.8 (83.6)	132.9 (18.9)	126.9 (15.5)	113.5 (8.1)	112.2 (11.7)	93.9 (11.1)	77.1 (7)
			UCLUST	279.5 (218)	467.2 (256.4)	390 (176.4)	388.7 (166.9)	293.5 (51.2)	265.6 (33)	233.4 (17.7)	216.2 (23.1)	167.6 (18.3)	132.7 (11.7)
			SortMeRNA	254.4 (195.7)	467.4 (273.8)	377.2 (179.3)	391.3 (149.9)	308.9 (52.1)	292.3 (35)	263.7 (17.9)	247.5 (24.2)	198.4 (21)	190 (114.3)
			Kallisto	271.7 (167.3)	415.9 (182.5)	354.8 (124)	366.2 (125.1)	290.2 (31.1)	260.1 (20.5)	224.5 (10.9)	208.9 (17.6)	166.6 (18.6)	140.6 (12.1)
			USEARCH61	287.7 (224.9)	497.8 (276.8)	399.7 (179.8)	402.7 (170.3)	306.5 (45.7)	282.6 (30.5)	244.7 (17.8)	227.2 (24.5)	175.1 (21.3)	138.3 (12.4)
			Mothur NB	327.3 (341.2)	433.9 (261.7)	433.9 (199.8)	510.9 (181.8)	392.8 (110.9)	416.4 (99.2)	405.4 (50.3)	404.8 (40.2)	382.7 (49.5)	382.5 (34)
			16S Classifier	1992.7 (44.6)	1941.3 (106.7)	1866.6 (267.6)	1946.1 (108.2)	1843.5 (148.6)	1912.7 (68.7)	1846.3 (57.8)	1834.4 (57.1)	1837.6 (57.9)	1853.3 (57.3)
			SINTAX	98.1 (99.2)	269.1 (177.0)	238.0 (100.9)	368.7 (140.5)	257.9 (53.1)	250.5 (43.5)	247.1 (26.8)	278.4 (21.1)	253.1 (22.3)	252.9 (10.2)
	V4	130	Karp	40.9 (55.3)	86.7 (92.1)	96.5 (63.2)	79.1 (23.1)	72 (25.3)	63.4 (14.4)	63.9 (15.4)	58.1 (4.4)	46.8 (5.6)	38.8 (2.7)
			Karp-Collapse	165.2 (270.1)	241.1 (173.6)	231 (104.8)	199.5 (54)	181.9 (40.6)	145.9 (27)	160.9 (43)	130.6 (10.6)	103.9 (15.5)	74.2 (4.8)
			UCLUST	130.2 (295.7)	166.8 (186.9)	157 (97.1)	146.3 (78.7)	124.5 (50.9)	99.4 (26.5)	119.5 (49.1)	95 (10.3)	75.6 (9.7)	51.2 (4.6)
			SortMeRNA	157.1 (341.8)	139.6 (167.5)	150.6 (101.1)	133.6 (61.5)	123.6 (49.5)	105.2 (22)	125.9 (40)	110.3 (10)	91.3 (14.7)	66.7 (5.2)
			Kallisto	104.1 (80.9)	205 (135.8)	201 (78.8)	180.8 (46.8)	165.3 (35.7)	144 (18.3)	142.4 (25.2)	126.6 (11)	101.6 (9.5)	79.6 (2.2)
			USEARCH61	149 (300.8)	172 (182.2)	175.9 (111.8)	142.1 (60.7)	130.6 (51.2)	105.1 (21.6)	122.5 (43.8)	103.3 (10.2)	78.5 (13.3)	55.3 (3.6)
			Mothur NB	311.4 (491.2)	404.8 (345.2)	498.2 (210.2)	399.5 (188.6)	397.4 (171.5)	423.9 (54)	433.3 (63.8)	408.8 (41.3)	374.4 (50.9)	344 (13.1)
			16S Classifier	1045.6 (710.8)	1440.5 (395.3)	1230.8 (400.5)	1266.7 (294.5)	1289.4 (179)	1100.4 (149.2)	1151.3 (98.8)	1130.1 (64.3)	1129.6 (61.3)	1113.8 (19.5)
			SINTAX	60.8 (57.0)	86.4 (41.1)	163.5 (100.3)	194.9 (96.5)	185.1 (48.4)	178.8 (32.9)	200.3 (41.8)	185.1 (19.2)	166.7 (10.6)	163.5 (6.1)
151bp PE	Full gene	130	Karp	2 (3.2)	5.3 (7.6)	7.4 (10.9)	8.4 (9.1)	9.3 (5.1)	10.4 (7.3)	9.4 (3.1)	11.1 (2.3)	11.7 (2.1)	10.5 (0.9)
			Karp-Collapse	6.3 (3.6)	20.8 (22.4)	17.8 (13.1)	26.8 (25)	28.6 (11.7)	27.3 (8.8)	27 (7.6)	24.7 (3.2)	23.6 (4.2)	22.1 (1.5)
			UCLUST	3.9 (3.5)	19.1 (31.3)	27.6 (45.9)	26.1 (20.7)	28.9 (18.6)	25.3 (14.5)	21.6 (5.3)	24.7 (3.8)	27.1 (6)	24.1 (2.8)
			SortMeRNA	7 (9.3)	31.2 (39.4)	39.1 (50.4)	41.2 (30.2)	40.9 (26.8)	38 (17.9)	31.2 (8.8)	35.3 (5.8)	33.8 (5.4)	28.8 (2.1)
			Kallisto	14.1 (12.1)	46.1 (24.1)	51.8 (22)	70.6 (19.3)	76.2 (16.9)	71.2 (10.8)	74.7 (7.4)	72.3 (4.9)	71.9 (5)	70.4 (2.7)
			USEARCH61	9.2 (5.2)	26.7 (37.6)	43.8 (57.7)	35.5 (26.9)	40.5 (25.5)	35.4 (13.3)	31.7 (5.6)	33.9 (4.6)	34.9 (7.3)	31.5 (2.2)
			Mothur NB	135.1 (52.8)	168.5 (59.9)	207 (108.5)	169.8 (72)	157.3 (48.2)	146.2 (57.7)	89.2 (21)	83.9 (17.9)	76.5 (11.7)	59.8 (6.3)
			16S Classifier	1873.9 (215.5)	1821.7 (284.1)	1821.5 (278.6)	1799.6 (172.9)	1675.5 (168.9)	1663.9 (85.2)	1625.2 (83)	1666.5 (24.1)	1645.3 (32.9)	1646.5 (6.8)
			SINTAX	9.9 (10.8)	51.8 (191.6)	36.2 (60.1)	45.5 (32.3)	42.4 (29.2)	34.7 (18.1)	30.6 (11.8)	29.9 (5.2)	32.5 (6.2)	28.0 (2.7)
	V4	190	Karp	0.5 (0.9)	2.9 (12.9)	5.2 (18.4)	1.3 (1.5)	2.5 (4.3)	2.1 (1.5)	3.7 (2.5)	3 (1)	3 (0.7)	3.7 (0.8)
			Karp-Collapse	0.2 (0.7)	0.5 (1.6)	5.1 (18.7)	1 (1.8)	2.1 (4.7)	1.3 (1.6)	1.7 (1.5)	2 (1.3)	1.6 (1)	2.4 (1.1)
			UCLUST	0.9 (2.4)	1.2 (3.1)	41.4 (174.9)	5.7 (7.5)	9.3 (17.7)	23.8 (35.5)	23.9 (24.8)	25.9 (32.4)	13.6 (6.3)	16.2 (4)
			SortMeRNA	2.7 (5.8)	10.5 (52.9)	21.9 (76)	17.2 (30)	10.1 (17.8)	31.2 (46.9)	30.4 (22.9)	39.5 (32.2)	35.1 (12.7)	30.6 (5.8)
			Kallisto	14.9 (16.9)	28.4 (23)	54 (26.4)	58.7 (15.6)	53.6 (11.7)	61.2 (11.1)	66 (8.2)	60.9 (6.8)	59.4 (5.2)	59.2 (2.2)
			USEARCH61	38.5 (227.6)	10.3 (53)	21.7 (76.1)	16.9 (30.2)	10.4 (17.2)	35.1 (49.3)	29.2 (23.1)	34.1 (35.3)	27.4 (8.9)	26.4 (4.8)
			Mothur NB	192.6 (337.3)	212.8 (171.7)	288.2 (267)	310.4 (201)	319 (88.7)	276.7 (53.4)	309.4 (35.4)	254.1 (36.3)	206.5 (18.5)	138.2 (12.5)
			16S Classifier	555.2 (799.3)	204.1 (349.5)	385.9 (434.3)	368.2 (250.2)	335.6 (214.1)	326.9 (111.0)	296.2 (81.7)	263.3 (48.9)	279.3 (26.2)	247.5 (22.7)
			SINTAX	3.3 (9.4)	0.8 (1.9)	48.1 (213.0)	0.5 (0.7)	56.1 (101.6)	17.8 (30.4)	9.6 (9.9)	16.9 (11.4)	9.7 (6.1)	13.5 (3.4)

SE=Single-end, PE=Paired-end

Table 8: **Per family error rates, 1,000,000 reads**

Read Length	Region	Samples	Method	Shannon Diversity									
				[0,0.7)	[0.7,1.4)	[1.4,2)	[2,2.7)	[2.7,3.4)	[3.4,4.1)	[4.1,4.8)	[4.8,5.5)	[5.5,6.2)	[6.2,6.9]
301bp PE	Full gene	170	Karp	7.2 (20.6)	5.3 (11.1)	9.2 (15.8)	11.7 (15.3)	11 (14.6)	12.6 (6)	12.2 (2.6)	14.4 (4.3)	15.9 (2.5)	17.3 (1.3)
			Karp-Collapse	8.7 (11)	7.3 (5.1)	8.6 (4)	13.1 (13.5)	12.2 (13.2)	16.3 (16.5)	10.6 (2.9)	10.8 (1.7)	12.1 (2.4)	11.7 (2.1)
			UCLUST	16.1 (54.8)	3.7 (8.1)	7.9 (18.9)	2.6 (6.1)	5.4 (7.4)	7 (8.8)	9.1 (9.7)	9.5 (4)	7.9 (2.6)	9.6 (2.7)
			SortMeRNA	9.6 (22.9)	16.6 (29.9)	16.8 (25.6)	12.2 (16.1)	19.9 (19.6)	20.6 (15.5)	18.4 (7.8)	23 (8.2)	16.2 (4.5)	16.3 (2.6)
			Kallisto	448.6 (375.8)	796.4 (302)	908.1 (253.8)	981.1 (252.7)	1053.6 (128.8)	1037.5 (123.2)	1067.3 (108.7)	1039 (56.3)	1055.4 (31)	1008.5 (23.7)
			USEARCH61	10 (40.1)	4.8 (10.7)	12.8 (30.4)	6.8 (13.2)	10.6 (14.4)	13.3 (15)	10.3 (11.6)	12.3 (9)	11.1 (4.3)	12.1 (2.1)
			Mothur NB	NA (NA)	NA (NA)	NA (NA)	NA (NA)	NA (NA)	NA (NA)	NA (NA)	NA (NA)	NA (NA)	NA (NA)
			16S Classifier	1545.3 (609.4)	1550.6 (460.4)	1602.6 (296.6)	1502.9 (266.1)	1534.1 (148.4)	1506 (113.6)	1507.8 (43.9)	1431.6 (69.8)	1419.6 (31.1)	1404.9 (13.7)
			SINTAX	4.9 (13.0)	10.1 (20.7)	12.9 (25.0)	10.9 (23.8)	19.9 (19.9)	18.8 (15.2)	17.3 (10.0)	22.3 (6.5)	16.7 (4.7)	18.1 (3.2)
	V4	190	Karp	10.4 (53.9)	2.8 (2.7)	6.9 (8.3)	28.2 (46.2)	8.5 (7.8)	6.3 (2.8)	6.5 (2.2)	8.1 (4.2)	7 (1.4)	6.7 (1.1)
			Karp-Collapse	0.7 (3.2)	0.3 (0.7)	0.3 (0.6)	5.4 (16.3)	0.4 (0.4)	0.4 (0.2)	0.4 (0.2)	0.5 (0.1)	0.6 (0.4)	0.4 (0.1)
			UCLUST	0.1 (0.6)	0.4 (2.6)	9.9 (62.6)	<0.1 (<0.1)	<0.1 (<0.1)	0.2 (0.4)	0.6 (0.9)	0.4 (0.5)	1.4 (1.4)	2.8 (3.1)
			SortMeRNA	0.9 (3.3)	0.3 (0.9)	1.2 (5.7)	29.9 (91.3)	15.7 (47.6)	9.6 (28)	4.6 (6.3)	4.3 (4.8)	3.9 (3.2)	3.5 (2.4)
			Kallisto	238.4 (274)	536.3 (426.2)	774.3 (317.3)	989.2 (211.7)	958.5 (207.2)	1032.7 (110.9)	997.7 (142.1)	975.5 (82.1)	978.7 (58.5)	968.1 (25)
			USEARCH61	<0.1 (0.1)	<0.1 (<0.1)	<0.1 (<0.1)	0.1 (0.3)	4.1 (13)	4.9 (14.9)	2.8 (8.5)	1.1 (2)	3.8 (4.1)	3.2 (2.2)
			Mothur NB	325.2 (516.6)	196.8 (80.5)	364.2 (291)	298.3 (117.3)	313.7 (56.7)	260.9 (54.5)	265.5 (29.4)	238.3 (21.4)	212.7 (24)	147.6 (11.7)
			16S Classifier	281.0 (555.8)	325.1 (528.0)	345.1 (342.8)	395.3 (304.7)	245.2 (163.2)	311.6 (118.9)	299.0 (82.9)	271.8 (56.3)	264.8 (34.6)	255.0 (18.2)
			SINTAX	0.6 (2.3)	39.6 (122.7)	1.0 (2.8)	0.4 (0.7)	26.1 (61.2)	11.9 (35.2)	9.4 (14.6)	13.9 (8.2)	14.7 (12.6)	10.0 (3.7)

SE=Single-end, PE=Paired-end

Table 9: **Per order error rates, 1,000,000 reads**

Read Length	Region	Samples	Method	Shannon Diversity									
				[0,0.7)	[0.7,1.4)	[1.4,2)	[2,2.7)	[2.7,3.4)	[3.4,4.1)	[4.1,4.8)	[4.8,5.5)	[5.5,6.2)	[6.2,6.9]
75bp SE	Full gene	110	Karp	64.3 (48)	99.4 (57.8)	122.2 (51.2)	124.4 (43.9)	97.8 (14)	79 (12.7)	64.2 (8.8)	64.3 (4.6)	56.2 (4.6)	49.4 (3.6)
			Karp-Collapse	63.2 (46.2)	93.3 (54.3)	117 (52.5)	114.4 (38.8)	89.5 (13.8)	76 (13.1)	60.7 (10.2)	57.5 (5.8)	54.2 (4)	44.6 (3)
			UCLUST	161.7 (93.4)	222.9 (116.5)	258.1 (117.9)	211.1 (89.2)	193.1 (36.3)	149.8 (30.3)	117.2 (15.4)	108.4 (11.6)	84.1 (7.8)	69.2 (7.6)
			SortMeRNA	164.7 (88.6)	223.2 (119.5)	251.7 (113.9)	218.9 (79.7)	213.5 (34.2)	171.1 (32.5)	144.5 (23.3)	131.1 (16.4)	106.6 (8.7)	106.7 (69.1)
			Kallisto	194.9 (80.2)	256.4 (95.2)	269.1 (103)	236.3 (69.9)	212.5 (29.4)	166.9 (15.3)	135.3 (15.7)	124.9 (13.9)	102.3 (7.8)	89.8 (9.2)
			USEARCH61	175 (95.4)	246.1 (127)	274.9 (123.8)	223 (97.4)	204.7 (31.5)	161.8 (31.8)	127.4 (16.3)	117.7 (14)	91.7 (6.9)	74.8 (8.6)
			Mothur NB	269.2 (309)	313.1 (247.7)	412.6 (235.6)	318.6 (74.9)	301.3 (72.2)	298.6 (96.1)	304 (44.6)	282.1 (47.5)	279.9 (42.2)	289.3 (24.5)
			16S Classifier	1861.1 (80.4)	1661 (240.2)	1533.3 (369.8)	1509.2 (209.8)	1535.3 (232.4)	1438 (136.6)	1351 (102.1)	1332.7 (83.4)	1317.6 (70.8)	1357.9 (54.4)
			SINTAX	44.2 (28.5)	131.4 (63.8)	209.3 (107.3)	229.0 (89.1)	212.7 (42.6)	187.0 (36.5)	202.1 (23.9)	210.8 (11.4)	199.2 (15.2)	204.1 (9.0)
	V4	130	Karp	34.2 (51.4)	60.5 (74.3)	61.8 (46)	61.5 (25.2)	50.3 (14.4)	52.3 (12.5)	48.6 (13.7)	43.3 (9.2)	37 (5.1)	33.5 (1.7)
			Karp-Collapse	166.9 (273.6)	176.7 (116.8)	159.8 (91.7)	135.9 (54.2)	125.3 (37.6)	104.3 (25.9)	102.5 (40.4)	83.7 (10.4)	68.9 (7.7)	51 (3.9)
			UCLUST	124 (307.2)	99.1 (138.9)	87.7 (83.7)	66 (53.3)	59.8 (28.4)	50.4 (12.3)	57.3 (42.1)	46.3 (4.4)	37.5 (4.5)	28.5 (3.7)
			SortMeRNA	143.8 (344.9)	82.6 (104.6)	80 (67.4)	68.6 (49)	72.2 (27.1)	66.4 (15.7)	72.6 (35.2)	62.6 (4.2)	59.3 (7.6)	49.9 (4.2)
			Kallisto	99.2 (84.1)	165.2 (117.8)	159.9 (87.7)	130.7 (46.1)	118.7 (34.4)	114 (18.1)	111.9 (19.8)	100.9 (6)	85.3 (6.2)	74.6 (1.5)
			USEARCH61	133.4 (310.3)	104 (138.3)	99 (94.2)	71.9 (57.2)	67.4 (30.2)	57.1 (13.3)	60.5 (34.2)	54.1 (5.9)	42.7 (6.2)	32.7 (3.7)
			Mothur NB	372.8 (484.3)	369.9 (277.3)	487.4 (250.4)	435.3 (81)	415.5 (129.2)	462.6 (134.4)	410.9 (46.2)	393.5 (45.5)	356.1 (43.7)	333.2 (11)
			16S Classifier	1374 (578.8)	1459.5 (400.8)	1277.8 (381.3)	1155.3 (265)	1164.7 (216.7)	1140.2 (113.3)	1060.4 (85.6)	1068 (118.3)	1038.7 (50.7)	1023.9 (23.6)
			SINTAX	62.6 (64.7)	115.3 (67.7)	180.3 (151.7)	174.7 (92.1)	159.3 (51.1)	159.3 (27.2)	173.7 (43.7)	173.6 (16.8)	166.1 (15.4)	165.0 (5.1)
151bp PE	Full gene	130	Karp	1.4 (2)	5.2 (7.6)	6 (9.9)	4.6 (2.8)	8.1 (4.9)	8.8 (7)	7.5 (2.8)	8.5 (1.6)	8.4 (1.6)	7.1 (0.8)
			Karp-Collapse	5.9 (3.9)	18.7 (21.2)	16 (11.6)	16.6 (7.7)	25.7 (11)	22.7 (7.2)	21.7 (6.3)	18.1 (3.6)	15.7 (3.3)	13.9 (1)
			UCLUST	3.1 (3.7)	15.4 (29.5)	9.8 (12.4)	18.4 (14)	16 (5.4)	16.3 (12.9)	15.9 (5.2)	14.2 (3.9)	13.9 (4.2)	13.2 (1.1)
			SortMeRNA	3.5 (3.7)	27.2 (59.5)	15.6 (16.1)	24.1 (23.3)	26.9 (19.6)	17.7 (7.1)	18.1 (6.6)	18.7 (3)	17.1 (3.2)	15 (1.3)
			Kallisto	14 (12.1)	45.8 (24.5)	49.4 (23.4)	69.6 (20.2)	74.6 (17.6)	68.5 (11.4)	69.4 (8.7)	68.4 (5.5)	68 (5)	67.3 (3.3)
			USEARCH61	8.6 (5)	20.3 (35.5)	19.3 (25.8)	17.8 (5.3)	30.7 (16.3)	25.9 (11)	22.5 (7.4)	21.7 (4.8)	19.3 (5.6)	16.9 (2.4)
			Mothur NB	124.1 (49)	158.7 (38.5)	181.6 (100.1)	138 (61.4)	124.3 (26.8)	101.7 (63.1)	56.6 (11.6)	45.6 (10.5)	38 (7.4)	31.2 (3.9)
			16S Classifier	1463.4 (501.1)	1595.9 (330.7)	1315.6 (343.1)	1423.4 (275.3)	1179 (270)	1280.7 (169.5)	1260.5 (117.9)	1250.6 (54.6)	1189.3 (50.9)	1206.5 (20.7)
			SINTAX	4.6 (5.5)	48.2 (190.4)	10.7 (16.3)	19.9 (21.2)	22.5 (23.0)	12.9 (12.4)	15.0 (6.7)	11.5 (3.0)	11.8 (3.0)	11.7 (1.2)
	V4	190	Karp	0.5 (0.9)	3.1 (13.5)	5.4 (18.7)	1.4 (1.5)	2.6 (4.3)	2.2 (1.8)	3.8 (2.2)	2.8 (1.1)	2.9 (1.1)	3.4 (0.9)
			Karp-Collapse	0.1 (0.3)	0.5 (1.5)	5.3 (19)	1.1 (1.8)	2.1 (4.7)	1.2 (1.6)	1.6 (1.4)	1.8 (1.2)	1.4 (0.9)	2.1 (1)
			UCLUST	2.6 (6.8)	19.6 (84.2)	51 (177.6)	5.9 (7.5)	22.4 (37.7)	33.6 (38)	29.4 (19.3)	27.8 (26.4)	20.9 (10.4)	17.7 (3.2)
			SortMeRNA	4.3 (8.2)	28.9 (98.2)	31.7 (84.5)	15.2 (23.7)	29.1 (38.9)	41.8 (46.9)	35.4 (16.8)	39.9 (28.2)	35.5 (8.5)	28.8 (4.5)
			Kallisto	21.5 (19.7)	51.5 (37.4)	62.9 (28.2)	67.3 (13.6)	70.1 (16.5)	80 (13.8)	78.6 (9.2)	71.9 (5.9)	70.5 (3.6)	72.4 (2.6)
			USEARCH61	4.1 (8.1)	28.9 (98.2)	31.4 (84.6)	15 (23.9)	29.1 (38.8)	41.7 (47.2)	34.6 (17.4)	36.3 (29.4)	29.6 (8.3)	25.4 (3.4)
			Mothur NB	186.7 (338)	220 (308.9)	188 (240.4)	277.6 (219.4)	335.7 (140.9)	252.4 (63.6)	262.4 (49.6)	203.5 (24.8)	148.6 (14)	93.4 (10.6)
			16S Classifier	421.7 (719.2)	128.1 (296)	179.8 (298.2)	347 (246.2)	259.3 (229.1)	299 (110.6)	195.2 (56.1)	231.5 (55.5)	239.6 (34.2)	219.7 (17.2)
			SINTAX	0.2 (0.4)	0.1 (0.3)	47.9 (213.3)	0.3 (0.5)	22.3 (69.3)	1.7 (1.9)	4.4 (7.1)	6.6 (10.2)	3.2 (1.6)	5.4 (3.3)

SE=Single-end, PE=Paired-end

Table 10: **Per order error rates, 1,000,000 reads**

Read Length	Region	Samples	Method	Shannon Diversity									
				[0,0.7)	[0.7,1.4)	[1.4,2)	[2,2.7)	[2.7,3.4)	[3.4,4.1)	[4.1,4.8)	[4.8,5.5)	[5.5,6.2)	[6.2,6.9]
301bp PE	Full gene	170	Karp	2.5 (4)	5.1 (11.1)	4 (4.6)	10.2 (14.5)	6.8 (5.1)	10.3 (7.2)	9.1 (2.2)	9.5 (3)	10.6 (2.4)	10 (0.6)
			Karp-Collapse	7.7 (9.4)	7 (5.2)	7.7 (3.7)	12.4 (13.3)	8.2 (3.3)	13.9 (16.6)	8.4 (3.1)	7.9 (1.3)	9.2 (3.5)	7.8 (1.2)
			UCLUST	0.6 (0.6)	3.1 (7.2)	3.6 (10)	0.7 (0.7)	1.7 (3.9)	0.6 (0.5)	3.5 (6.8)	4.3 (4.3)	2.7 (1.9)	3.3 (0.9)
			SortMeRNA	1.9 (4.2)	14.5 (29.2)	9.8 (20.1)	6.4 (14)	7.7 (9)	10 (13.1)	8.1 (5.3)	11.9 (9.8)	8 (4)	6.9 (1.5)
			Kallisto	424.4 (355.2)	778.8 (311.9)	883.6 (263.2)	972.5 (260)	1034.9 (139.8)	1022.5 (125.7)	1028.7 (118)	982 (63.5)	1006.9 (44.8)	961.9 (28.8)
			USEARCH61	0.4 (0.4)	3.8 (10.5)	3.4 (12.7)	5.1 (12.5)	3.7 (4.1)	8 (14.6)	7.1 (11.3)	6.4 (7.6)	5.3 (3.9)	4.8 (2)
			Mothur NB	NA (NA)	NA (NA)	NA (NA)	NA (NA)	NA (NA)	NA (NA)	NA (NA)	NA (NA)	NA (NA)	NA (NA)
			16S Classifier	1450.8 (557.7)	1246 (555.4)	1445.1 (305.4)	1268.7 (292.5)	1244.6 (190.6)	1218.4 (169.2)	1162.5 (110.2)	1070.7 (139)	1103.1 (60.3)	1090.2 (14)
			SINTAX	0.5 (1.1)	8.5 (20.4)	6.1 (15.1)	3.4 (5.9)	7.9 (9.5)	7.3 (13.0)	5.8 (7.3)	10.1 (7.9)	6.0 (2.4)	6.7 (1.5)
	V4	190	Karp	10.8 (54)	6 (11.3)	8.4 (8.9)	29.5 (49)	8.6 (7.8)	7 (3.9)	6.3 (1.9)	7.4 (3.5)	6.5 (1.4)	5.5 (1)
			Karp-Collapse	0.3 (1.9)	0.3 (0.9)	0.3 (0.5)	5.8 (17.6)	0.4 (0.4)	0.4 (0.3)	0.3 (0.2)	0.3 (0.1)	0.4 (0.2)	0.3 (0.1)
			UCLUST	<0.1 (<0.1)	0.4 (2.6)	14.1 (89.3)	<0.1 (<0.1)	<0.1 (<0.1)	0.1 (0.4)	0.2 (0.7)	<0.1 (<0.1)	0.1 (0.4)	0.9 (1.6)
			SortMeRNA	0.1 (0.1)	0.1 (0.2)	0.1 (<0.1)	32 (99.7)	15.3 (47.7)	9.2 (28.1)	0.8 (1)	1.8 (3.3)	0.9 (1.1)	1.6 (1.2)
			Kallisto	433.9 (358)	829.3 (396.8)	942.4 (299.1)	1117.8 (212.9)	1226.9 (192.2)	1186.1 (114.5)	1199.5 (107.4)	1171.1 (76.2)	1129.7 (72.7)	1159.8 (28.7)
			USEARCH61	<0.1 (<0.1)	<0.1 (<0.1)	<0.1 (<0.1)	0.1 (0.3)	<0.1 (<0.1)	4.7 (14.9)	2.7 (8.5)	<0.1 (<0.1)	0.7 (2.1)	1.4 (1.8)
			Mothur NB	453.4 (590.8)	268 (140.8)	321.1 (250.6)	267.8 (116.2)	211.2 (70.8)	230.8 (56.7)	181.6 (16.7)	157.2 (22.9)	145.4 (22.5)	84.9 (12.9)
			16S Classifier	264.4 (624.3)	141.4 (361.1)	182.8 (297.6)	155.1 (194.5)	163.1 (99.7)	212.9 (80.2)	201.3 (79)	214.3 (45.4)	211.3 (54.8)	203.6 (11.2)
			SINTAX	<0.1 (0.2)	<0.1 (0.1)	28.2 (126.3)	0.3 (0.5)	20.6 (61.1)	11.2 (35.4)	3.0 (7.3)	1.9 (3.0)	1.7 (2.4)	3.4 (2.3)

SE=Single-end, PE=Paired-end

Table 11: **Per class error rates, 1,000,000 reads**

Read Length	Region	Samples	Method	Shannon Diversity									
				[0,0.7)	[0.7,1.4)	[1.4,2)	[2,2.7)	[2.7,3.4)	[3.4,4.1)	[4.1,4.8)	[4.8,5.5)	[5.5,6.2)	[6.2,6.9]
75bp SE	Full gene	110	Karp	44.5 (22.4)	51 (18.5)	60.1 (27.4)	71.6 (22.5)	46.5 (11)	44.4 (11.5)	36.7 (5.4)	38.4 (4)	35.5 (3.6)	34.8 (2.7)
			Karp-Collapse	44 (23.4)	48.2 (16.7)	62.4 (26.2)	60.8 (15.8)	40.5 (12.1)	41.3 (11.2)	33.6 (5.2)	33.8 (4.2)	31.6 (3)	29.2 (2.1)
			UCLUST	109.1 (46.6)	121.5 (50.7)	124.2 (60.1)	94.5 (39)	82.4 (16.3)	70.1 (16.9)	53.8 (8)	50.1 (8.5)	39.2 (3.9)	34.5 (5.5)
			SortMeRNA	119.1 (58.1)	127 (56.6)	123.5 (49.7)	107.1 (40.6)	102.5 (18.7)	90.3 (26.9)	76.6 (11.1)	68.7 (12.2)	57.1 (5.5)	62.6 (43.4)
			Kallisto	161.9 (48.5)	160.1 (41.8)	150.8 (50.5)	147.2 (43.5)	113.4 (21)	99.9 (16.7)	83.2 (10.3)	79.1 (7.5)	67.7 (5.9)	64.8 (7.5)
			USEARCH61	118.2 (48.3)	136.9 (63.9)	132.7 (59.4)	99.7 (40.5)	91.3 (19.7)	78.7 (17.2)	60.7 (10.9)	57.1 (9.8)	46.9 (4.9)	41.1 (6.2)
			Mothur NB	271 (288.5)	256.4 (208.2)	228.4 (119.4)	196 (78)	215.3 (62)	231.4 (101.4)	233.8 (39)	221.7 (45)	217.7 (40.9)	229.2 (26.9)
			16S Classifier	1804.8 (125.8)	1479.4 (314.9)	1336 (450.7)	1339.5 (294.4)	1292.2 (290.4)	1177.6 (177.8)	1156.4 (112.3)	1092.8 (91.3)	1112.8 (71.6)	1155.5 (52.3
			SINTAX	33.7 (26.3)	91.8 (40.9)	104.6 (35.6)	100.0 (29.3)	86.9 (35.9)	97.4 (21.7)	94.3 (17.1)	92.1 (10.7)	81.7 (14.7)	85.8 (6.4)
	V4	130	Karp	25.7 (31.4)	26 (18.8)	30.1 (20)	44.6 (12.5)	34.1 (9.7)	38.1 (9.9)	32.8 (4)	31.8 (7)	26.9 (3.9)	26.4 (1.9)
			Karp-Collapse	136.3 (203.8)	86.1 (63.9)	85.8 (23.8)	78.7 (27.5)	67.1 (20.1)	59.7 (16.1)	47.4 (4.9)	44.4 (11.7)	37.7 (4.7)	29.2 (2.9)
			UCLUST	76.9 (194)	26.8 (42.8)	26.4 (11.4)	26 (12.7)	23 (6.8)	20.9 (4.7)	17.4 (4.5)	16 (4.3)	13.4 (1.9)	11.5 (1.5)
			SortMeRNA	107.8 (282.9)	28.5 (35.4)	33.2 (14.7)	32.6 (12.4)	37.2 (10.1)	38.7 (9.8)	33.8 (6)	33.3 (5.3)	36.3 (5.7)	30.7 (2.2)
			Kallisto	78.3 (61.8)	82.8 (48.3)	91.5 (27.3)	81.3 (23.4)	75.7 (13.6)	76.1 (13.3)	74.7 (6.7)	69.3 (8)	64 (5.1)	62 (1.9)
			USEARCH61	94.9 (240.5)	29.9 (41.6)	33.7 (24.5)	30.4 (12.4)	25.5 (8.8)	21.5 (6.1)	18.3 (4.7)	19.2 (4)	16.4 (2.9)	12.7 (1.9)
			Mothur NB	331.1 (341.6)	279.9 (208.2)	328.4 (165.6)	353.7 (95.6)	318 (72.7)	345.9 (81.7)	324.9 (60.2)	339.4 (29.5)	332 (25.7)	310.7 (10.6)
			16S Classifier	1339 (559.1)	1317.8 (386)	1160.2 (344.3)	1066.2 (243.3)	1050.8 (202.2)	1041.2 (99.2)	944.7 (77.1)	976.5 (115.2)	950.2 (44.6)	931.7 (27.2)
			SINTAX	69.1 (96.6)	77.3 (62.0)	72.5 (38.5)	87.4 (32.2)	67.8 (14.5)	64.4 (12.6)	61.8 (13.8)	66.6 (9.4)	67.9 (8.0)	63.0 (5.4)
151bp PE	Full gene	130	Karp	1.6 (2.5)	4.8 (7.6)	5.6 (9.6)	3.7 (2.5)	6.4 (4)	6.7 (5.9)	5.4 (2.7)	5.8 (1.4)	6 (1.4)	5 (0.5)
			Karp-Collapse	4.9 (3.5)	17.9 (21.2)	15.4 (11.3)	14.8 (8.2)	17.1 (5.9)	19.7 (6.5)	16.3 (6.2)	14 (3)	10.1 (1.9)	9.3 (0.8)
			UCLUST	1.9 (1.7)	3.8 (6.5)	4.4 (5.1)	9.9 (3.8)	10.1 (4.1)	9 (1.8)	9.2 (2.6)	7.7 (1.1)	6.9 (1)	7.1 (0.8)
			SortMeRNA	2.1 (1.4)	3.9 (3.3)	5.6 (3.7)	9.1 (3.4)	10.3 (4.1)	11.3 (6.5)	9.7 (2.2)	9.5 (2.8)	9.2 (1)	8.5 (0.9)
			Kallisto	14 (12.1)	45.5 (24.6)	48.4 (23.6)	67.4 (21.5)	69.5 (16.1)	63.6 (14.2)	62.1 (8.1)	63.7 (6.2)	61.3 (4.2)	62.1 (2.6)
			USEARCH61	7.5 (4.6)	12.1 (30.6)	10.5 (9.3)	15.8 (5.6)	20.2 (4.8)	18.8 (7.7)	13.5 (3.8)	12.2 (2.3)	11.3 (1.9)	9.5 (1.5)
			Mothur NB	123.4 (49.5)	148.7 (32.1)	132 (34.7)	129 (66)	94.4 (25.9)	80.6 (52.7)	34.2 (10)	25.7 (4.1)	18.7 (3.9)	14.1 (1.9)
			16S Classifier	1378.1 (455)	1479.9 (322.6)	1194.7 (367.4)	1250.5 (299.2)	1000.7 (262.9)	1111.1 (163.7)	1092.7 (99.3)	1082.8 (43.8)	1033.2 (49.4)	1042.6 (22.1)
			SINTAX	1.0 (1.6)	35.7 (190.9)	1.9 (1.8)	2.6 (1.0)	2.3 (1.0)	2.3 (0.7)	3.8 (1.9)	2.3 (0.4)	2.4 (0.8)	2.2 (0.4)
	V4	190	Karp	0.5 (0.9)	3.1 (13.5)	5.4 (18.7)	1.3 (1.5)	2.4 (4.3)	1.9 (1.8)	3.3 (2.1)	2.3 (1)	2.4 (1.2)	2.7 (0.9)
			Karp-Collapse	0.1 (0.3)	0.5 (1.5)	5.2 (19)	1 (1.8)	2 (4.6)	1.1 (1.5)	1.4 (1.3)	1.6 (1.1)	1.3 (0.8)	2 (1)
			UCLUST	2.4 (6.7)	19.6 (84.2)	23.3 (67.7)	5.6 (7.3)	21.9 (37.3)	31.6 (36.9)	27.6 (19.2)	24.2 (20.9)	18.2 (8.5)	14.8 (3.2)
			SortMeRNA	4 (8.2)	28.9 (98.2)	30 (78.7)	12.9 (17.5)	28.5 (38.6)	39.6 (45.5)	32.7 (17.2)	32.8 (22)	29.6 (5.9)	24.6 (5.6)
			Kallisto	21.3 (19.8)	51 (37.6)	61.2 (27.7)	63.4 (16.7)	67.7 (16.2)	74.9 (12)	74.1 (9.3)	67.5 (5.9)	66 (3.5)	68.3 (1.9)
			USEARCH61	3.9 (8.2)	28.9 (98.3)	29.6 (78.8)	12.8 (17.6)	28.6 (38.6)	38.9 (46)	32.1 (17.6)	31.1 (21.8)	23.8 (6.5)	21.6 (4.5)
			Mothur NB	99.6 (28)	152.7 (170.3)	168.6 (244.7)	224.2 (180.7)	235.9 (89.6)	196.8 (56.9)	186.7 (39.9)	137.4 (28.9)	95.1 (17)	53.8 (12.8)
			16S Classifier	299.9 (653.2)	117.2 (283.8)	83.3 (188.1)	285.5 (228.6)	229.3 (231.7)	178.8 (68)	152.1 (49.2)	159.3 (47.6)	171.7 (31.7)	154.8 (20.6)
			SINTAX	<0.1 (0.1)	0.1 (0.3)	0.1 (0.3)	<0.1 (0.1)	0.3 (0.3)	0.7 (0.8)	0.9 (1.4)	1.4 (2.6)	0.7 (0.6)	1.7 (1.7)

SE=Single-end, PE=Paired-end

Table 12: **Per class error rates, 1,000,000 reads**

Read Length	Region	Samples	Method	Shannon Diversity									
				[0,0.7)	[0.7,1.4)	[1.4,2)	[2,2.7)	[2.7,3.4)	[3.4,4.1)	[4.1,4.8)	[4.8,5.5)	[5.5,6.2)	[6.2,6.9]
301bp PE	Full gene	170	Karp	2.4 (4)	5.4 (11.2)	2.9 (2.7)	5.8 (7.4)	5.1 (4.4)	5.1 (1.5)	6.4 (1.8)	5.8 (1.2)	6.7 (1.5)	6.1 (1)
			Karp-Collapse	7.4 (9.5)	6.2 (4.5)	7.4 (3.8)	8.9 (3.9)	6.7 (2.2)	7.3 (2.4)	5.6 (1)	5.5 (1)	5.1 (1)	4.7 (0.3)
			UCLUST	0.5 (0.4)	0.7 (0.7)	0.7 (1.1)	0.3 (0.2)	0.3 (0.2)	0.1 (0.1)	0.1 (0.1)	0.1 (0.2)	0.1 (<0.1)	0.3 (0.3)
			SortMeRNA	0.9 (1.9)	2.1 (4.8)	1.8 (1.8)	2 (1.7)	2 (1.5)	3.8 (4.2)	2.2 (1)	2.9 (3.4)	2.7 (1.5)	2.3 (0.5)
			Kallisto	422.7 (354.9)	765 (324.1)	875 (270)	934.6 (272.6)	1001 (133.8)	991.9 (146.7)	953.9 (165.3)	910.8 (83.5)	875.5 (62.5)	860.7 (33.1)
			USEARCH61	0.4 (0.4)	0.8 (0.9)	0.7 (0.4)	0.8 (0.5)	0.7 (0.5)	0.5 (0.2)	0.6 (0.6)	0.8 (1)	0.6 (0.5)	0.5 (0.4)
			Mothur NB	NA (NA)	NA (NA)	NA (NA)	NA (NA)	NA (NA)	NA (NA)	NA (NA)	NA (NA)	NA (NA)	NA (NA)
			16S Classifier	1253.5 (573.7)	1152.9 (532.1)	1245.4 (311.4)	1082.1 (302.1)	1056.5 (189.9)	1007.4 (142.9)	964.2 (74.9)	876.1 (95.4)	933.3 (67.4)	908.8 (13.2)
			SINTAX	<0.1 (<0.1)	0.2 (1.1)	0.1 (0.3)	0.1 (0.2)	0.6 (1.6)	0.2 (0.2)	0.3 (0.4)	0.5 (1.2)	0.7 (0.6)	0.5 (0.4)
	V4	190	Karp	10.1 (49.9)	6 (11.3)	8.4 (8.9)	29.1 (49.1)	8.1 (7.7)	6.2 (4.1)	5.2 (1.7)	6.1 (3.5)	5 (1.6)	3.8 (0.8)
			Karp-Collapse	0.3 (1.7)	0.3 (0.8)	0.3 (0.5)	5.7 (17.3)	0.3 (0.3)	0.3 (0.2)	0.3 (0.2)	0.3 (0.1)	0.3 (0.2)	0.2 (0.1)
			UCLUST	<0.1 (<0.1)	<0.1 (<0.1)	<0.1 (<0.1)	<0.1 (<0.1)	<0.1 (<0.1)	<0.1 (<0.1)	<0.1 (<0.1)	<0.1 (<0.1)	<0.1 (<0.1)	0.2 (0.6)
			SortMeRNA	0.1 (<0.1)	0.1 (<0.1)	0.1 (<0.1)	0.1 (<0.1)	0.2 (<0.1)	0.2 (0.1)	0.3 (0.1)	0.4 (0.1)	0.4 (0.1)	0.7 (0.6)
			Kallisto	433 (357.9)	815.4 (401.5)	922.9 (303.1)	1101.6 (207.4)	1167.7 (216.8)	1148.7 (133.4)	1155.1 (167.4)	1121.2 (111.8)	1066.4 (95.6)	1107.7 (39.1)
			USEARCH61	<0.1 (<0.1)	<0.1 (<0.1)	<0.1 (<0.1)	<0.1 (<0.1)	<0.1 (<0.1)	<0.1 (<0.1)	<0.1 (<0.1)	<0.1 (<0.1)	<0.1 (<0.1)	0.2 (0.6)
			Mothur NB	354.1 (485)	222.7 (108.1)	227.1 (182.4)	201.7 (79.5)	140.5 (41.1)	152 (17)	136.7 (33.8)	109.8 (15.9)	91.1 (17.5)	51.2 (13.5)
			16S Classifier	171.8 (510)	71.9 (122.7)	97.2 (214.6)	141.2 (195.1)	102.9 (64.3)	115.9 (63.8)	132.9 (67.9)	165 (37.9)	151 (46.6)	144.4 (13)
			SINTAX	<0.1 (<0.1)	<0.1 (<0.1)	<0.1 (<0.1)	<0.1 (<0.1)	<0.1 (<0.1)	<0.1 (<0.1)	<0.1 (<0.1)	0.7 (1.8)	0.1 (0.2)	0.2 (0.6)

SE=Single-end, PE=Paired-end

Table 13: **Per species error rates, 100,000 reads**

Read Length	Region	Samples	Method	Shannon Diversity									
				[0,0.7)	[0.7,1.4)	[1.4,2)	[2,2.7)	[2.7,3.4)	[3.4,4.1)	[4.1,4.8)	[4.8,5.5)	[5.5,6.2)	[6.2,6.9]
75bp SE	Full gene	130	Karp	1.6 (2.5)	2.1 (3.4)	4.1 (4.8)	4.1 (3.1)	4.8 (2.7)	4.3 (1.3)	4.4 (0.9)	4.2 (0.9)	3.7 (0.7)	3.4 (0.3)
			Karp-Collapse	2.9 (4.4)	3.8 (7.1)	6.3 (6.4)	8.8 (6.2)	9.2 (5.2)	8.7 (2.6)	8.6 (1.4)	8.6 (1.2)	6.7 (1)	5.5 (0.3)
			UCLUST	2.9 (6)	5.3 (11.9)	7.4 (9.3)	7.8 (6.7)	9.8 (6.3)	7.8 (2.9)	8.2 (2.2)	8.1 (1.5)	6.5 (1.1)	5.9 (0.4)
			SortMeRNA	4.1 (10.5)	5.6 (12.6)	7.7 (9.2)	9.4 (8)	11 (7.2)	9.2 (3.6)	9.9 (3.2)	9.7 (1.5)	8 (1.3)	7 (0.4)
			Kallisto	2.4 (3.4)	2.9 (5.1)	5.5 (6)	5.9 (4.2)	6.8 (4)	5.9 (1.7)	5.9 (1.4)	5.9 (1)	5.1 (0.9)	4.7 (0.3)
			USEARCH61	3.5 (8.2)	6.1 (14.5)	7.5 (8.9)	8.5 (7.3)	10.1 (6.3)	8 (2.6)	8.6 (2.5)	8.7 (1.5)	7 (1.4)	6.1 (0.4)
			Mothur NB	5.6 (14.4)	8.1 (18.6)	12.5 (17.8)	13.3 (9.5)	17.5 (15.4)	14.6 (8)	14 (3.5)	14.5 (1.1)	12.8 (1.6)	12 (0.9)
			16S Classifier	7.9 (24.2)	12.4 (25.7)	12.5 (20.3)	11.9 (10.4)	12.4 (11.4)	10.5 (7.2)	11.1 (4.7)	11.2 (1.3)	10.3 (1.4)	10.8 (0.7)
			SINTAX	0.9 (2.5)	1.5 (3.5)	4.8 (10.5)	3.5 (5.2)	6.2 (4.1)	5.0 (2.5)	5.9 (3.0)	6.7 (1.7)	6.0 (1.1)	6.6 (0.6)
	V4	130	Karp	0.9 (1.6)	1.5 (2.3)	2.2 (2.1)	2 (1.9)	2.6 (1.7)	2.6 (1.4)	2.1 (0.7)	2.3 (0.6)	2 (0.3)	1.9 (0.3)
			Karp-Collapse	2 (3.6)	3.6 (4.5)	4 (4)	4.5 (4.8)	5.3 (3.4)	5.7 (2.6)	6.1 (1.4)	5.2 (1.2)	5 (0.6)	4.1 (0.4)
			UCLUST	2.2 (4.2)	2.9 (3.6)	4.5 (5.1)	4.9 (5.9)	5.3 (4.1)	5.1 (2.4)	5 (1.4)	5.1 (1.8)	4.4 (0.6)	4.2 (0.4)
			SortMeRNA	1.8 (3.1)	3 (3.9)	4.6 (5.5)	4.5 (4.7)	5.5 (4)	5.5 (2.6)	5.4 (1.2)	5.6 (1.6)	5.3 (0.8)	4.9 (0.5)
			Kallisto	1.6 (2.8)	2.4 (3.2)	3.3 (3.3)	3.1 (3)	3.9 (2.5)	3.5 (1.8)	3.2 (0.9)	3.7 (1.1)	3.1 (0.5)	3.1 (0.4)
			USEARCH61	2.8 (6.2)	3 (3.9)	4.7 (5.5)	4.6 (5.4)	5.2 (3.8)	4.9 (2.2)	4.7 (1.2)	5.1 (1.7)	4.6 (0.8)	4.1 (0.5)
			Mothur NB	8.9 (19.3)	7.6 (13)	8.6 (11.3)	9.6 (10.9)	8 (4.7)	9.4 (5)	9.8 (3.1)	9.6 (2.4)	9.3 (1)	9.4 (0.8)
			16S Classifier	14.5 (33)	12.2 (21)	10.9 (18.7)	8.2 (10.9)	6.9 (5.5)	7.3 (5)	7.7 (2.6)	7.6 (2.2)	7.1 (0.9)	7.9 (0.7)
			SINTAX	0.5 (0.6)	1.3 (2.2)	2.7 (5.3)	5.7 (7.5)	4.6 (5.7)	3.9 (2.7)	4.2 (1.5)	5.2 (2.4)	4.4 (0.7)	4.9 (0.6)
151bp PE	Full gene	130	Karp	0.1 (0.4)	0.1 (0.1)	0.7 (1.5)	0.1 (0.1)	0.4 (0.3)	0.5 (0.3)	0.5 (0.3)	0.4 (0.1)	0.5 (0.1)	0.4 (<0.1)
			Karp-Collapse	0.3 (0.4)	0.2 (0.4)	1.4 (3.5)	1.1 (1.3)	1.9 (1.6)	2 (1.1)	2.7 (1.8)	2.3 (0.7)	1.9 (0.5)	1.2 (0.2)
			UCLUST	0.1 (0.1)	0.3 (0.5)	3.3 (11.8)	1.2 (1.3)	3.5 (4.1)	2.3 (0.9)	3.5 (1.4)	2.5 (0.8)	3 (0.7)	2.3 (0.3)
			SortMeRNA	0.3 (0.3)	0.5 (0.9)	3.3 (9.9)	2 (2.1)	3 (1.5)	4 (2)	4.4 (2)	4.1 (1.2)	4.3 (0.9)	3.3 (0.3)
			Kallisto	0.2 (0.6)	<0.1 (0.1)	0.2 (0.7)	0.3 (0.4)	0.5 (0.5)	0.4 (0.3)	0.6 (0.5)	0.6 (0.2)	0.8 (0.2)	0.8 (0.1)
			USEARCH61	0.2 (0.2)	0.2 (0.3)	5.4 (18.3)	1.1 (1.7)	2.8 (2.1)	3 (1.6)	3.4 (2)	2.2 (0.6)	2.9 (0.8)	2.5 (0.3)
			Mothur NB	3.3 (5.4)	2.9 (5.2)	6.4 (8.3)	8.9 (8.1)	13.9 (6.7)	10.4 (4)	11.5 (3.7)	12.5 (2.2)	13.6 (1.6)	12.5 (0.5)
			16S Classifier	10.3 (24.3)	2.5 (4.5)	8.7 (15.8)	7.6 (9.8)	13.5 (9.5)	8.1 (4.9)	8.2 (3.2)	9.9 (2)	11.4 (1.9)	10.7 (0.7)
			SINTAX	0.1 (0.1)	0.3 (0.5)	4.9 (18.5)	0.9 (1.4)	4.0 (3.7)	3.0 (2.2)	3.5 (2.4)	3.5 (1.0)	4.2 (1.1)	3.4 (0.4)
	V4	190	Karp	<0.1 (<0.1)	<0.1 (<0.1)	<0.1 (<0.1)	<0.1 (<0.1)	<0.1 (<0.1)	0.3 (0.7)	0.1 (0.2)	0.1 (<0.1)	0.1 (<0.1)	0.2 (0.1)
			Karp-Collapse	<0.1 (<0.1)	<0.1 (0.1)	<0.1 (<0.1)	<0.1 (<0.1)	<0.1 (<0.1)	0.2 (0.4)	0.1 (0.2)	0.1 (0.2)	0.1 (0.1)	0.1 (0.1)
			UCLUST	7.2 (33.9)	0.2 (0.9)	<0.1 (0.1)	3.7 (10.6)	0.1 (0.2)	0.6 (1.1)	0.5 (0.6)	0.5 (0.7)	0.4 (0.4)	0.6 (0.4)
			SortMeRNA	0.4 (1.4)	1.6 (6.9)	0.7 (2)	6.4 (18.8)	0.3 (0.4)	1.3 (1.3)	1.1 (1.3)	2.1 (1.3)	1.1 (0.6)	1.2 (0.6)
			Kallisto	<0.1 (0.1)	0.1 (0.2)	0.1 (0.1)	0.3 (0.6)	0.4 (0.7)	0.9 (1.5)	0.8 (0.5)	0.8 (0.7)	0.7 (0.1)	0.8 (0.2)
			USEARCH61	7.7 (33.8)	0.2 (0.9)	0.3 (1)	3.7 (10.7)	1.8 (4.9)	0.7 (1.6)	1.3 (1.6)	0.9 (0.9)	0.7 (0.4)	0.9 (0.5)
			Mothur NB	3.5 (10.7)	11.4 (13.1)	6 (8.9)	9.8 (11.7)	10.5 (5.9)	10 (5.3)	9.4 (2.9)	9.7 (2.5)	10.4 (0.9)	10.3 (0.8)
			16S Classifier	23.1 (7.4)	22.7 (19.8)	12.3 (8.1)	9.8 (8.3)	9.2 (9.4)	6 (8.8)	2.9 (7.1)	2.1 (6.8)	0.7 (7.7)	0.8 (8.2)
			SINTAX	0.1 (0.2)	3.7 (11.1)	7.4 (23.1)	3.7 (8.6)	1.1 (1.7)	4.9 (7.0)	2.5 (2.2)	2.9 (2.6)	2.5 (1.3)	2.9 (0.9)

SE=Single-end, PE=Paired-end

Table 14: **Per species error rates, 100,000 reads**

Read Length	Region	Samples	Method	Shannon Diversity									
				[0,0.7)	[0.7,1.4)	[1.4,2)	[2,2.7)	[2.7,3.4)	[3.4,4.1)	[4.1,4.8)	[4.8,5.5)	[5.5,6.2)	[6.2,6.9]
301bp PE	Full gene	170	Karp	0.1 (0.2)	0.5 (1.2)	0.4 (0.7)	<0.1 (0.1)	0.5 (0.5)	0.6 (0.6)	0.8 (0.4)	0.7 (0.3)	0.7 (0.3)	0.7 (0.1)
			Karp-Collapse	0.2 (0.3)	0.6 (1.4)	2 (3.7)	0.1 (0.3)	0.7 (0.7)	1 (0.7)	1.4 (1.1)	1.4 (0.9)	0.9 (0.4)	0.7 (0.2)
			UCLUST	0.5 (0.9)	1.8 (5.2)	1.6 (3.4)	0.3 (0.6)	0.7 (0.6)	2 (2.5)	1.7 (1.1)	1.7 (0.8)	1.8 (0.6)	1.6 (0.4)
			SortMeRNA	0.5 (0.8)	2 (4)	2.2 (3.7)	1.7 (1.6)	1.5 (1.5)	4.3 (5.4)	3 (1.2)	3.1 (1.1)	2.9 (0.9)	2.6 (0.4)
			Kallisto	0.8 (1.3)	5.2 (10.4)	1.4 (2)	4.1 (6.6)	6.1 (7.1)	10.7 (8.2)	11 (5.9)	11.2 (3.4)	12.7 (2.5)	13.1 (1.6)
			USEARCH61	0.4 (0.8)	1.2 (3.3)	1.6 (2.2)	0.5 (0.9)	1.1 (1.2)	1.7 (1.3)	1.8 (0.9)	2.1 (0.9)	2 (0.8)	1.6 (0.2)
			Mothur NB	3.6 (3.3)	10.7 (14.8)	10.9 (9.5)	5.2 (3.5)	10.4 (4)	12.6 (8)	13.9 (3.6)	12.9 (2)	13.9 (1.8)	12.9 (0.6)
			16S Classifier	20.4 (36.4)	16.8 (24.5)	12.3 (12.1)	4.6 (4.5)	8.4 (4.7)	9.1 (5.7)	11.7 (4.8)	10 (1.9)	11.7 (2.3)	11.2 (1)
			SINTAX	0.5 (1.0)	1.4 (3.3)	1.3 (3.3)	0.3 (0.7)	1.8 (2.1)	3.5 (3.8)	2.7 (1.4)	3.1 (0.9)	3.2 (1.0)	3.2 (0.4)
	V4	190	Karp	<0.1 (<0.1)	<0.1 (0.1)	0.1 (0.1)	0.1 (0.2)	0.5 (1.6)	0.1 (<0.1)	0.1 (0.1)	0.2 (0.1)	0.2 (0.1)	0.3 (0.1)
			Karp-Collapse	<0.1 (<0.1)	<0.1 (0.1)	<0.1 (<0.1)	0.1 (0.3)	0.4 (1.3)	<0.1 (<0.1)	0.1 (0.1)	0.1 (0.1)	<0.1 (<0.1)	0.1 (0.1)
			UCLUST	<0.1 (<0.1)	0.6 (3.2)	<0.1 (<0.1)	<0.1 (0.1)	0.1 (0.4)	0.2 (0.6)	0.2 (0.4)	0.5 (0.9)	0.3 (0.3)	0.7 (0.2)
			SortMeRNA	<0.1 (<0.1)	0.1 (0.2)	<0.1 (0.1)	0.3 (0.5)	1.2 (3.5)	0.8 (1.2)	0.8 (0.8)	0.9 (0.8)	0.6 (0.6)	0.6 (0.3)
			Kallisto	0.1 (0.2)	3.3 (7.7)	2.4 (4.1)	5.3 (10.2)	3 (2.6)	5.6 (2.6)	7.6 (4.8)	9.4 (2.6)	9 (2.6)	11 (1.4)
			USEARCH61	<0.1 (<0.1)	<0.1 (0.1)	0.1 (0.4)	0.1 (0.4)	1.7 (5.2)	0.6 (0.8)	0.7 (1.1)	0.5 (0.3)	0.6 (0.4)	0.7 (0.3)
			Mothur NB	2 (4)	3.3 (5.1)	7.2 (9.9)	5.5 (5.3)	6.3 (5.9)	9.1 (5.1)	9.2 (3.5)	10.9 (2.4)	9.4 (1.9)	10 (0.8)
			16S Classifier	14.9 (33.5)	5.6 (9.3)	12 (18.9)	6.8 (9.2)	4.9 (4.6)	7.1 (4.6)	7 (3.5)	7.9 (2)	7.1 (1.8)	8 (1)
			SINTAX	<0.1 (0.2)	<0.1 (<0.1)	4.9 (11.9)	0.3 (0.7)	0.5 (1.0)	3.6 (5.4)	3.0 (2.3)	2.5 (1.3)	2.3 (1.0)	2.9 (0.6)

SE=Single-end, PE=Paired-end

Table 15: **Per genus error rates, 100,000 reads**

Read Length	Region	Samples	Method	Shannon Diversity									
				[0,0.7)	[0.7,1.4)	[1.4,2)	[2,2.7)	[2.7,3.4)	[3.4,4.1)	[4.1,4.8)	[4.8,5.5)	[5.5,6.2)	[6.2,6.9]
75bp SE	Full gene	130	Karp	8.8 (9.7)	11.6 (7.5)	14.2 (5.5)	14.8 (4.2)	13.8 (2)	14 (1.8)	12.9 (1.2)	10.8 (0.8)	10.1 (1)	8.3 (0.4)
			Karp-Collapse	17.3 (18)	20.2 (13.3)	27.7 (9.9)	29.7 (6.1)	27.6 (3.2)	26.6 (2.2)	24.3 (2.7)	20.9 (1.6)	18.2 (1.2)	13.8 (0.4)
			UCLUST	15.3 (17.5)	20.1 (15.3)	26.5 (10.1)	27.3 (6.5)	26.4 (3.6)	25.9 (3.2)	24.2 (3)	19.9 (1.4)	18.1 (1.4)	13.8 (0.5)
			SortMeRNA	16.7 (19)	21.3 (15.5)	30.5 (10.9)	32 (8.3)	31.2 (4.9)	30.5 (3.5)	29 (3.2)	25 (1.7)	22.7 (2.1)	17.2 (0.7)
			Kallisto	14.4 (15.1)	16.9 (11)	21.6 (7.8)	22.2 (5.4)	21.2 (3.2)	20.1 (2.4)	18.6 (1.8)	15.8 (1.2)	14.4 (1.4)	11.7 (0.5)
			USEARCH61	16.3 (19)	20.7 (15.3)	28.2 (10.7)	28.3 (6.6)	27.5 (3.6)	26.8 (2.4)	25 (2.6)	20.9 (1.4)	18.8 (1.5)	14.2 (0.5)
			Mothur NB	20.6 (30.4)	22.1 (18.2)	43.6 (25.6)	37.4 (15.2)	40.8 (11)	39.7 (4.5)	37.6 (6.3)	36.6 (2.3)	34 (1.7)	31.8 (1.9)
			16S Classifier	89.3 (93.6)	86.4 (77.6)	113 (49.8)	99.4 (47.6)	90.9 (28.9)	89.1 (24.7)	86.1 (12.6)	89 (5.6)	86.4 (6.3)	86.5 (2.5)
			SINTAX	5.2 (5.6)	12.7 (15.0)	19.0 (13.4)	19.4 (7.6)	18.2 (6.7)	22.0 (6.5)	20.3 (3.1)	19.3 (1.6)	19.7 (2.0)	19.2 (0.7)
	V4	130	Karp	4.7 (6)	8.1 (8.7)	10.1 (8.3)	10.3 (3.8)	9.2 (4.1)	8.6 (2.8)	6.8 (1.6)	7.3 (1.2)	6.5 (0.9)	5.6 (0.5)
			Karp-Collapse	11.3 (11.3)	16.7 (13.7)	21.6 (14)	20.9 (6.7)	19.3 (6.2)	20.3 (5)	16.1 (3.2)	15.2 (1.7)	13.8 (1.6)	10.4 (0.8)
			UCLUST	7.7 (7.9)	15 (17.8)	19.6 (16.4)	19.5 (8.6)	17.6 (6.5)	16.9 (4.7)	13.4 (3.6)	13.9 (1.7)	12 (1.7)	9.8 (0.8)
			SortMeRNA	7.1 (7.9)	16.8 (20)	20.6 (15.1)	19.1 (7.7)	19.4 (6.7)	19.9 (4.6)	16.1 (3.3)	16.9 (1.5)	15.9 (1.7)	12.7 (0.7)
			Kallisto	9.4 (9.4)	13.6 (12.2)	18.7 (14.5)	16.5 (6.3)	15 (4.5)	15 (4.1)	12.2 (2.3)	12.5 (2.1)	11 (1.3)	9.3 (0.7)
			USEARCH61	8.7 (11.2)	17.2 (21.4)	20.9 (16.4)	19 (9.3)	18.6 (6.3)	17.5 (4.1)	14 (3.1)	14.1 (1.6)	12.7 (1.8)	10.1 (0.8)
			Mothur NB	11.5 (10.7)	25.7 (25.6)	34.1 (22.4)	39.2 (20.6)	36.8 (9.5)	35.4 (8.4)	32.2 (5.5)	33.2 (3.1)	30.7 (2.5)	28.7 (0.9)
			16S Classifier	79.2 (79.9)	86.7 (63)	77.2 (48.1)	80.6 (33.6)	80.9 (14.7)	76.2 (19.7)	68 (14.8)	72.8 (8.5)	69 (5.1)	70.2 (2.3)
			SINTAX	4.4 (4.5)	11.5 (17.8)	18.8 (21.6)	17.4 (13.5)	19.0 (8.4)	18.3 (4.9)	16.1 (4.5)	18.6 (3.2)	17.3 (2.7)	17.3 (0.8)
151bp PE	Full gene	130	Karp	0.5 (1.4)	0.5 (0.6)	0.7 (0.9)	1 (0.7)	1.1 (0.6)	0.8 (0.3)	1.2 (0.3)	1.1 (0.3)	1.1 (0.2)	1.1 (0.1)
			Karp-Collapse	1.4 (4)	2.3 (4.3)	2.1 (3)	4.2 (4.7)	3 (2.4)	2.4 (1.8)	4.5 (3.1)	2.6 (0.8)	2 (0.5)	1.8 (0.2)
			UCLUST	1 (2)	1.5 (1.7)	5.1 (11.5)	2.9 (2.2)	3.8 (2.3)	3.9 (2)	6.2 (2.5)	4 (0.7)	3.8 (0.7)	3.8 (0.5)
			SortMeRNA	2.7 (4.3)	3 (3.8)	7.1 (9.6)	7.1 (4.3)	5.9 (2.5)	6 (2.4)	8.3 (2.8)	6.7 (1.2)	6.4 (0.7)	5.6 (0.3)
			Kallisto	0.9 (1.1)	1.9 (2)	1.6 (1.2)	2.7 (1.4)	2.7 (1)	3.1 (0.8)	3.3 (0.6)	3.3 (0.3)	3.4 (0.3)	3.6 (0.1)
			USEARCH61	2.1 (5.3)	1.7 (1.6)	3.8 (5)	6.5 (6.4)	4.6 (4)	4.4 (2.8)	6 (2.5)	4.6 (0.9)	4.2 (0.8)	4.2 (0.4)
			Mothur NB	11.4 (20.5)	19.8 (17.4)	26.3 (10.3)	31.1 (7.6)	29.3 (6.6)	29.4 (5.2)	29.6 (2.6)	27.7 (3)	27.5 (1.2)	24.7 (1.1)
			16S Classifier	84.3 (85.7)	83.7 (67.3)	62.9 (38.2)	71.1 (18.5)	77.6 (28.2)	79.4 (10.9)	73.6 (7.9)	77.3 (9.9)	77.1 (7)	75.4 (3.1)
			SINTAX	1.3 (2.5)	2.5 (3.9)	6.7 (17.0)	5.5 (3.6)	4.9 (2.0)	6.1 (2.8)	7.5 (3.3)	6.4 (1.4)	6.0 (1.4)	5.7 (0.7)
	V4	190	Karp	<0.1 (<0.1)	0.1 (0.3)	0.3 (1)	0.1 (<0.1)	0.4 (0.6)	0.3 (0.1)	0.5 (0.5)	0.4 (0.2)	0.5 (0.2)	0.6 (0.1)
			Karp-Collapse	<0.1 (0.1)	0.1 (0.3)	0.3 (1)	<0.1 (<0.1)	0.3 (0.6)	0.1 (0.2)	0.4 (0.5)	0.2 (0.3)	0.2 (0.2)	0.2 (0.1)
			UCLUST	4 (25)	0.4 (1.6)	0.8 (2.9)	3.6 (10.1)	0.6 (1.1)	0.4 (0.6)	1.5 (3.4)	0.9 (0.5)	1.1 (0.8)	1.5 (0.5)
			SortMeRNA	2.3 (11)	6.6 (21)	2.3 (8.2)	6.5 (17.6)	1.5 (1.6)	1.6 (1.6)	3.4 (4.2)	3.4 (2.4)	2.9 (1.5)	3 (0.8)
			Kallisto	0.6 (0.8)	1.5 (1.8)	2.8 (2.8)	2.7 (0.8)	3.6 (1.8)	3.5 (0.9)	3.6 (0.6)	3.9 (0.9)	4 (0.4)	3.9 (0.3)
			USEARCH61	4.5 (24.8)	0.8 (2.5)	0.7 (2.8)	4.9 (10.3)	1 (1.5)	0.8 (1)	3.5 (4.4)	1.8 (1.1)	2.4 (1.3)	2.4 (0.5)
			Mothur NB	14.7 (11.2)	24.3 (10.4)	25.8 (17.3)	30.9 (10.5)	29.9 (6.7)	28.1 (6.2)	28.1 (3.1)	28.9 (2.3)	27.4 (2.4)	24.9 (1.1)
			16S Classifier	39.1 (70.6)	55.6 (65.4)	37.6 (33.4)	26.6 (21.1)	33.2 (24.4)	32.4 (9.1)	30 (9.2)	34.2 (8.3)	31.1 (2.7)	33.2 (3.1)
			SINTAX	7.7 (34.0)	8.0 (21.7)	11.0 (24.7)	4.5 (9.4)	2.0 (2.4)	3.7 (2.5)	5.3 (5.4)	3.9 (2.2)	5.3 (1.3)	4.2 (1.0)

SE=Single-end, PE=Paired-end

Table 16: **Per genus error rates, 100,000 reads**

Read Length	Region	Samples	Method	Shannon Diversity									
				[0,0.7)	[0.7,1.4)	[1.4,2)	[2,2.7)	[2.7,3.4)	[3.4,4.1)	[4.1,4.8)	[4.8,5.5)	[5.5,6.2)	[6.2,6.9]
301bp PE	Full gene	170	Karp	0.2 (0.4)	0.8 (1.8)	0.3 (0.3)	1 (2.1)	0.9 (0.7)	1.4 (1)	1.6 (0.8)	1.4 (0.3)	1.5 (0.3)	1.7 (0.1)
			Karp-Collapse	0.2 (0.5)	0.7 (1.3)	1.9 (4.1)	1.4 (3.2)	1.4 (1.5)	1.4 (0.9)	1.8 (1.4)	1.4 (0.8)	1.3 (0.3)	0.9 (0.2)
			UCLUST	0.3 (0.4)	1.5 (3.4)	2.8 (6.1)	2.9 (5.2)	1.7 (1.3)	3.7 (3.3)	3.1 (2.5)	2.2 (0.7)	2.1 (0.5)	2 (0.3)
			SortMeRNA	0.9 (1.5)	2.9 (3.8)	4.8 (6.2)	4 (4.4)	5.5 (3)	7.8 (6.1)	6.1 (1.6)	4.8 (1.2)	4.4 (0.7)	4 (0.3)
			Kallisto	15.5 (27.7)	26.5 (34.9)	28.1 (23.7)	34.9 (26.1)	37.1 (14.8)	47.2 (9.2)	51.2 (9.2)	51.4 (6)	53.7 (6)	52.1 (2.2)
			USEARCH61	0.2 (0.4)	1.8 (4.9)	1.7 (2.2)	2.4 (5.3)	2.3 (2.4)	2.4 (1.6)	2.8 (1.6)	2.6 (0.7)	2.1 (0.5)	2.1 (0.3)
			Mothur NB	9.7 (20)	25.8 (24.2)	26.9 (18.3)	25.4 (12.1)	30.9 (4.6)	31.1 (9.8)	30.2 (3.6)	28.8 (2.6)	28.2 (1.4)	26.2 (1.3)
			16S Classifier	62.5 (79.6)	51.3 (54.8)	77.7 (50.1)	60.1 (30.2)	67.2 (9.7)	71.6 (15.1)	70.2 (12.7)	65.9 (10.1)	67 (7.3)	67.2 (4)
			SINTAX	0.7 (1.1)	3.9 (8.7)	4.0 (6.8)	3.0 (3.3)	3.5 (3.5)	6.4 (4.8)	4.8 (2.6)	4.1 (1.0)	4.4 (0.9)	4.3 (0.7)
	V4	190	Karp	0.1 (0.2)	0.3 (0.5)	0.6 (1)	0.8 (1)	1.3 (2.8)	0.6 (0.2)	0.6 (0.1)	0.7 (0.2)	1 (0.2)	1.1 (0.1)
			Karp-Collapse	<0.1 (<0.1)	0.1 (0.4)	<0.1 (0.1)	0.2 (0.4)	<0.1 (0.1)	0.2 (0.3)	0.1 (0.1)	0.1 (0.1)	0.1 (<0.1)	0.1 (<0.1)
			UCLUST	<0.1 (<0.1)	<0.1 (0.1)	0.6 (2.5)	<0.1 (<0.1)	<0.1 (0.1)	0.4 (0.8)	0.3 (0.4)	0.9 (0.9)	0.4 (0.3)	0.7 (0.4)
			SortMeRNA	0.1 (0.5)	0.7 (2.4)	0.4 (2)	0.8 (1.6)	0.8 (1.5)	2 (2.1)	1.7 (1.8)	2.3 (1.3)	1.2 (0.6)	1 (0.3)
			Kallisto	14.6 (28.4)	38.1 (43)	37.7 (24)	41.4 (22.5)	41.7 (16.7)	52.6 (17.7)	47.9 (10.9)	55.7 (7.3)	55.6 (6.9)	57.5 (2.7)
			USEARCH61	<0.1 (0.2)	0.2 (0.8)	<0.1 (<0.1)	0.2 (0.5)	0.1 (0.3)	1 (1.2)	1 (1.3)	0.9 (0.7)	1 (0.7)	0.9 (0.3)
			Mothur NB	28.1 (55)	27.3 (33.2)	29.3 (12.6)	28 (10.5)	29.7 (5)	30 (3.5)	27.3 (3.6)	29.7 (3)	26.8 (2.2)	24.7 (1.1)
			16S Classifier	38.4 (74.8)	30.4 (42.4)	52.5 (44.8)	25 (20.9)	32.2 (20.6)	32.6 (12.2)	32.9 (10.1)	31.2 (7.8)	32.2 (5.6)	31.6 (2.4)
			SINTAX	0.5 (1.3)	4.2 (14.6)	5.1 (17.2)	0.7 (1.1)	1.8 (4.9)	5.9 (4.8)	3.4 (2.0)	4.3 (2.8)	4.0 (2.1)	4.1 (0.8)

SE=Single-end, PE=Paired-end

Table 17: **Per family error rates, 100,000 reads**

Read Length	Region	Samples	Method	Shannon Diversity									
				[0,0.7)	[0.7,1.4)	[1.4,2)	[2,2.7)	[2.7,3.4)	[3.4,4.1)	[4.1,4.8)	[4.8,5.5)	[5.5,6.2)	[6.2,6.9]
75bp SE	Full gene	130	Karp	15 (8.3)	16.8 (8.4)	15.9 (6.3)	16.6 (3.8)	15.7 (3)	13.9 (2.2)	13.1 (2)	10 (0.7)	8.7 (1)	6.4 (0.3)
			Karp-Collapse	34.6 (15.2)	31 (13.3)	33 (9.5)	36.2 (5.9)	32.3 (6.5)	26.9 (3)	25 (3.7)	18.9 (1.2)	15.4 (1.7)	10.7 (0.5)
			UCLUST	28.6 (13.5)	27.7 (13)	28.9 (8.8)	30 (6.2)	28.6 (6.2)	23.9 (3)	22.3 (3.1)	17 (1.4)	13.6 (1.7)	9.2 (0.5)
			SortMeRNA	29.7 (14.3)	29.1 (13.2)	32.9 (10.6)	34.9 (5.5)	34.5 (7.1)	30.1 (3)	28.9 (3.7)	22.3 (1.5)	18.4 (1.9)	12.8 (0.5)
			Kallisto	27.2 (12.8)	26.3 (11.6)	26.9 (8.2)	27.6 (5)	25.7 (3.5)	21.8 (2.4)	20.5 (2.7)	15.8 (1.3)	13.2 (1.3)	9.5 (0.3)
			USEARCH61	30.7 (14.6)	28.6 (13.7)	30.5 (10)	31 (5.9)	29.7 (6.3)	24.7 (2.6)	23.4 (3.6)	17.8 (1.4)	14.2 (1.9)	9.6 (0.5)
			Mothur NB	45.3 (56.4)	34.1 (21.2)	39.1 (21.8)	38 (11.1)	45.5 (9.6)	40.2 (7.4)	39.3 (4.2)	37.4 (3.2)	35.4 (2.1)	34.2 (1.8)
			16S Classifier	173.5 (57.9)	131.8 (71.1)	151.9 (40.2)	172 (19.3)	147.3 (10.6)	156.1 (12.4)	149.8 (10.7)	144.3 (6.9)	146.4 (4.6)	145.7 (1.6)
			SINTAX	7.7 (4.1)	16.4 (10.6)	17.2 (9.4)	21.7 (4.7)	22.6 (7.3)	21.8 (3.3)	21.4 (2.7)	18.6 (1.5)	18.8 (1.7)	17.8 (0.7)
	V4	130	Karp	6.1 (7.9)	8.6 (7.8)	9 (3.9)	7.5 (3.9)	8.7 (4.2)	8.1 (2.1)	7 (1.1)	6.1 (0.9)	5.7 (0.6)	4.5 (0.3)
			Karp-Collapse	17.8 (13.4)	20 (12.8)	21 (7.3)	18.9 (8.1)	20.1 (6)	18.6 (5.9)	16 (2.9)	12.8 (1.7)	11.1 (0.9)	7.9 (0.8)
			UCLUST	9.3 (11)	15.4 (13.7)	16.6 (7.9)	14.1 (7.9)	14.5 (5.9)	14.6 (6.7)	11.6 (2.3)	9.5 (2)	8.5 (1)	6.1 (0.7)
			SortMeRNA	9.3 (10.2)	15.6 (15.2)	17.1 (7.2)	14.7 (7.6)	17.4 (6.1)	18 (5.9)	16.2 (3)	13.9 (1.9)	13.7 (0.7)	10.5 (0.8)
			Kallisto	15.3 (12.1)	17.4 (12)	20 (10.3)	16.6 (6.3)	17.3 (5.2)	17.2 (5.2)	14.8 (1.9)	12 (1.1)	10.9 (0.6)	8.6 (0.4)
			USEARCH61	11.6 (15)	17.9 (17.4)	17.2 (7)	14.9 (8.2)	14.8 (5.8)	14.7 (5.6)	12.6 (2.5)	9.9 (1.8)	9 (0.7)	6.3 (0.6)
			Mothur NB	25.9 (29)	38 (26.3)	38.2 (15.4)	44.6 (16)	36.4 (9.8)	48.3 (13.2)	39.7 (5)	39.7 (5)	36.9 (2.6)	35.5 (1.4)
			16S Classifier	136.5 (69.9)	129 (46.1)	121.5 (44.5)	130.7 (19.5)	117.7 (18.2)	125 (17.3)	114.7 (10.9)	115.6 (7.7)	113.2 (2.7)	112 (3.4)
			SINTAX	6.3 (6.3)	14.2 (12.8)	18.8 (10.9)	16.7 (8.7)	16.9 (4.9)	22.2 (7.2)	18.1 (2.7)	18.5 (2.8)	18.3 (1.0)	16.6 (0.9)
151bp PE	Full gene	130	Karp	0.5 (1.1)	0.8 (1.2)	1.1 (1.2)	1.4 (0.7)	1.4 (0.9)	0.9 (0.2)	1.4 (0.4)	1.1 (0.3)	1.2 (0.2)	1.1 (0.1)
			Karp-Collapse	0.6 (1.2)	1.7 (2.9)	3.8 (5.2)	2 (1.9)	2.2 (2.5)	1.2 (0.5)	2.1 (1)	1.3 (0.3)	1.5 (0.2)	1.3 (0.2)
			UCLUST	2 (5.7)	2 (2.5)	4.3 (5.6)	3.9 (4.6)	3.3 (2.9)	3.3 (1.4)	3.4 (1.8)	2.5 (0.5)	2.4 (0.5)	2.3 (0.3)
			SortMeRNA	2.3 (4.5)	3.3 (4.8)	5.5 (6)	6.5 (5.5)	4.1 (3.1)	4.3 (2.3)	4.4 (1.9)	4.5 (0.5)	4.1 (0.6)	3.9 (0.3)
			Kallisto	1.9 (2.2)	3.3 (2.4)	3.8 (1.7)	5.2 (1.8)	5.3 (1.1)	4.9 (1)	5.6 (1.1)	5.6 (0.5)	5.4 (0.4)	5.5 (0.2)
			USEARCH61	0.9 (1.2)	2.1 (2.8)	3.9 (4.9)	4.7 (3.5)	4 (3.3)	3.5 (1.5)	3.2 (0.8)	2.9 (0.4)	3 (0.3)	2.9 (0.2)
			Mothur NB	9.9 (14.1)	17.1 (10.7)	24.6 (17.6)	20.7 (10.8)	18.4 (8.5)	15.2 (5.4)	15.7 (3.7)	13.7 (2.3)	14.3 (1.9)	12.2 (1.3)
			16S Classifier	135.5 (71.3)	135.4 (53.5)	118.7 (39.8)	123.9 (18.3)	118.3 (18.9)	127 (10)	113.5 (13)	121.8 (9.6)	114.7 (7)	116.3 (3.1)
			SINTAX	2.1 (5.8)	2.2 (3.0)	4.7 (5.7)	4.5 (5.2)	3.7 (4.3)	3.3 (1.6)	3.7 (2.0)	2.7 (0.8)	2.3 (0.5)	2.2 (0.2)
	V4	190	Karp	0.1 (0.1)	0.1 (0.3)	0.5 (1.9)	0.2 (<0.1)	0.5 (0.5)	0.4 (0.1)	0.6 (0.5)	0.5 (0.2)	0.6 (0.2)	0.6 (0.1)
			Karp-Collapse	<0.1 (0.1)	0.1 (0.3)	0.4 (1.9)	<0.1 (<0.1)	0.3 (0.6)	0.3 (0.3)	0.4 (0.6)	0.2 (0.2)	0.3 (0.2)	0.2 (0.1)
			UCLUST	4.1 (25)	0.3 (1)	0.9 (3.7)	4.6 (10.2)	1.5 (1.8)	0.5 (0.8)	2.3 (4.1)	1.7 (1)	1.5 (0.8)	1.7 (0.5)
			SortMeRNA	2.2 (11.1)	0.4 (1)	1.3 (3.8)	7.6 (17.5)	1.6 (1.7)	1.6 (1.6)	4.4 (5)	3.1 (1.6)	2.9 (1)	3 (0.5)
			Kallisto	1.7 (1.6)	3.9 (2.7)	5.1 (3.2)	4.8 (1.2)	5.9 (1.3)	5.7 (1.1)	6.3 (0.9)	6.3 (0.6)	6.1 (0.7)	6.3 (0.2)
			USEARCH61	4.4 (24.8)	0.3 (1)	1 (3.7)	5.1 (10.1)	1.4 (1.8)	0.7 (0.9)	3.9 (5.1)	2.7 (1.3)	2.8 (1.1)	2.6 (0.5)
			Mothur NB	20.4 (10.5)	30.9 (25.5)	27.5 (9.8)	31.2 (11.8)	27.3 (5.2)	26 (6.2)	21.3 (5.6)	19.7 (2.9)	16.5 (2.6)	14.1 (1.3)
			16S Classifier	46.6 (18.2)	63.7 (48.9)	27.4 (26)	29.2 (30.5)	19 (24.2)	9.4 (31.3)	6.8 (27)	4 (29.1)	4.1 (27.3)	1.9 (26.9)
			SINTAX	0.3 (1.2)	0.2 (0.8)	0.1 (0.1)	3.2 (9.2)	1.1 (1.8)	0.3 (0.4)	2.9 (5.0)	1.2 (1.2)	1.8 (0.9)	1.4 (0.4)

SE=Single-end, PE=Paired-end

Table 18: **Per family error rates, 100,000 reads**

Read Length	Region	Samples	Method	Shannon Diversity									
				[0,0.7)	[0.7,1.4)	[1.4,2)	[2,2.7)	[2.7,3.4)	[3.4,4.1)	[4.1,4.8)	[4.8,5.5)	[5.5,6.2)	[6.2,6.9]
301bp PE	Full gene	170	Karp	0.2 (0.3)	0.7 (1.2)	0.5 (0.3)	0.7 (0.7)	1 (0.7)	1.3 (0.9)	1.6 (0.5)	1.3 (0.2)	1.4 (0.1)	1.6 (0.1)
			Karp-Collapse	0.1 (0.2)	0.4 (0.9)	0.2 (0.2)	0.5 (0.7)	1 (1.4)	0.6 (1)	1 (0.8)	0.6 (0.3)	0.5 (0.1)	0.5 (0.1)
			UCLUST	<0.1 (0.1)	0.2 (0.4)	1.5 (4.9)	1.6 (3.3)	1.7 (1.8)	1.6 (2)	1.5 (1.4)	0.7 (0.4)	1 (0.5)	0.7 (0.2)
			SortMeRNA	0.8 (1.7)	1.7 (4.2)	2.1 (4.8)	2.3 (3)	3.2 (2.4)	2.8 (1.8)	3.4 (2)	3 (1.1)	2.5 (0.4)	2.2 (0.3)
			Kallisto	28.2 (35.2)	55.7 (39.3)	73.3 (33.7)	71.9 (17.7)	71.2 (12.4)	84.9 (15.5)	83.7 (9.7)	82.1 (5.5)	81.4 (7)	79.2 (3)
			USEARCH61	<0.1 (0.1)	0.2 (0.2)	0.2 (0.4)	0.6 (1.1)	1.5 (2)	0.8 (1.1)	1.5 (1.1)	0.8 (0.4)	0.7 (0.2)	0.9 (0.2)
			Mothur NB	6.8 (13.2)	14.1 (12.5)	12 (9.8)	17.6 (11.4)	23.4 (8.1)	15.5 (6.9)	19.2 (4.9)	14.1 (3.8)	15.1 (1.9)	12.6 (1.1)
			16S Classifier	94.4 (76.4)	109.3 (60.1)	128.3 (30.1)	105.9 (33)	105.8 (16.5)	101.6 (15.4)	96.7 (5.4)	93.3 (7.4)	90.9 (3.8)	93.4 (2.4)
			SINTAX	0.1 (0.1)	0.4 (1.2)	1.5 (4.8)	2.8 (4.4)	2.6 (3.6)	2.2 (2.1)	2.0 (1.3)	1.4 (0.8)	1.5 (0.6)	1.2 (0.2)
	V4	190	Karp	0.3 (0.6)	0.6 (0.6)	0.9 (1.1)	1 (1)	1.7 (3)	0.8 (0.2)	0.9 (0.2)	1 (0.2)	1.2 (0.2)	1.4 (0.1)
			Karp-Collapse	<0.1 (<0.1)	<0.1 (0.1)	<0.1 (<0.1)	<0.1 (<0.1)	<0.1 (0.1)	0.1 (0.3)	<0.1 (<0.1)	0.1 (0.1)	<0.1 (<0.1)	0.1 (<0.1)
			UCLUST	<0.1 (<0.1)	<0.1 (0.1)	0.3 (2)	<0.1 (<0.1)	<0.1 (<0.1)	<0.1 (<0.1)	<0.1 (<0.1)	0.5 (1.6)	0.1 (0.1)	0.2 (0.2)
			SortMeRNA	<0.1 (<0.1)	0.5 (2.1)	<0.1 (0.1)	0.1 (0.1)	0.5 (1.4)	0.6 (1.1)	1.7 (1.8)	1.1 (1.1)	0.6 (0.2)	0.5 (0.2)
			Kallisto	43.8 (44.7)	72.1 (43.5)	73.5 (26.6)	84.2 (25.6)	90 (22.8)	92.7 (17.2)	88.5 (13.6)	103.5 (11.4)	98.7 (5.2)	99.1 (3.9)
			USEARCH61	3.3 (20.9)	0.1 (0.3)	<0.1 (<0.1)	<0.1 (<0.1)	<0.1 (<0.1)	0.5 (0.9)	<0.1 (0.1)	0.5 (0.5)	0.5 (0.6)	0.3 (0.2)
			Mothur NB	39.9 (61.6)	42.6 (46.9)	26.6 (9.8)	28.2 (8.8)	27.4 (8.3)	23.5 (4.6)	23.9 (6.3)	22 (4.4)	17.3 (2.6)	15 (1.4)
			16S Classifier	19.3 (52)	33.2 (52.9)	36.1 (39.2)	33.8 (22.5)	31.7 (19.2)	26.6 (14.7)	29.4 (5.1)	29.9 (6.5)	26.9 (3.1)	25.1 (1.9)
			SINTAX	<0.1 (<0.1)	6.2 (24.7)	0.4 (1.7)	0.8 (2.3)	1.5 (3.3)	0.5 (1.1)	0.7 (1.3)	1.1 (0.9)	1.4 (0.9)	1.1 (0.4)

SE=Single-end, PE=Paired-end

Table 19: **Per order error rates, 100,000 reads**

Read Length	Region	Samples	Method	Shannon Diversity									
				[0,0.7)	[0.7,1.4)	[1.4,2)	[2,2.7)	[2.7,3.4)	[3.4,4.1)	[4.1,4.8)	[4.8,5.5)	[5.5,6.2)	[6.2,6.9]
75bp SE	Full gene	130	Karp	15.4 (11.2)	13.1 (10.8)	10.2 (4.4)	11.2 (3.1)	12.1 (3.9)	8.7 (2.2)	8.4 (1.6)	5.4 (0.6)	5.2 (0.5)	4.1 (0.4)
			Karp-Collapse	36.1 (17.9)	29.4 (16.5)	23.4 (7.2)	26.1 (7.4)	25.6 (8.5)	18.5 (3.5)	17 (3)	11.2 (1)	9.9 (1)	7.4 (0.5)
			UCLUST	28.9 (21.3)	23.1 (17.8)	17.1 (7.4)	19.7 (7.4)	20.4 (8.2)	14.1 (3.3)	13.5 (2.8)	8.9 (1)	7.7 (0.9)	5.7 (0.3)
			SortMeRNA	32.1 (23.6)	26.6 (18.9)	22.1 (7.9)	24.4 (6.8)	27.4 (9.3)	20.4 (3.7)	20 (2.8)	13.6 (1.3)	12.8 (1)	10.1 (0.7)
			Kallisto	28.4 (15.3)	24.5 (14.2)	19.3 (6)	21.1 (5.1)	19.9 (5.2)	15.4 (2.9)	14.7 (2.5)	10 (0.9)	9.4 (0.7)	7.4 (0.3)
			USEARCH61	30.4 (22.6)	24.8 (19.3)	19 (8.3)	20.3 (7.1)	21.2 (8.3)	14.8 (3.3)	14.3 (3)	9.5 (1)	8.1 (0.9)	6.1 (0.4)
			Mothur NB	46 (52.8)	43.3 (28.7)	39.3 (20.9)	33.2 (13.1)	49.5 (10.8)	37.2 (4.6)	36.3 (4.8)	35.6 (2.3)	34.4 (2.5)	33.7 (1.5)
			16S Classifier	178.7 (24.8)	174 (16.1)	153.3 (18.7)	151.9 (26.1)	152.7 (10.7)	144.4 (8.1)	141.2 (13.7)	139 (7.5)	141.7 (3.2)	142.5 (1.7)
			SINTAX	12.3 (14.1)	20.0 (12.1)	15.1 (7.6)	19.5 (7.9)	24.2 (8.6)	20.9 (4.2)	22.3 (2.9)	20.0 (1.0)	20.1 (0.9)	20.6 (0.6)
	V4	130	Karp	3.8 (4.7)	5.4 (4.5)	5.5 (3.2)	5.5 (3.4)	5.9 (1.4)	5.7 (1.4)	4.6 (0.8)	4.3 (0.5)	3.8 (0.4)	3.3 (0.2)
			Karp-Collapse	16.8 (12.9)	14.9 (12.1)	16.1 (8.4)	12.5 (4.8)	14.4 (2.4)	13.8 (3.7)	10.4 (2)	9.2 (2.1)	7.1 (0.6)	5.2 (0.4)
			UCLUST	6.5 (9)	8.2 (7.9)	7.7 (5.8)	6.1 (4.9)	8.2 (2)	8.5 (4.4)	5.5 (1.2)	6 (1.4)	4.4 (0.6)	3.5 (0.3)
			SortMeRNA	7.4 (9.6)	8.4 (7.1)	10.5 (6)	9.7 (5)	12.3 (1.6)	13.5 (3.2)	11.5 (2.6)	11.4 (2.1)	10.3 (0.6)	9.2 (0.6)
			Kallisto	14 (11.7)	13.8 (10.6)	14.5 (7.4)	12.6 (4.9)	13.6 (1.9)	13.1 (3.9)	11.1 (1.5)	10.1 (0.8)	9.1 (0.7)	7.8 (0.4)
			USEARCH61	9 (14.2)	9 (9.5)	8.7 (6.8)	7.5 (5.8)	8.6 (1.9)	8.6 (3.9)	6.5 (1.8)	6.4 (1.6)	4.9 (0.5)	3.8 (0.4)
			Mothur NB	31.4 (31.7)	41 (39.3)	48.5 (25)	42.3 (15)	46.9 (13.2)	47.5 (6)	39.7 (6.2)	40.1 (6.1)	35.6 (2)	34.1 (1.2)
			16S Classifier	148.1 (61.4)	126.5 (44.3)	130.9 (37.7)	112.6 (29.9)	118.5 (12.5)	116 (14.4)	106 (17.6)	108.1 (12.9)	103.8 (4.9)	102.4 (2.8)
			SINTAX	6.7 (6.0)	14.2 (10.4)	14.9 (8.1)	14.5 (7.2)	19.4 (3.2)	18.9 (4.8)	17.7 (1.9)	18.4 (2.8)	17.1 (0.5)	16.6 (0.5)
151bp PE	Full gene	130	Karp	0.5 (1.1)	0.6 (1)	0.8 (0.7)	1.2 (0.8)	1.2 (0.9)	0.8 (0.4)	1.2 (0.3)	1 (0.2)	1 (0.1)	1 (0.1)
			Karp-Collapse	0.5 (1.1)	0.6 (1)	0.9 (1)	1.1 (0.8)	1.8 (2.5)	0.8 (0.5)	1.4 (0.6)	1 (0.3)	1 (0.2)	1 (0.1)
			UCLUST	0.4 (0.3)	1 (1)	1.3 (1.1)	2.8 (3.8)	2.3 (2.9)	1.6 (0.9)	1.8 (0.5)	1.6 (0.4)	1.5 (0.2)	1.5 (0.2)
			SortMeRNA	2.2 (4.2)	1.1 (0.8)	2.3 (1.9)	3.6 (4.2)	2.5 (2.9)	2.4 (1)	2.4 (0.4)	3.1 (0.6)	3 (0.4)	3.2 (0.3)
			Kallisto	2.2 (2.1)	3.9 (2.3)	5 (2.4)	6.1 (1.8)	5.8 (1.3)	5.7 (0.9)	6.6 (0.8)	6 (0.6)	6.1 (0.5)	6.4 (0.2)
			USEARCH61	1.4 (2.1)	1.4 (0.8)	2.2 (1.6)	3.7 (3.6)	3.2 (3.8)	2.1 (0.5)	2.4 (0.5)	2.1 (0.3)	2.2 (0.3)	2.1 (0.3)
			Mothur NB	6.3 (9.5)	8.3 (7.1)	13.7 (15.2)	10 (7.5)	11.2 (7.3)	8 (3.1)	6.5 (2.8)	6.9 (2.9)	6.1 (1.3)	5.4 (1.1)
			16S Classifier	145.4 (60.4)	122.3 (49.7)	111.6 (48.1)	103.4 (35.7)	111.8 (16.8)	110 (19.6)	100.7 (14.8)	105.4 (9.8)	96.7 (6.3)	99.8 (1.8)
			SINTAX	0.3 (0.5)	1.0 (1.8)	1.0 (1.4)	2.5 (4.2)	2.2 (4.5)	1.2 (1.1)	1.1 (0.4)	1.3 (0.8)	1.1 (0.3)	1.2 (0.3)
	V4	190	Karp	0.1 (<0.1)	0.2 (0.3)	0.6 (2.2)	0.2 (<0.1)	0.5 (0.5)	0.4 (0.1)	0.6 (0.4)	0.5 (0.2)	0.5 (0.1)	0.6 (0.1)
			Karp-Collapse	<0.1 (0.1)	0.1 (0.4)	0.5 (2.3)	<0.1 (<0.1)	0.3 (0.6)	0.2 (0.2)	0.3 (0.5)	0.2 (0.2)	0.2 (0.2)	0.2 (0.1)
			UCLUST	2 (11.2)	0.4 (1.2)	1 (3.3)	5.7 (11.1)	2.7 (2.2)	2.6 (3.1)	2.9 (3.7)	2.2 (1.1)	2 (1.2)	2.1 (0.4)
			SortMeRNA	2.4 (11.2)	0.5 (1.2)	2.4 (6.7)	8.5 (17.9)	2.6 (1.5)	3 (2.9)	4.3 (4.4)	3.3 (1.4)	3 (1.2)	3 (0.3)
			Kallisto	2.1 (1.7)	5.3 (2.8)	7.1 (3.5)	5.7 (1.4)	6.9 (1.3)	7.5 (1.9)	7.5 (1.2)	7.4 (0.8)	7.3 (0.5)	7.5 (0.4)
			USEARCH61	2.3 (11.2)	0.5 (1.2)	2.4 (6.7)	6.1 (11)	2.6 (1.5)	2.7 (3)	4.1 (4.1)	3 (1.3)	3 (1.3)	2.7 (0.2)
			Mothur NB	30 (32.7)	33.9 (24.7)	32.3 (22)	32.7 (8)	27.3 (9.2)	22.6 (4.3)	17.3 (4.9)	14.2 (2.8)	11.1 (1.9)	9.4 (0.7)
			16S Classifier	23.2 (55.8)	26.5 (40.5)	14.1 (21.7)	23.7 (30.5)	17.5 (12.3)	23.7 (9.7)	24.7 (5.9)	23.2 (5)	23.7 (4)	22.3 (2.1)
			SINTAX	0.3 (1.2)	<0.1 (<0.1)	<0.1 (0.1)	3.0 (9.2)	2.2 (4.9)	0.4 (0.6)	1.2 (3.2)	0.7 (1.1)	0.7 (0.8)	0.4 (0.2)

SE=Single-end, PE=Paired-end

Table 20: **Per order error rates, 100,000 reads**

Read Length	Region	Samples	Method	Shannon Diversity									
				[0,0.7)	[0.7,1.4)	[1.4,2)	[2,2.7)	[2.7,3.4)	[3.4,4.1)	[4.1,4.8)	[4.8,5.5)	[5.5,6.2)	[6.2,6.9]
301bp PE	Full gene	170	Karp	0.3 (0.3)	0.7 (1.2)	0.5 (0.6)	0.6 (0.6)	1 (0.7)	1.1 (0.8)	1.2 (0.5)	1.1 (0.2)	1.2 (0.2)	1.3 (0.1)
			Karp-Collapse	0.1 (0.3)	0.4 (0.9)	0.1 (0.1)	0.3 (0.7)	0.7 (1.4)	0.2 (0.2)	0.6 (0.8)	0.3 (0.2)	0.3 (0.1)	0.3 (0.1)
			UCLUST	<0.1 (0.1)	<0.1 (0.1)	0.2 (0.4)	0.1 (0.1)	0.7 (1.7)	0.1 (0.2)	0.6 (1.1)	0.2 (0.2)	0.5 (0.6)	0.3 (0.1)
			SortMeRNA	0.3 (0.6)	0.4 (0.7)	0.6 (0.8)	0.4 (0.4)	1.2 (1.8)	1.2 (1)	2 (1.8)	1.4 (0.3)	1.7 (0.3)	1.7 (0.1)
			Kallisto	42.3 (36)	67.4 (36.1)	81.8 (32.3)	84.9 (17.8)	96.7 (21)	92 (16.2)	97.9 (17.3)	92.2 (4)	92.3 (8)	88.6 (2.7)
			USEARCH61	<0.1 (0.1)	<0.1 (0.2)	<0.1 (0.1)	<0.1 (<0.1)	0.9 (2)	0.2 (0.2)	0.8 (1.1)	0.3 (0.2)	0.3 (0.2)	0.4 (0.1)
			Mothur NB	4.1 (13.1)	9.6 (14.1)	5.9 (6.7)	5.3 (6.5)	13.2 (11.2)	8.8 (3.7)	8.1 (2.3)	7 (2.3)	6.4 (0.9)	5.1 (0.7)
			16S Classifier	91.3 (66.2)	107.7 (65.1)	108.4 (38)	90 (39.3)	98.2 (23.3)	99.2 (16.9)	90.3 (6.4)	89.8 (11.6)	86.7 (4.4)	86.8 (2.7)
			SINTAX	<0.1 (<0.1)	0.1 (0.3)	0.2 (0.5)	0.6 (1.5)	1.4 (3.4)	0.6 (0.6)	1.1 (1.1)	0.6 (0.4)	0.7 (0.7)	0.6 (0.2)
	V4	190	Karp	0.3 (0.7)	0.7 (0.6)	1 (1.1)	1.1 (1.1)	1.6 (3)	0.9 (0.5)	0.9 (0.2)	1 (0.2)	1.1 (0.1)	1.2 (0.1)
			Karp-Collapse	<0.1 (<0.1)	<0.1 (<0.1)	<0.1 (<0.1)	<0.1 (<0.1)	<0.1 (<0.1)	<0.1 (<0.1)	<0.1 (<0.1)	<0.1 (<0.1)	<0.1 (<0.1)	0.1 (<0.1)
			UCLUST	<0.1 (<0.1)	<0.1 (0.1)	<0.1 (<0.1)	<0.1 (<0.1)	<0.1 (<0.1)	<0.1 (<0.1)	<0.1 (<0.1)	<0.1 (<0.1)	<0.1 (0.1)	0.1 (0.1)
			SortMeRNA	<0.1 (<0.1)	<0.1 (0.1)	<0.1 (<0.1)	0.1 (0.1)	1.2 (3.5)	0.2 (0.1)	0.5 (0.7)	0.5 (0.2)	0.4 (0.1)	0.2 (0.1)
			Kallisto	59.1 (48.6)	98.6 (39.5)	84.3 (30.2)	110.3 (17.5)	105.9 (21.4)	114.4 (10.4)	113.3 (13.4)	120.4 (10.8)	117.4 (6.1)	117.2 (4.3)
			USEARCH61	<0.1 (<0.1)	<0.1 (0.3)	<0.1 (<0.1)	<0.1 (<0.1)	<0.1 (<0.1)	<0.1 (<0.1)	<0.1 (<0.1)	0.1 (0.3)	0.1 (0.2)	0.1 (0.1)
			Mothur NB	40.3 (58.1)	38.3 (41.9)	22 (11.8)	25.9 (11.6)	21.5 (8.8)	21 (4.6)	16.9 (3.9)	15.6 (4.6)	11.3 (1.9)	8.4 (1.5)
			16S Classifier	2.5 (4.8)	25.6 (42.5)	18.2 (31.1)	26 (31.1)	22.9 (26.2)	20.3 (11.4)	26.1 (7.3)	23 (5.3)	20.2 (4.4)	20.4 (2.7)
			SINTAX	<0.1 (<0.1)	0.4 (1.8)	<0.1 (<0.1)	0.1 (0.3)	<0.1 (<0.1)	<0.1 (<0.1)	0.2 (0.5)	0.3 (0.5)	0.6 (0.7)	0.4 (0.2)

SE=Single-end, PE=Paired-end

Table 21: **Per class error rates, 100,000 reads**

Read Length	Region	Samples	Method	Shannon Diversity									
				[0,0.7)	[0.7,1.4)	[1.4,2)	[2,2.7)	[2.7,3.4)	[3.4,4.1)	[4.1,4.8)	[4.8,5.5)	[5.5,6.2)	[6.2,6.9]
75bp SE	Full gene	130	Karp	10.2 (6.3)	6.2 (4.3)	6 (2)	7 (2.5)	5.3 (1.3)	4.3 (0.9)	3.5 (0.9)	2.5 (0.3)	2.6 (0.3)	2.1 (0.3)
			Karp-Collapse	26.6 (12.5)	18 (8.9)	16.2 (4)	17.1 (5.1)	12.8 (2)	10.9 (2)	8.6 (1.5)	6.2 (0.6)	5.7 (0.7)	4.2 (0.4)
			UCLUST	19 (18.1)	10.4 (7.6)	9.7 (3.3)	11.2 (4.6)	7.8 (2.2)	6.4 (1.6)	5.1 (1.1)	3.5 (0.5)	3.2 (0.3)	2.3 (0.2)
			SortMeRNA	22.2 (19.7)	14.3 (8.2)	14.6 (4.4)	16.4 (5.7)	13 (3)	12.1 (1.9)	10.9 (1.4)	8 (0.8)	8.2 (1)	6.8 (0.5)
			Kallisto	20.8 (9.4)	15 (6.1)	13.4 (3.7)	14.5 (4.5)	10.7 (1.6)	9.3 (1.7)	8.2 (1.6)	5.9 (0.5)	5.9 (0.2)	5.2 (0.2)
			USEARCH61	20 (18.7)	11.4 (8.1)	11.1 (3.7)	11.7 (4.4)	8.3 (1.9)	7 (1.5)	5.6 (1.3)	3.8 (0.5)	3.5 (0.3)	2.5 (0.3)
			Mothur NB	35.6 (47.1)	33.6 (24.1)	34 (20.5)	29.8 (12.7)	38.4 (5.7)	30.8 (4.4)	30.6 (4.3)	34.4 (2.7)	34.5 (2.3)	33.1 (1.1)
			16S Classifier	165.6 (33.8)	158.3 (24.1)	137.5 (28.4)	131.5 (35.1)	146 (11.4)	129.8 (11.7)	123.7 (14)	122.9 (8.7)	125.4 (3.1)	125.5 (2.8)
			SINTAX	8.6 (14.3)	7.3 (6.2)	7.8 (3.9)	11.0 (5.2)	9.9 (2.7)	9.8 (2.8)	9.4 (2.0)	8.7 (0.8)	8.9 (0.6)	8.8 (0.5)
	V4	130	Karp	1.4 (1.5)	2.5 (1.5)	3.8 (2)	3.3 (2.7)	3 (1)	2.9 (0.9)	2.7 (0.4)	2.4 (0.6)	2.2 (0.2)	2.2 (0.3)
			Karp-Collapse	9.9 (7.2)	7.9 (4.6)	10.7 (5.7)	7.5 (3.2)	7.6 (1.3)	6.9 (2.2)	5.3 (1.1)	5 (1.7)	3.6 (0.4)	3 (0.3)
			UCLUST	2 (2.3)	2.4 (2.1)	3.9 (3)	2.3 (0.4)	2.7 (0.8)	3.2 (1.2)	2 (0.2)	2.6 (1.1)	1.7 (0.3)	1.6 (0.2)
			SortMeRNA	2.8 (1.8)	3.8 (2.4)	6.7 (2.7)	6.1 (2.1)	6.8 (1.2)	7.7 (2.4)	7.3 (1.5)	7.2 (1.8)	6.9 (0.6)	6.8 (0.5)
			Kallisto	7.9 (6.1)	7.5 (3.1)	9.9 (4.7)	7.9 (2)	7.9 (1)	7.4 (1)	7 (0.7)	6.7 (0.7)	6.8 (0.3)	6.4 (0.4)
			USEARCH61	2.3 (3.2)	2.5 (2)	4.4 (3.7)	2.8 (0.6)	3 (0.7)	3.2 (1.4)	2.5 (0.5)	2.8 (1)	1.9 (0.2)	1.7 (0.2)
			Mothur NB	27.7 (39.5)	23.3 (15.4)	36.3 (20.4)	35.7 (13.4)	35 (11.2)	36.5 (7.4)	33.9 (5.3)	34.1 (3)	32.3 (2.5)	32 (0.9)
			16S Classifier	138.8 (59.2)	119.1 (42.7)	120.8 (33.7)	105 (28.9)	108.9 (13.3)	106.2 (12.8)	94.3 (16)	96.6 (11.2)	94 (3.3)	93.3 (2.1)
			SINTAX	3.4 (3.3)	6.0 (5.0)	9.7 (5.2)	6.7 (2.7)	9.0 (2.0)	8.2 (1.9)	7.1 (1.4)	8.0 (2.0)	6.3 (0.5)	6.3 (0.4)
151bp PE	Full gene	130	Karp	0.5 (1.1)	0.6 (1)	0.7 (0.7)	1 (0.8)	0.9 (0.6)	0.7 (0.3)	1 (0.3)	0.8 (0.2)	0.7 (0.1)	0.7 (0.1)
			Karp-Collapse	0.5 (1.1)	0.6 (1)	0.7 (0.7)	0.8 (0.7)	0.9 (0.7)	0.5 (0.2)	1 (0.4)	0.8 (0.3)	0.7 (0.1)	0.7 (0.1)
			UCLUST	0.4 (0.3)	0.7 (0.4)	0.9 (0.5)	0.9 (0.2)	1.2 (0.3)	1.1 (0.2)	1 (0.2)	1.1 (0.4)	0.9 (0.1)	0.9 (0.1)
			SortMeRNA	1.1 (3)	0.8 (0.5)	1.1 (0.5)	1.2 (0.3)	1.2 (0.4)	1.6 (0.4)	1.7 (0.2)	2 (0.6)	1.9 (0.4)	2.3 (0.2)
			Kallisto	2.2 (2.1)	3.5 (2.2)	4.9 (2.4)	5.9 (1.8)	5.4 (1.4)	5.3 (0.8)	6.1 (1)	5.5 (0.8)	5.5 (0.5)	5.8 (0.2)
			USEARCH61	0.7 (0.7)	1.3 (0.7)	1.6 (0.9)	2.1 (0.7)	1.6 (0.5)	1.6 (0.4)	1.6 (0.4)	1.4 (0.3)	1.3 (0.2)	1.3 (0.2)
			Mothur NB	5.5 (6.2)	7 (6.6)	7.6 (6)	5.8 (5.3)	5.3 (3.3)	4.1 (1.5)	4.4 (1.8)	3.7 (1.2)	3.2 (0.9)	2.7 (0.5)
			16S Classifier	139.2 (59.7)	107.7 (49.1)	97.5 (43.2)	90.9 (34.2)	98 (17.6)	98.8 (17.4)	87.9 (14)	90.9 (9.1)	83.4 (6.8)	86.4 (1.8)
			SINTAX	0.2 (0.3)	0.2 (0.2)	0.3 (0.3)	0.3 (0.1)	0.3 (0.1)	0.3 (0.1)	0.3 (0.1)	0.3 (0.4)	0.2 (0.1)	0.2 (<0.1)
	V4	190	Karp	0.1 (<0.1)	0.2 (0.3)	0.6 (2.2)	0.2 (<0.1)	0.5 (0.5)	0.3 (0.1)	0.5 (0.5)	0.4 (0.2)	0.4 (0.1)	0.4 (0.1)
			Karp-Collapse	<0.1 (0.1)	0.1 (0.4)	0.5 (2.3)	<0.1 (<0.1)	0.3 (0.6)	0.1 (0.1)	0.3 (0.5)	0.2 (0.2)	0.2 (0.1)	0.2 (0.1)
			UCLUST	0.3 (0.9)	0.4 (1.2)	1 (3.3)	5.7 (11.1)	2.1 (1.5)	2.5 (3)	2.6 (2.9)	2 (1.1)	1.8 (1.1)	1.7 (0.4)
			SortMeRNA	0.7 (1.4)	0.5 (1.2)	2.4 (6.7)	8.4 (17.8)	2.6 (1.5)	3 (2.9)	3.9 (3.7)	3 (1.2)	2.6 (1.1)	2.5 (0.3)
			Kallisto	2 (1.7)	5.2 (2.8)	7 (3.5)	5.6 (1.4)	6.6 (1.4)	6.9 (1.7)	7.1 (1.3)	7 (0.8)	6.8 (0.4)	7 (0.3)
			USEARCH61	0.6 (1.4)	0.5 (1.2)	2.3 (6.7)	6 (11)	2.6 (1.5)	2.7 (3)	3.7 (3.5)	2.7 (1.1)	2.5 (1.2)	2.3 (0.3)
			Mothur NB	29.3 (32.6)	25.5 (7)	25.3 (10.8)	29.9 (8.1)	21.6 (8.2)	16.6 (4.2)	12.6 (3.7)	9.1 (1.9)	7.2 (1.5)	5.8 (0.7)
			16S Classifier	22.1 (56)	21.1 (41.2)	10.7 (19.4)	13.4 (14.5)	13.1 (12.3)	19.8 (8.9)	17.8 (6.2)	18 (4.4)	16.4 (3.1)	16.1 (1.8)
			SINTAX	<0.1 (<0.1)	<0.1 (<0.1)	<0.1 (<0.1)	2.9 (8.9)	1.5 (4.3)	0.3 (0.5)	0.6 (1.3)	0.1 (0.1)	0.3 (0.4)	0.2 (0.1)

SE=Single-end, PE=Paired-end

Table 22: **Per class error rates, 100,000 reads**

Read Length	Region	Samples	Method	Shannon Diversity									
				[0,0.7)	[0.7,1.4)	[1.4,2)	[2,2.7)	[2.7,3.4)	[3.4,4.1)	[4.1,4.8)	[4.8,5.5)	[5.5,6.2)	[6.2,6.9]
301bp PE	Full gene	170	Karp	0.3 (0.3)	0.7 (1.2)	0.5 (0.6)	0.6 (0.5)	0.7 (0.4)	0.9 (0.7)	0.7 (0.2)	0.8 (0.1)	0.8 (0.2)	0.8 (0.1)
			Karp-Collapse	0.1 (0.2)	0.3 (0.9)	0.1 (0.1)	0.3 (0.6)	0.1 (0.1)	0.2 (0.2)	0.1 (0.1)	0.2 (0.1)	0.2 (0.1)	0.1 (<0.1)
			UCLUST	<0.1 (<0.1)	<0.1 (<0.1)	<0.1 (<0.1)	<0.1 (<0.1)	<0.1 (<0.1)	<0.1 (<0.1)	<0.1 (<0.1)	<0.1 (<0.1)	<0.1 (<0.1)	<0.1 (<0.1)
			SortMeRNA	0.2 (0.2)	0.3 (0.6)	0.4 (0.7)	0.2 (0.2)	0.4 (0.1)	0.8 (0.9)	0.7 (0.1)	0.9 (0.2)	1 (0.2)	1.3 (0.1)
			Kallisto	42 (36)	66.4 (37.6)	79.9 (32.1)	83.3 (17.9)	91.8 (23.1)	87.5 (16.9)	91.5 (19.1)	84.8 (7.7)	85.7 (7.6)	82.2 (5.4)
			USEARCH61	<0.1 (<0.1)	<0.1 (<0.1)	<0.1 (<0.1)	<0.1 (<0.1)	<0.1 (<0.1)	<0.1 (<0.1)	<0.1 (<0.1)	<0.1 (0.1)	<0.1 (<0.1)	<0.1 (<0.1)
			Mothur NB	4.1 (13.1)	7.4 (13.6)	4.4 (6.6)	3.4 (4.9)	7.9 (10.6)	6.2 (2.8)	4.4 (1.5)	4 (2.1)	3.6 (0.8)	2.9 (0.6)
			16S Classifier	86.1 (65.9)	96.4 (63.8)	97.5 (33.9)	75.5 (32)	84.9 (19.8)	84.9 (15)	74.5 (8.2)	75.1 (10.3)	72.4 (3.8)	72.4 (2.6)
			SINTAX	<0.1 (<0.1)	<0.1 (<0.1)	<0.1 (<0.1)	<0.1 (<0.1)	<0.1 (<0.1)	0.1 (0.4)	0.1 (0.3)	0.1 (0.1)	<0.1 (0.1)	<0.1 (<0.1)
	V4	190	Karp	0.3 (0.7)	0.6 (0.6)	0.9 (1.1)	1 (1.1)	1.6 (3)	0.8 (0.5)	0.7 (0.2)	0.7 (0.2)	0.8 (0.2)	0.8 (0.1)
			Karp-Collapse	<0.1 (<0.1)	<0.1 (<0.1)	<0.1 (<0.1)	<0.1 (<0.1)	<0.1 (<0.1)	<0.1 (<0.1)	<0.1 (<0.1)	<0.1 (<0.1)	<0.1 (<0.1)	<0.1 (<0.1)
			UCLUST	<0.1 (<0.1)	<0.1 (<0.1)	<0.1 (<0.1)	<0.1 (<0.1)	<0.1 (<0.1)	<0.1 (<0.1)	<0.1 (<0.1)	<0.1 (<0.1)	<0.1 (<0.1)	<0.1 (<0.1)
			SortMeRNA	<0.1 (<0.1)	<0.1 (<0.1)	<0.1 (<0.1)	0.1 (<0.1)	1.2 (3.5)	0.2 (<0.1)	0.2 (<0.1)	0.3 (0.1)	0.3 (0.1)	0.2 (<0.1)
			Kallisto	58.9 (48.5)	95.1 (42.1)	81.9 (31.7)	107.7 (16)	103.4 (23.8)	110.3 (13.7)	109.6 (14.7)	114.6 (14.8)	113.5 (7.3)	111.5 (5.3)
			USEARCH61	<0.1 (<0.1)	<0.1 (<0.1)	<0.1 (<0.1)	<0.1 (<0.1)	<0.1 (<0.1)	<0.1 (<0.1)	<0.1 (<0.1)	<0.1 (<0.1)	<0.1 (<0.1)	<0.1 (<0.1)
			Mothur NB	39.2 (57.3)	35.5 (38.5)	17.4 (9.9)	21.3 (8.6)	19.1 (7.8)	16.3 (4.5)	12.1 (2.5)	10.1 (3)	6.8 (1.5)	4.8 (1.1)
			16S Classifier	1.8 (4.9)	11.4 (31.7)	13.6 (28.7)	18.2 (28.6)	14.8 (24.3)	13.1 (10.7)	16 (7.6)	15.7 (4.5)	13.8 (3.9)	14.3 (1.7)
			SINTAX	<0.1 (<0.1)	<0.1 (<0.1)	<0.1 (<0.1)	<0.1 (<0.1)	<0.1 (<0.1)	<0.1 (<0.1)	0.2 (0.5)	<0.1 (<0.1)	0.2 (0.3)	0.1 (0.1)

SE=Single-end, PE=Paired-end
