# Supplementary figures and images for: Using pseudoalignment and base quality to accurately quantify microbial community composition
Source: PLoS Comput Biol. 2018 Apr 16;14(4):e1006096. doi: 10.1371/journal.pcbi.1006096 (PMC5945057; doi:10.1371/journal.pcbi.1006096)

**A****Read Length**

- 75 Single-end
- 151 Paired-end
- 301 Paired-end

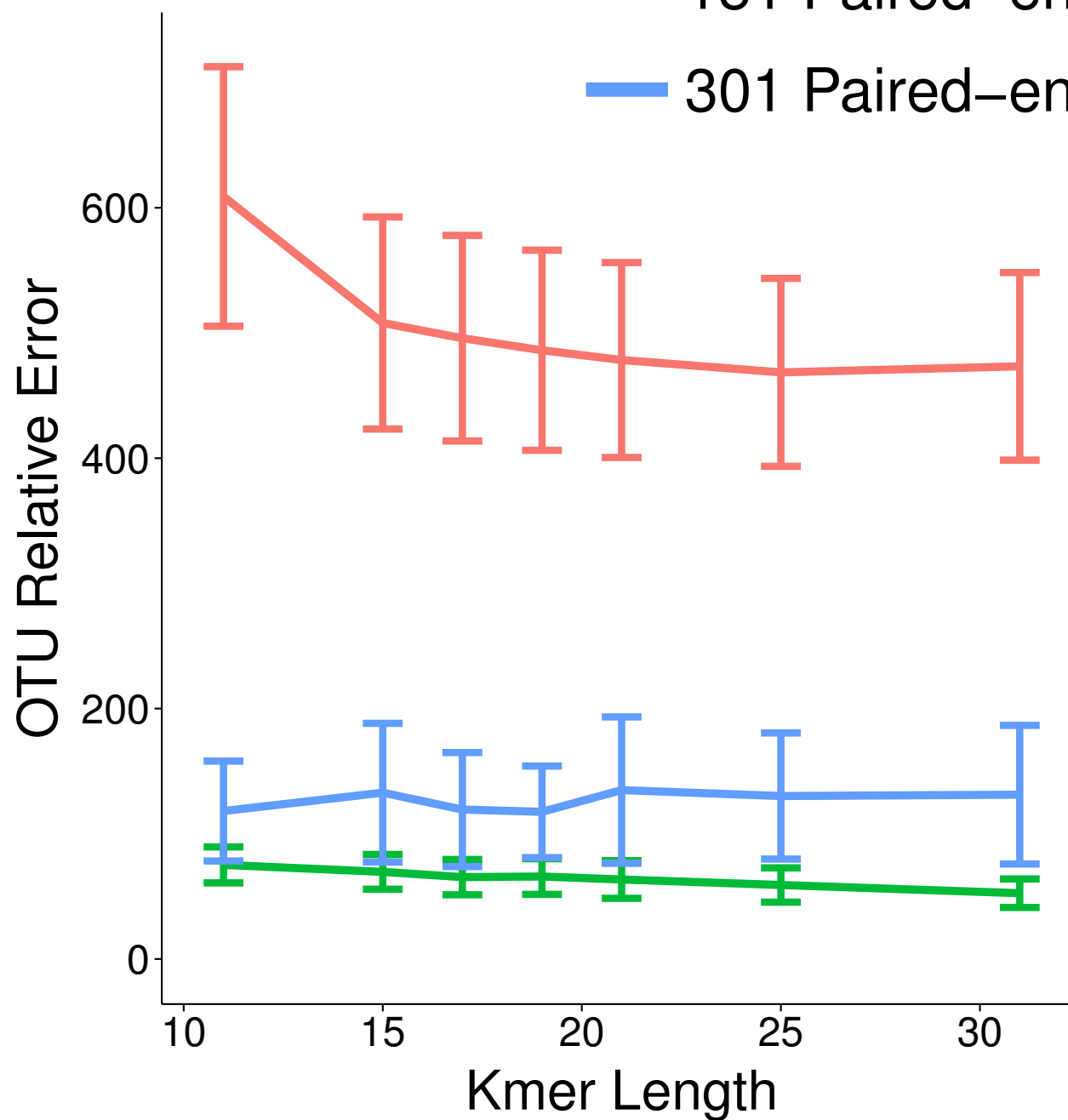**B**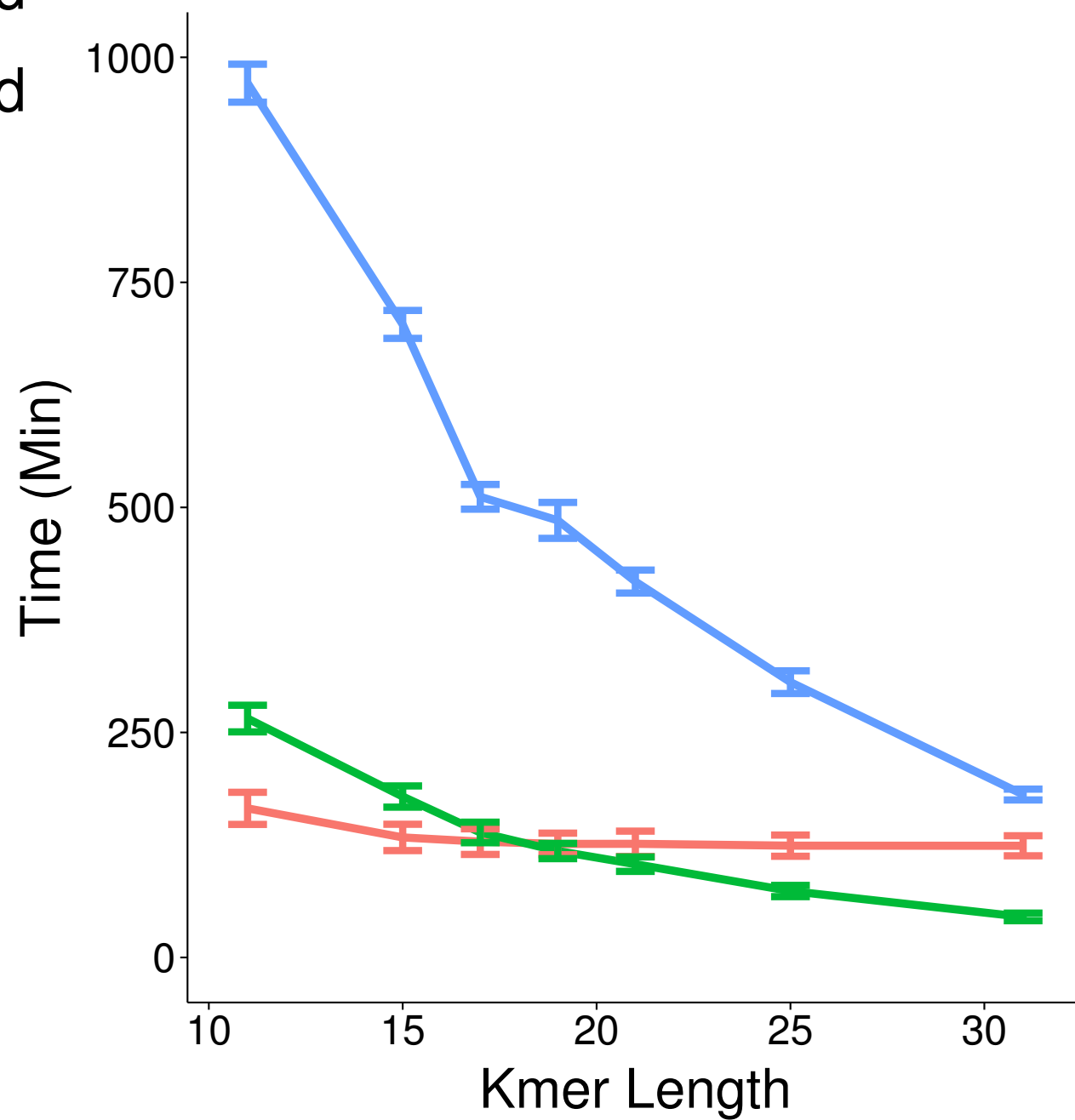

Supplement: S1 Fig — Impact of k-mer length on Karp performance. Pseudoalignment indexes constructed using different k-mer lengths were used to classify 30 previously analyzed samples selected to cover a full range of Shannon Diversities. For each of 75bp, 151bp, and 301bp reads 10 samples of 1,000,000 reads were analyzed. (A) The average error values with 95% confidence intervals for each read length. (B) Average run times using 12-cores in parallel. (PDF) [file pcbi.1006096.s005.pdf]

**A**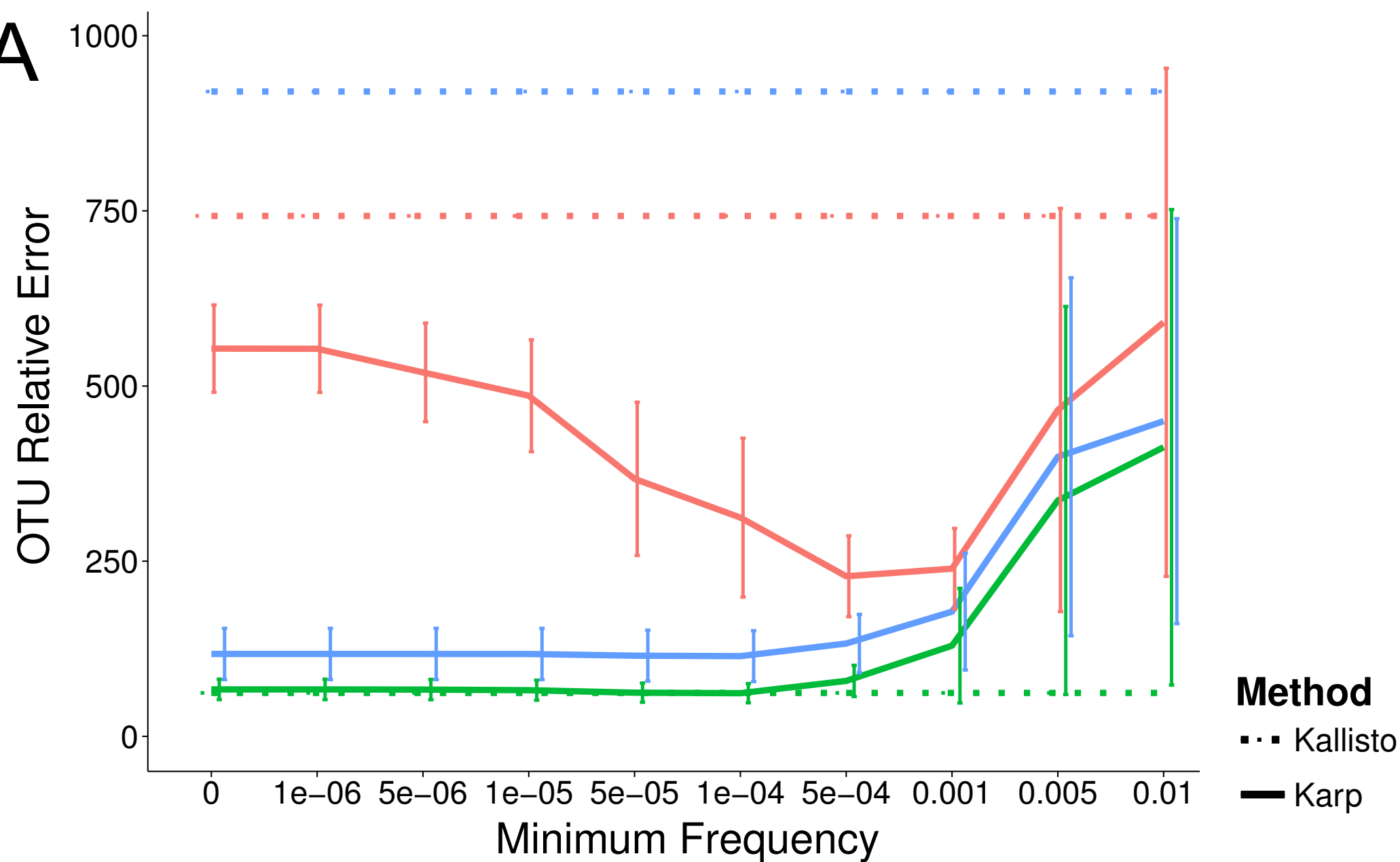**B**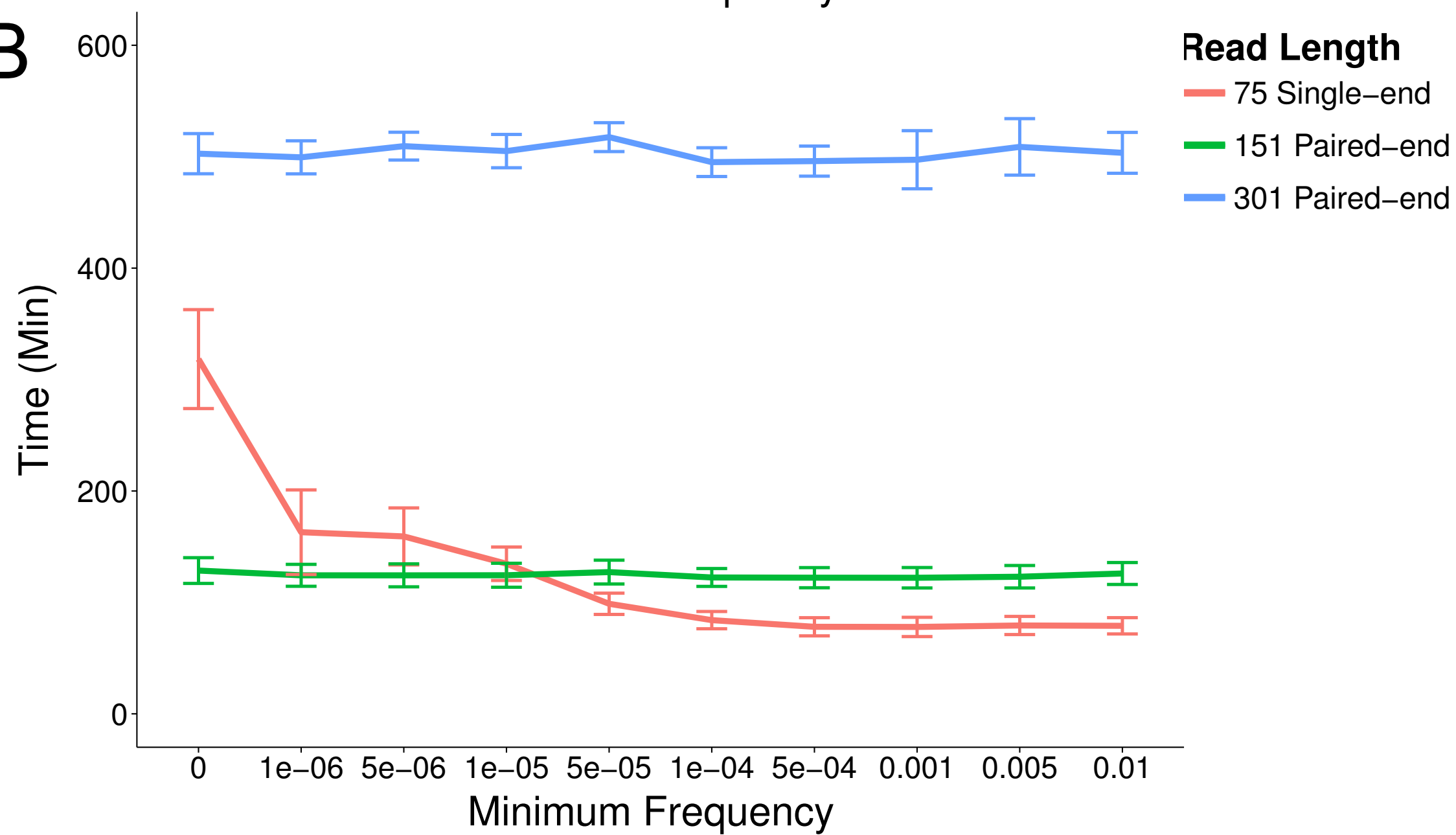

Supplement: S2 Fig — Karp uses an EM algorithm to estimate the relative frequencies of reference sequences in a pooled sample. During the EM process a minimum frequency threshold can be applied that removes references with frequencies below this threshold. Set at a low frequency, the threshold helps remove spurious findings and improves accuracy, particularly for shorter reads. At higher frequencies the threshold removes references actually present in the sample and lowers accuracy. In this figure different thresholds are applied during quantification of 30 previously analyzed samples selected to cover a full range of Shannon Diversities. K-mers of length 19 were used for these analyses. For lengths of 75bp, 151bp, and 301bp 10 samples were analyzed. (A) The average error values with 95% confidence intervals for each read length. (B) Average run times using 12-cores in parallel. For shorter reads, increasing the threshold reduces the number of EM iterations required to converge and decreases run-time. (PDF) [file pcbi.1006096.s006.pdf]

OTU Relative Error

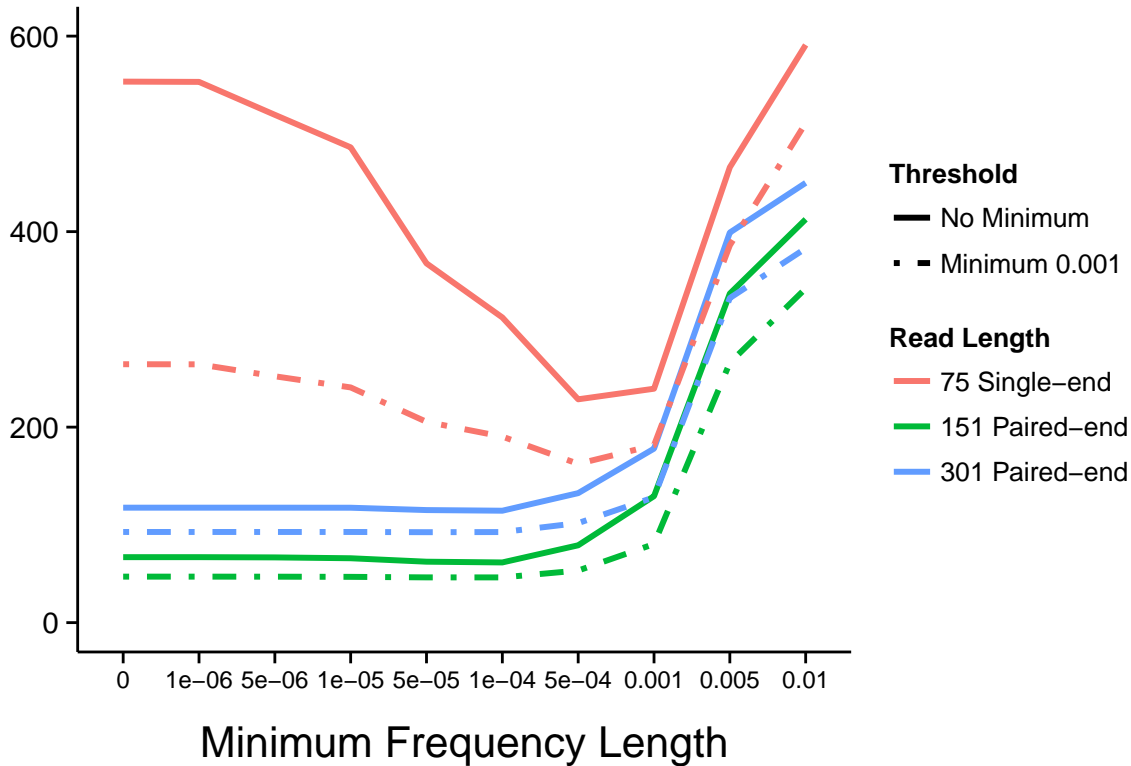

Supplement: S3 Fig — The impact of the EM frequency threshold is smaller when analyzing error in the estimates of more common references. Solid lines present the error calculated using all references classified, dashed lines give the error when only references with an actual or estimated frequency above >0.1%, a cut-off used frequently in this study. In such cases the chosen frequency threshold is less important. (PDF) [file pcbi.1006096.s007.pdf]

Shannon Diversity

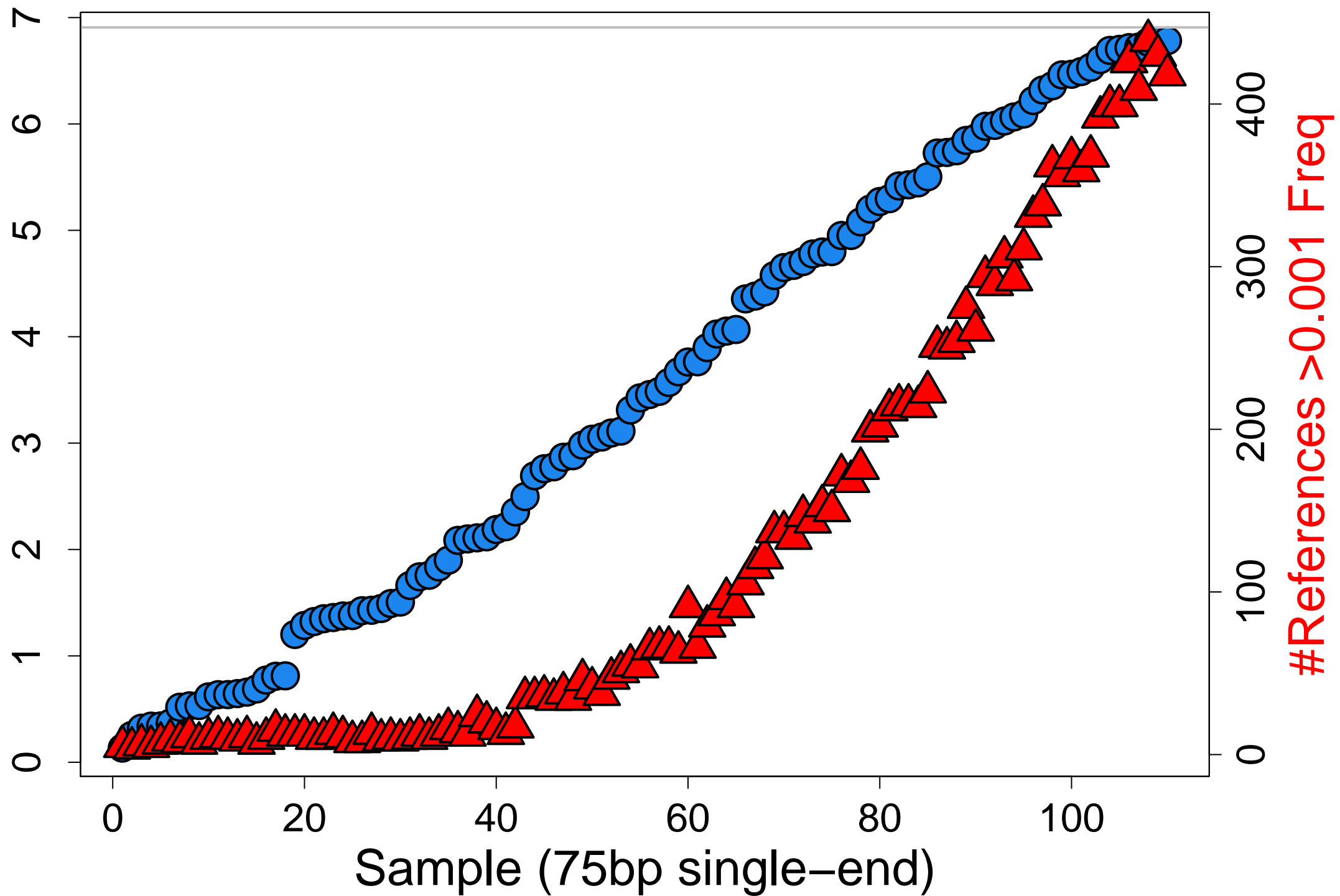

Supplement: S4 Fig — Each simulated dataset contains reads from a mixture of 1,000 reference sequences (each an operational taxonomic unit: OTU). The frequencies at which reads were generated from contributing references were varied to create datasets with a range of Shannon Diversity. As diversity increases the frequency distribution begins to approach a uniform distribution. (PDF) [file pcbi.1006096.s008.pdf]

**A**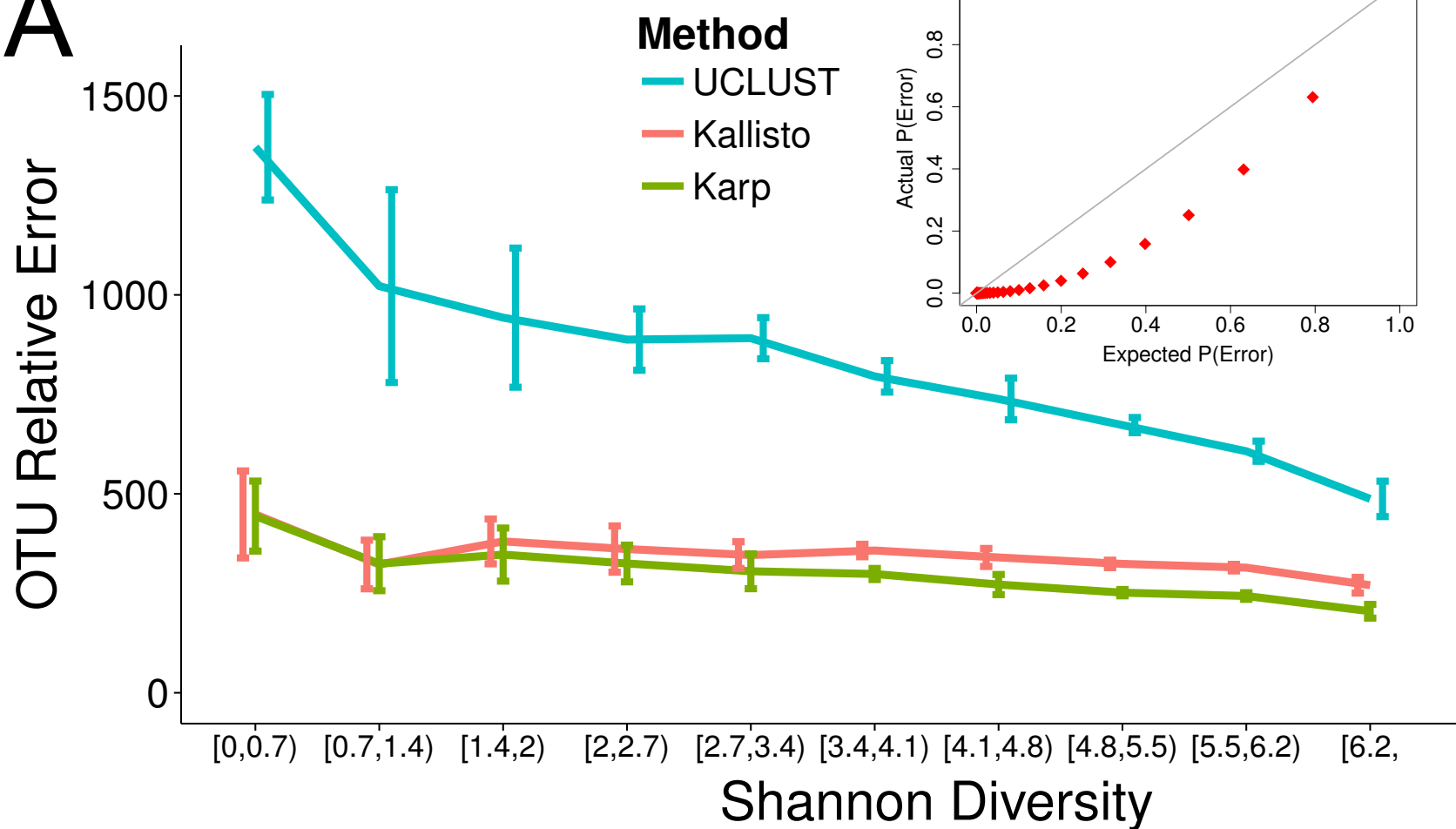**B**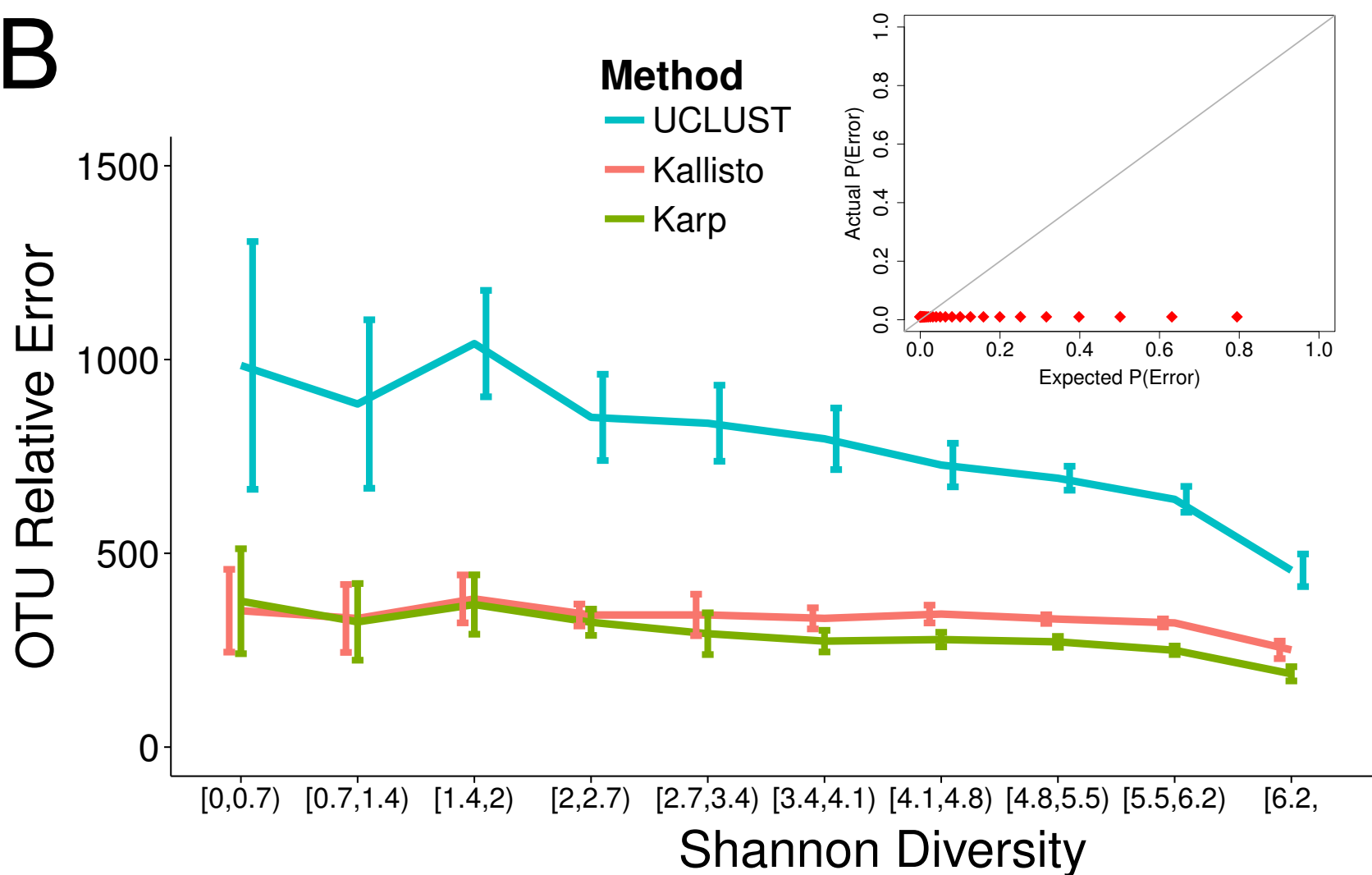

Supplement: S5 Fig — Impact of assumption that base-quality scores accurately represent probability of sequencing error. For two different models of sequencing error we simulated 50 samples and classified them with Karp, Kallisto, and UCLUST/USEARCH. Each method is represented by a different colored line, and bars represent 95% confidence intervals (A) In our first model the true rate of sequencing error varied with the base-quality score, but was smaller than Karp’s model assumes. (B) In our second model, errors were distributed uniformly at 1% of bases in each read, independent of whatever base-quality score was assigned. (PDF) [file pcbi.1006096.s009.pdf]

A

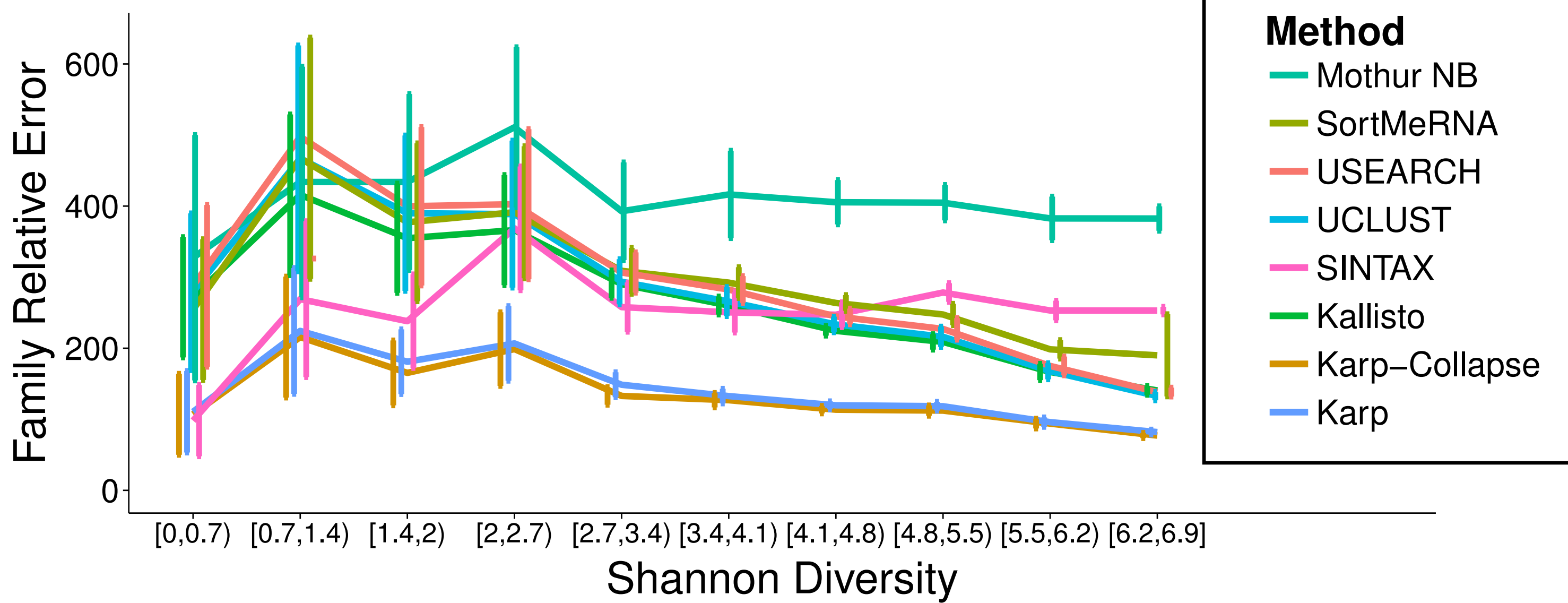

B

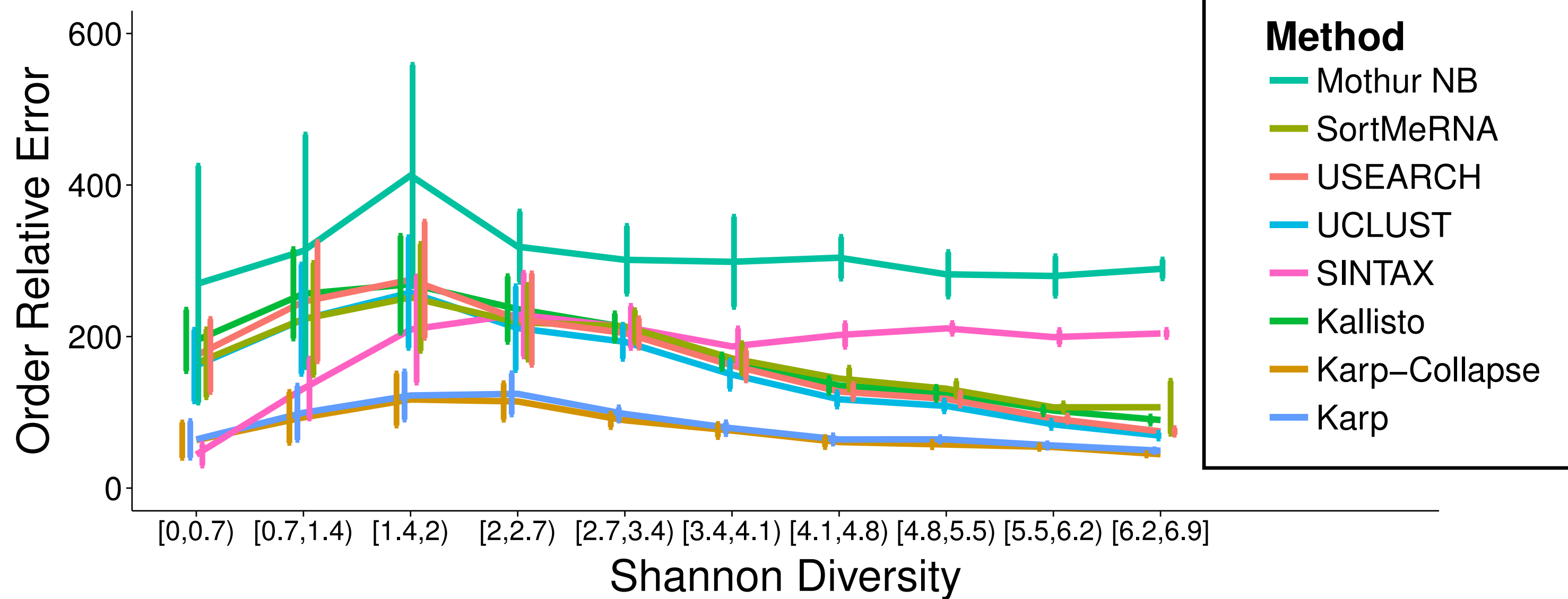

C

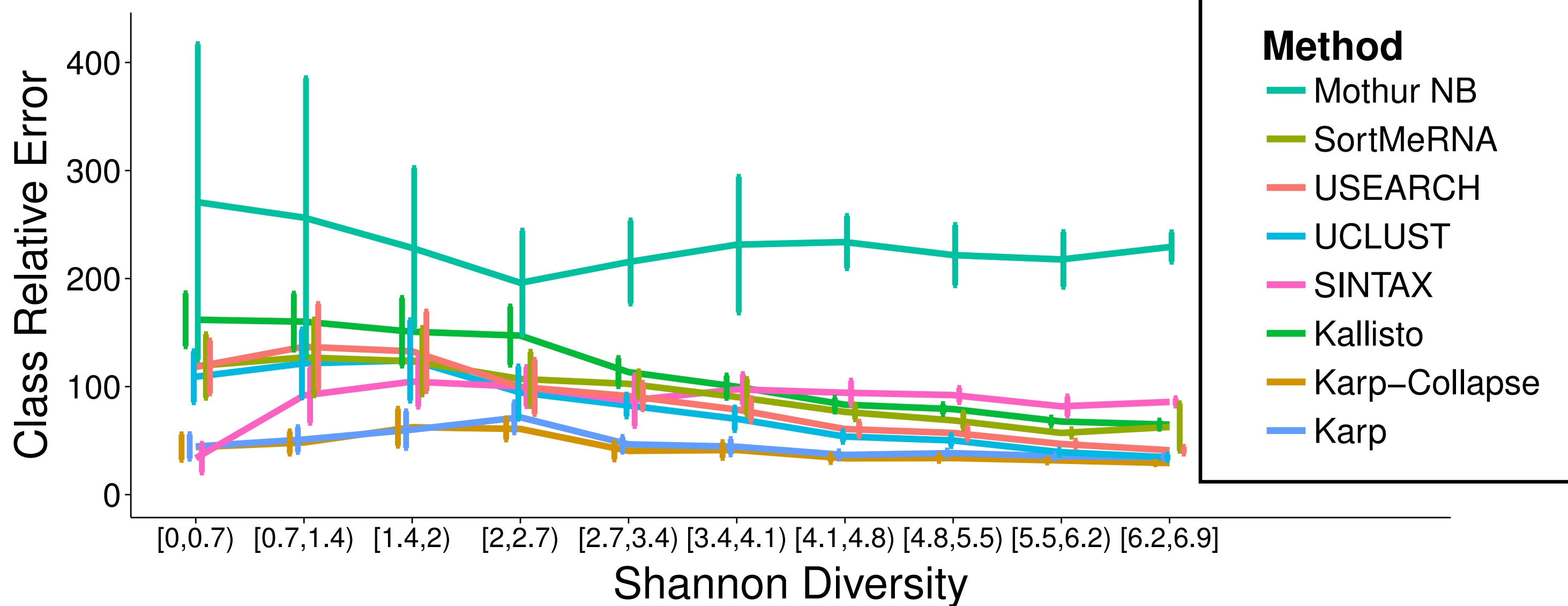

Supplement: S6 Fig — Average absolute error and 95% confidence intervals from the taxonomic quantification of 110 simulated samples, each comprised of 1,000,000 75bp paired-end reads. Taxonomy was classified using Karp, Kallisto, SINTAX, UCLUST, USEARCH, SortMeRNA, and the Naive Bayes implemented in Mothur. Counts were aggregated for OTUs classified in the same (A) family, (B) order, or (C) class and taxa with a frequency >0.1% were compared to their true counts. (PDF) [file pcbi.1006096.s010.pdf]
