# Supplementary material for: Using pseudoalignment and base quality to accurately quantify microbial community composition
Source: PLoS Comput Biol. 2018 Apr 16;14(4):e1006096. doi: 10.1371/journal.pcbi.1006096 (PMC5945057; doi:10.1371/journal.pcbi.1006096)

	Total associations			Distinct genera found to be associated			Proportion associations found by another method		
FDR	0.01	0.05	0.1	0.01	0.05	0.1	0.01	0.05	0.1
Karp Full	4	40	121	4	26	45	0.5	0.33	0.33
Karp Collapse	12	53	131	11	32	51	0.17	0.28	0.32
Kallisto	9	38	95	9	27	42	0.22	0.29	0.26
UCLUST	8	42	120	6	18	34	0.125	0.26	0.2

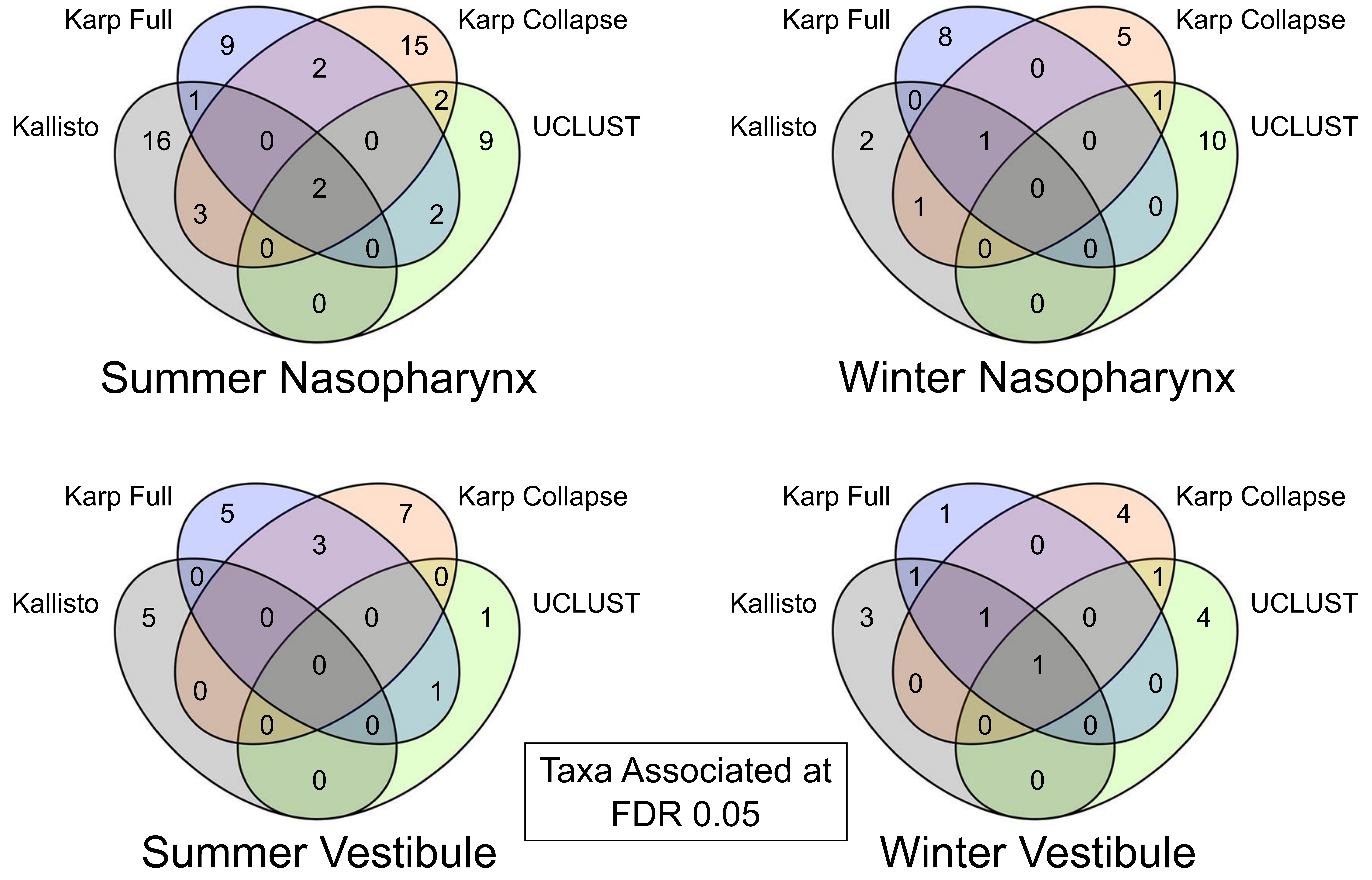

Supplement: S7 Fig — Associations between the relative abundances of 369 genera level taxa in 332 16S sequencing samples and their host genomes. The 16S samples were drawn from brushings of two tissues, the nasopharynx and nasal vestibule, during either the summer or the winter. 3,161,460 SNPs were tested for association with the relative genera abundances estimated by four methods: Karp, Karp-Collapse, Kallisto, and UCLUST. There was greater microbiome diversity in the summer samples, particularly those from the nasopharynx, and this was reflected in a greater number of associations across all classification methods. At FDR thresholds of 0.01, 0.05, and 0.1 the Karp-Collapse method detected the most independent associations, and with the most distinct genera. Additionally, the Karp algorithms and Kallisto also shared a larger group of common associations with each other than they did with UCLUST. (PDF) [file pcbi.1006096.s011.pdf]
